# Benzobicyclo[3.2.1]octene Derivatives as a New Class of Cholinesterase Inhibitors

**DOI:** 10.3390/molecules25214872

**Published:** 2020-10-22

**Authors:** Tena Čadež, Ana Grgičević, Ramiza Ahmetović, Danijela Barić, Nikolina Maček Hrvat, Zrinka Kovarik, Irena Škorić

**Affiliations:** 1 Institute for Medical Research and Occupational Health, Ksaverska Cesta 2, 10000 Zagreb, Croatia; tcadez@imi.hr (T.Č.); nmacek@imi.hr (N.M.H.); 2 Department of Organic Chemistry, Faculty of Chemical Engineering and Technology, University of Zagreb, Marulićev trg 19, 10000 Zagreb, Croatia; aratkov@fkit.hr (A.G.); ramiza.ahmetovic96@gmail.com (R.A.); 3 Division of Physical Chemistry, Rudjer Bošković Institute, Bijenička Cesta 54, 10000 Zagreb, Croatia; dbaric@irb.hr

**Keywords:** acylation, benzobicyclo[3.2.1]octane/octene, benzylamines, cholinesterase, epoxidation, oximes

## Abstract

A library of amine, oxime, ether, epoxy and acyl derivatives of the benzobicyclo[3.2.1]octene were synthesized and evaluated as inhibitors of both human acetylcholinesterase (AChE) and butyrylcholinesterase (BChE). The majority of the tested compounds exhibited higher selectivity for BChE. Structural adjustment for AChE seems to have been achieved by acylation, and the furan ring opening of furo-benzobicyclo[3.2.1]octadiene results for compound **51** with the highest AChE affinity (IC_50_ = 8.3 µM). Interestingly, its analogue, an oxime ether with a benzobicyclo[3.2.1]-skeleton, compound **32** was one of the most potent BChE inhibitors in this study (IC_50_ = 31 µM), but not as potent as *endo-***43**, an ether derivative of the benzobicyclo[3.2.1]octene with an additional phenyl substituent (IC_50_ = 17 µM). Therefore, we identified several cholinesterase inhibitors with a potential for further development as potential drugs for the treatment of neurodegenerative diseases.

## 1. Introduction

Acetylcholinesterase (AChE, EC 3.1.1.7) and butyrylcholinesterase (BChE, EC 3.1.1.8), as they control the acetylcholine ambient in the synapsis, are pharmacologically relevant enzyme targets in neurodegenerative disorders [1,2]. AChE has an essential physiological role in the body as it controls the transmission of nerve impulses in the cholinergic synapses of the central and peripheral nervous system by hydrolysis of the neurotransmitter acetylcholine. Loss of neurons results in a significant reduction in AChE and an increase in BChE levels in the brain [3,4]. BChE, even though it is not physiologically essential, serves as a co-regulator of cholinergic neurotransmission capable of catalyzing the hydrolysis of acetylcholine [5]. Except for cholinergic activity, cholinesterases are associated with amyloid plaques and neurofibrillary tangles which together with the aggregation of toxic amyloid-β peptide lead to the dysfunction and apoptosis of neurons [6,7,8,9,10]. Therefore, compounds acting as inhibitors of cholinesterase could be considered as potential drugs in Alzheimer’s disease (AD) treatment, other neurological disorders and therapeutics [11,12,13,14,15]. Due to the composition of their active site, two enzymes can differ in selectivity and specificity for inhibitor binding [16,17,18]. Moreover, it has been well-documented that BChE reacts with a wider range of ligands than AChE and is less selective [19,20,21,22,23,24,25,26].

**Figure 1:**
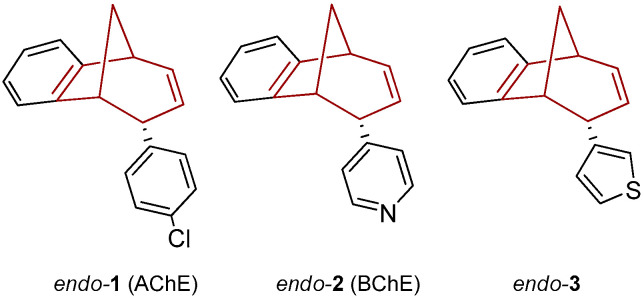
Photoproducts as potent inhibitors of BChE and AChE [24].

Recently we prepared photoproducts, shown in Figure 1, and probed the cholinesterase inhibition [27,28]. Compounds *endo*-**1** and *endo*-**2** were the most potent inhibitors of AChE (IC_50_ = 17.5 µM) and BChE (IC_50_ = 8.8 µM), respectively, among the tested compounds. All of the tested photoproducts exhibited a binding preference for BChE. In comparison to huperzine A, a selective BChE inhibitor (with similar basic methano-bridged aryl-bicyclo[3.3.1]octadiene skeleton), compound *endo*-**2** was about five times more potent as a BChE inhibitor [10].

**Figure 2:**
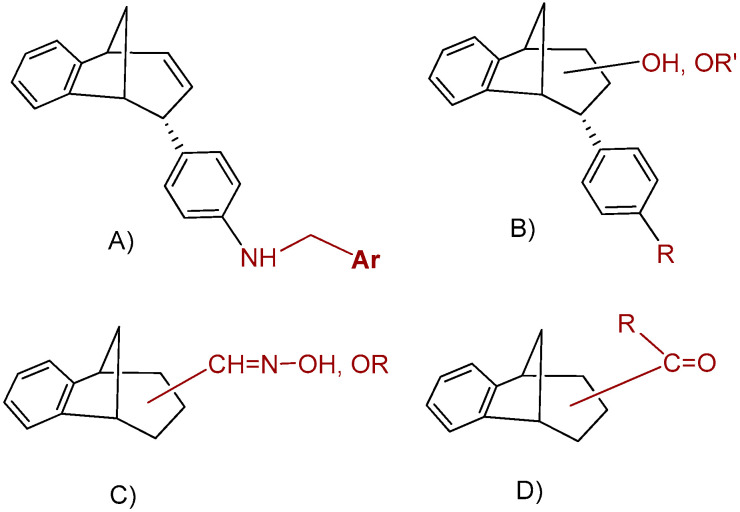
Functionalization of the benzobicyclo[3.2.1]octadiene skeleton employed in this study: (**A**) amines; (**B**) epoxides, alcohols and ethers; (**C**) oximes and oxime ethers; and (**D**) acyl derivatives.

The present study focused on the additional functionalization of the primary photoproducts to obtain new amines, epoxides, alcohols and ethers (Figure 2A,B), or to find new oximes, oxime ethers or acyl derivatives (Figure 2C,D). The mandatory postulate was high yield/productivity in the photochemical reaction. That strategy resulted in a wide range of related compounds, four groups of molecules with different functionalities and a common methano-bridged benzobicyclo[3.2.1]octadiene skeleton found in huperzine A, many biologically active natural sesquiterpenoids [29] or similar to bicyclo[3.2.1]octane benzylamines proven as potential neurokinin–1 antagonist drugs [30]. Compounds were evaluated as inhibitors of both human AChE and BChE.

## 2. Results and Discussion

### 2.1. Benzylic Amines with the Benzobicyclo[3.2.1]-Skeleton

**Scheme 1:**
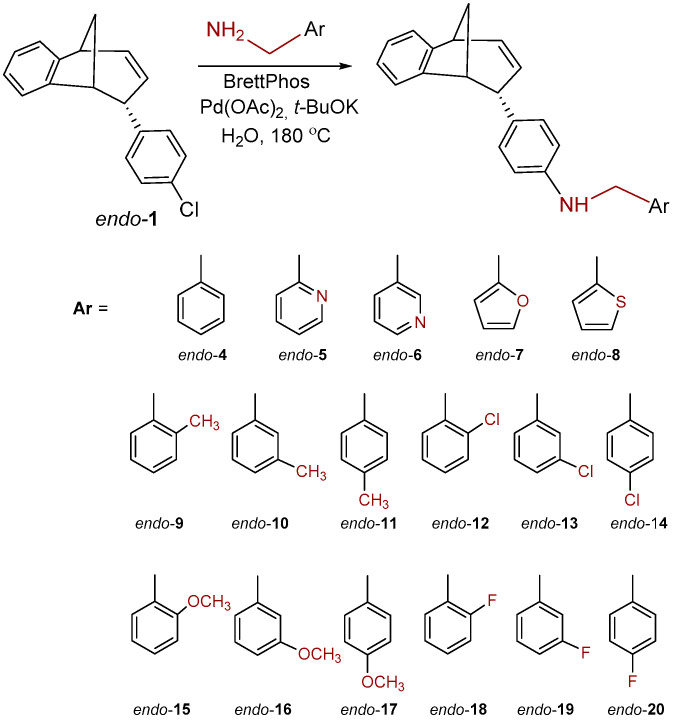
Synthesis of benzobicyclo[3.2.1]octadiene benzylamines, *endo*-**4**–**20**.

Chloro-substituted *endo*-**1** (Scheme 1) was chosen as a suitable substrate for the Buchwald−Hartwig amination reaction. Compound *endo*-**1** was obtained in one photochemical step according to the described method from the previously prepared *o*-vinylphenyl substituted butadienes [31]. The Buchwald−Hartwig amination successfully gave amino substituted benzobicyclo[3.2.1]octadienes *endo*-**2**–**18** (Scheme 1) at 21–88% yields [32].

**Figure 3:**
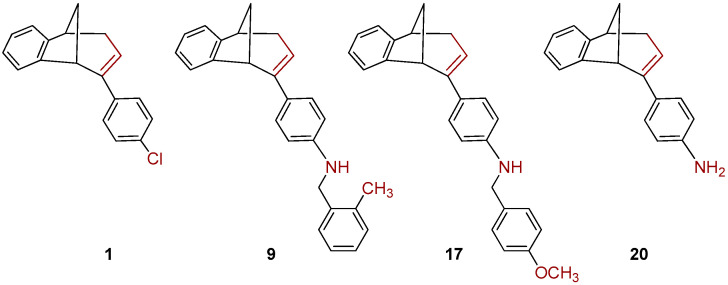
The rearranged products **1**, **9**, **17** and **20** obtained during amination of *endo*-**1**.

The benzylamines obtained in good yields were tested as potential cholinesterase inhibitors as well as **1**, **9**, **17** and **20**, rearranged products obtained during amination of *endo*-**1** with different benzylamines (Figure 3).

We tested 18 compounds described above as reversible inhibitors of human AChE and BChE (Table 1), but it seems that benzobicyclo[3.2.1]octadiene benzylamines were poor inhibitors of both cholinesterases. The exception was *endo*-**10**, a compound with methyl on the benzylamine substituent in *meta* position, which seems important for the inhibition and binding interactions between BChE and benzylamine. The rearranged products (**1**, **9**, **17** and **20**; Figure 3) were more potent BChE inhibitors than *endo*-benzylamines of the bicyclo[3.2.1]octane skeleton. However, none of the compounds (≤100 µM) showed potency to inhibit AChE. Unfortunately, in the case of AChE, due to the solvent interference, we could not use a higher concentration.

### 2.2. Oxime Derivatives Possessing Benzobicyclo[3.2.1]-Skeleton

**Scheme 2:**
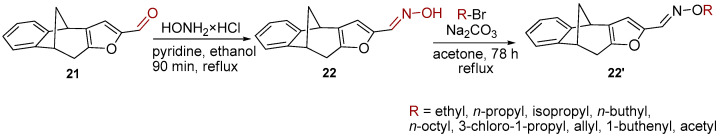
Synthetic pathway toward a new oxime and oxime ethers **22** and **22′**.

**Figure 4:**
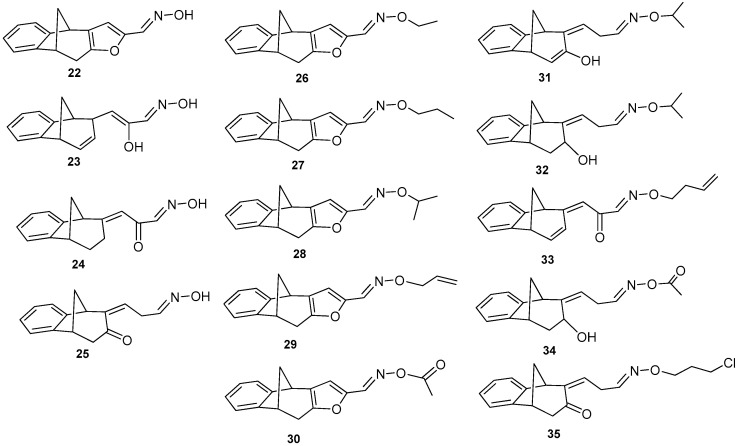
Obtained oximes **22**–**25** and oxime ethers **26**–**35**.

Further functionalization of the basic skeleton (Scheme 2) was performed according to the synthesis of the formyl derivative [33], and a previously published study that combined docking and density functional theory [34]. From the formyl derivative **21**, the corresponding bicyclic oxime **22** was synthesized as a crucial substrate for further synthesis of oxime ethers **22′**. Along with the expected oxime **22** and oxime ethers **26**–**30**, due to opening of the furan ring two classes were formed—opened oximes **23**–**25** and oxime ethers **31**–**35** (Figure 4).

Oxime derivatives possessing a benzobicyclo[3.2.1]-skeleton (Figure 4) were prepared at sufficient amounts and were tested as cholinesterase inhibitors. According to the results given in Table 2, three different functionalities of compounds including oximes, oxime ethers with/without furan ring seem highly related to inhibition potency. The lead inhibitors could be detected in each group, oxime **22** (IC_50_ = 25 ± 6 µM), oxime ether **28** with isopropyl group and furan ring (IC_50_ = 24 ± 3 µM) and oxime ether **32** with an opened furan ring and isopropyl functionality (IC_50_ = 31 ± 4 µM). As for the presence of the furan ring, it seems important only for the inhibition of BChE with oxime derivatives **22**, **23**, **24**, and **25**. In other words, IC_50_ for the opened oxime ether compounds was in the same range like IC_50_ for furan-containing oxime ethers, as shown for compounds **28** and **32**. However, IC_50_ decreased for compounds with more saturated bicyclic moiety, e.g., octadiene **31** was a weaker inhibitor than octene analogue **32**. Compounds **27** and **29** differ only in the saturation of their chain substituent and again propenyl derivative **29** inhibited slightly less potently than its propyl analogue **27**. Moreover, out of opened oxime ether derivatives (compounds **31**–**35**), butenyl derivative **33** was the less potent inhibitor. Therefore, the saturation of a compound can maybe play a crucial role for their beneficial interaction inside of the BChE active gorge. In case of AChE, none of the compounds were able to inhibit more than 20% of enzyme with 200 µM concentration.

### 2.3. Epoxides, Alcohols and Ethers with Benzobicyclo[3.2.1]-Skeleton

**Scheme 3:**
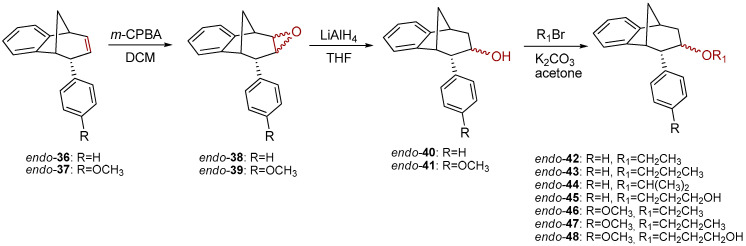
Synthetic path to novel epoxides, alcohols and ethers.

**Figure 5:**
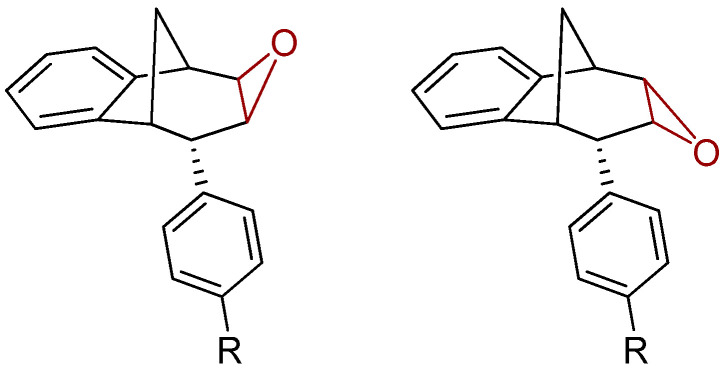
Representation of the stereoisomers of the obtained epoxides *endo*-**38** (R = H) and *endo*-**39** (R = OCH_3_).

Further functionalization of the benzobicyclo[3.2.1]octadiene skeleton was made through the synthesis of novel epoxy derivatives, suitable substrates for novel alcohols and corresponding ethers. It is worth mentioning that functionalization utilized the addition reaction to the free double bond on the basic methano-bridged bicyclic skeleton. As it is known, epoxides represent three-membered cyclic ethers of high reactivity and one way of synthesizing them is by epoxidation using peroxy acids, which have an electrophilic oxygen atom, in a suitable solvent. This reaction represents a stereospecific syn-addition to the double bond [35,36,37]. In this study, the reaction pathway to novel epoxides, alcohols and ethers with the specific geometry (Scheme 3) began by obtaining corresponding epoxides of photoproducts *endo*-**36** and *endo*-**37**, and via alcohols the pathway led to desired ethers with a benzobicyclo[3.2.1]octadiene structure. More specifically, the reaction of endo-**36** and endo-**37** with *meta*-chloroperbenzoic acid (*m*-CPBA) in dichloromethane (DCM) as solvent gave the corresponding epoxides *endo*-**38** (61%) and *endo*-**39** (50%). Although two stereoisomers could be obtained regarding the oxygen position at the three-membered ring relative to the plane of the larger ring in the bridged bicyclic system (Figure 5), we were unable to determine the exact stereochemistry of *endo*-**38** and *endo*-**39** by spectroscopic analysis (see experimental and SI, Figures S1–S19). Therefore, the stereochemistry was predicted by DFT calculations (See SI).

The ring opening was carried out by reacting epoxides *endo*-**38** and *endo*-**39** and LiAlH_4_ in tetrahydrofuran (THF; Scheme 3) as it is known that epoxides due to tension of the three-membered ring are highly reactive. The resulting products of the reaction with reducing agent were alcohols that can theoretically have four isomers: two regioisomers, and within them two more stereoisomers. According to the literature, the most likely isomer, resulting from the epoxide opening product, is a regioisomer with an hydroxy (OH) group on the middle carbon atom of the larger ring in the bridged bicycle system [38]. Finally, the conversion of alcohol into different ethers was carried out by a simple reaction of the alcohol with corresponding alkyl bromides and potassium carbonate in acetone (Scheme 3). The isolated products (20%–50% yields) were characterized by spectroscopic methods (see experimental and SI, Figures S1–S19).

Results on the biological activity of obtained epoxides, alcohols and ethers with a benzobicyclo[3.2.1]-skeleton in Table 3 show higher efficiency in the inhibition of both AChE and BChE than their counterparts. Epoxide *endo*-**38** as a highly reactive compound has proven to be a potent inhibitor for AChE even though its potency was still 3-fold lower than for BChE. An opening of the epoxide ring resulted in derivatives with higher selectivity of BChE, but the IC_50_ was only possible to evaluate for ether, *endo*-**43**, or alcohol, *endo*-**45**, compounds without a substituent on the endo-oriented benzene ring. It seems that a methoxy group in the para position on the phenyl substituent (*endo*-**46** and *endo*-**47**) disturbed beneficial interactions for inhibition by a methoxy group as an electron-donating group. Interestingly, a study by Mohammadi-Farani et al. with similar compounds containing a methoxy group in the *meta* position on the phenyl ring, reported an increase of the inhibition potency for AChE [39]. Therefore, in the case of *endo*-**46** and *endo*-**47**, their para-methoxy group probably could not achieve effective stabilization in the active site.

#### The Modelling of an Epoxide Ring Opening

To predict with more certainty which of the four possible alcohol isomers is dominant among the products, we computationally modelled the reaction of an epoxide opening in molecule *endo*-**38**. Calculations were performed at the (SMD)M06-2X/6-31G(d) level of theory in tetrahydrofuran as a solvent, using Gaussian09 programme package [40]. The stationary points of reactions were localized at the potential energy surface and vibrational analysis was performed to verify the minima and transition states on the PES for all structures (See SI).

**Scheme 4:**
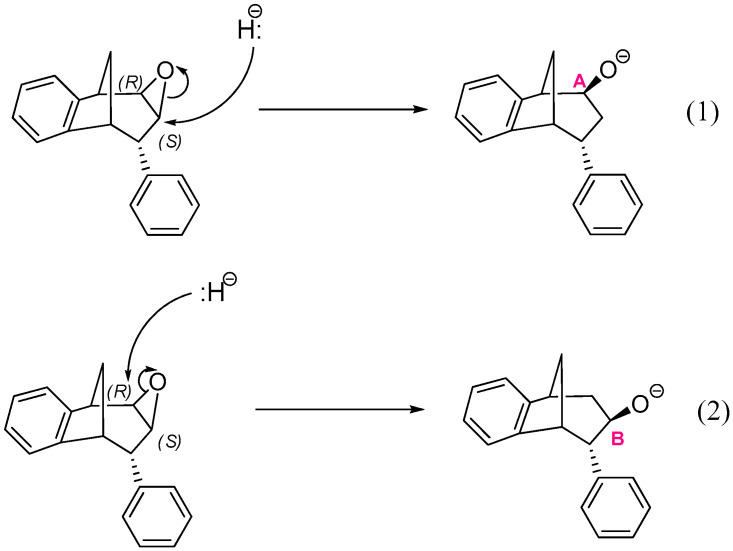
Two possible openings of epoxide, with oxygen placed above the plane of larger ring in the bridged bicyclic system.

**Scheme 5:**
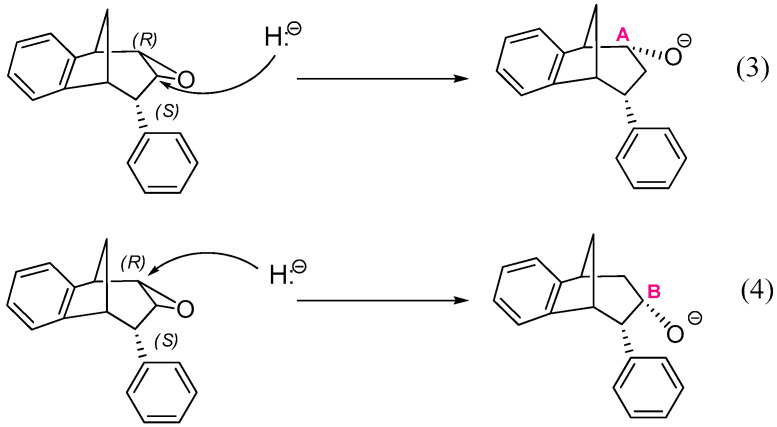
Two possible openings of epoxide, with oxygen positioned below the plane.

The epoxide opening step occurs upon the nucleophilic attack by a hydride anion at one of two carbons that belong to the epoxide ring: the middle carbon of the larger ring in the bridged bicycle system, denoted as B in Scheme 4, or the neighboring carbon atom denoted as A. A total of four reactions may occur, two starting from structures where the oxygen of epoxide is placed above the plane of the larger ring in the bridged bicycle system:

Another two reactions, starting from the structure where an oxygen atom of epoxide is placed below the plane are also possible (Scheme 5):

Reactions (1) and (3) lead to two stereoisomers of regioisomer where the hydroxyl group is placed at the C_A_ atom of the larger ring in the bridged bicycle system, while (2) and (4) give two stereoisomers of an alternative regioisomer, with an OH group at the middle carbon atom of the larger ring (i.e., at C_B_).

**Figure 6:**
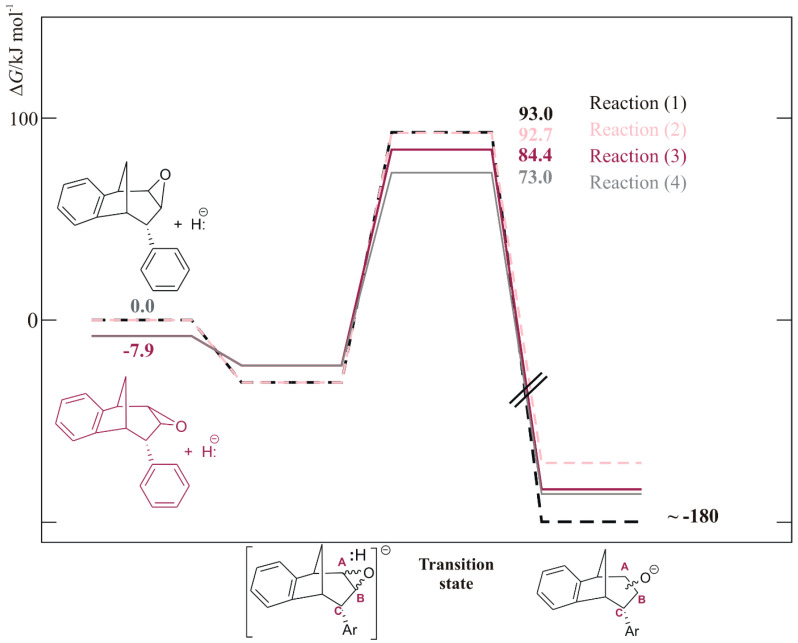
The free energy profile for reactions (1)–(4), calculated at (SMD)/M06-2X/6-31G(d) level of theory.

**Figure 7:**
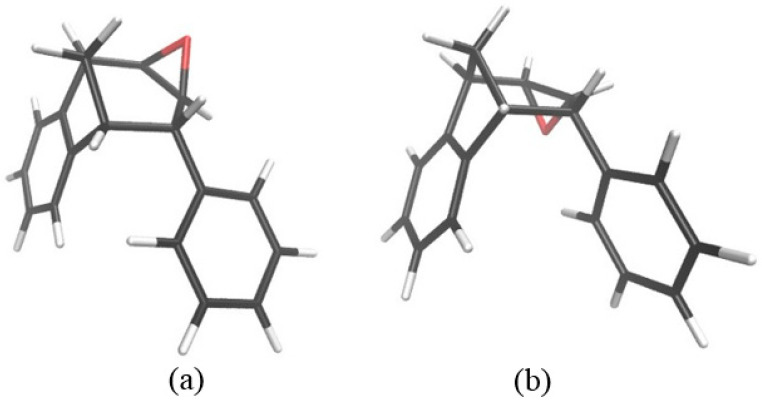
Optimized structures of two stereoisomers of starting epoxide. Isomer (**b**) is thermodynamically more stable than isomer (**a**) by –7.9 kJ mol^−1^.

The free energy profile for all four reactions (1)–(4) was calculated and is shown in Figure 6. The barrier for the ring opening was the highest and almost the same for reactions (1) and (2) (dotted lines, 93.0 and 92.7 kJ mol^−1^) where molecule *endo*-**38** has an oxygen atom of the epoxide ring above the plane (structure (a) in Figure 7). Reaction (4) had the lowest activation energy of 73.0 kJ mol^−1^, meaning the fastest process among these four reactions was the ring opening which leads to the formation of alcohol with an OH group at the C_B_ and placed below the plane.

Thermodynamically, although the sum of free energies of reactants showed that an epoxide isomer with an oxygen above the plane was less stable compared to the other isomer (for 7.9 kJ mol^−1^), this ratio was inversed when an adduct between *endo*-**38** and hydride formed: the adduct containing an isomer with an O above the plane becomes more stable (Figure 6). This makes the absolute barriers for the ring opening shown by reactions (1) and (2) even higher, being 123.9 and 124.2 kJ mol^−1^, respectively, leading to the conclusion that reactions (3) and (4), where the absolute barriers were 106.9 and 95.5 kJ mol^−1^, respectively, were favored. Due to the difference between activation energies for reactions (3) and (4) (Figure 6, full line), the product that was probably the most abundant in the mixture was a regioisomer of alcohol with an OH group at the C_B_ atom and placed below the plane.

**Figure 8:**
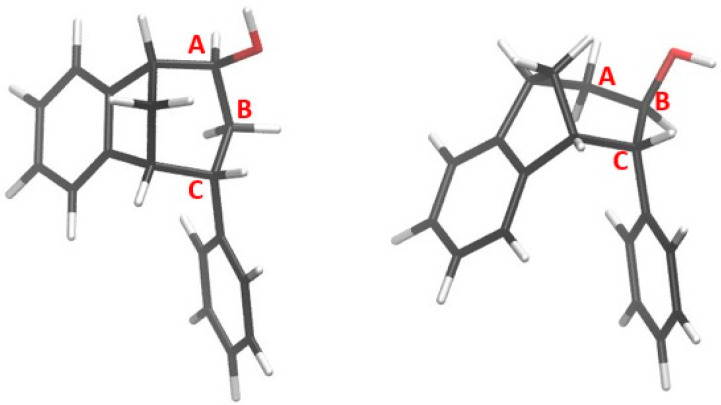
Alcohols formed from products of reactions (1) and (2).

**Figure 9:**
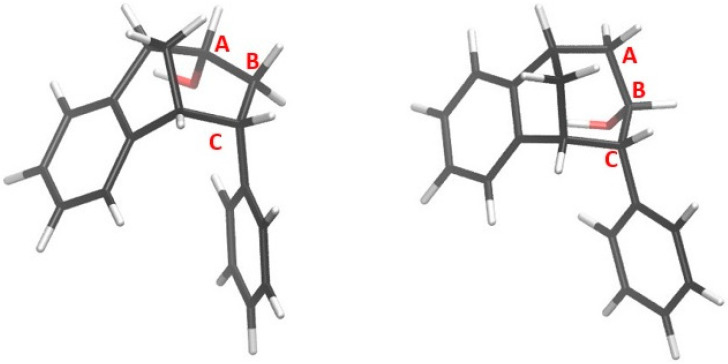
Alcohols derived from products of reactions (3) and (4).

Optimized structures of alcohols derived from products of reactions (1) and (2) are shown in Figure 8, whereas the structures of alcohols corresponding to products of reactions (3) and (4) are depicted in Figure 9.

**Figure 10:**
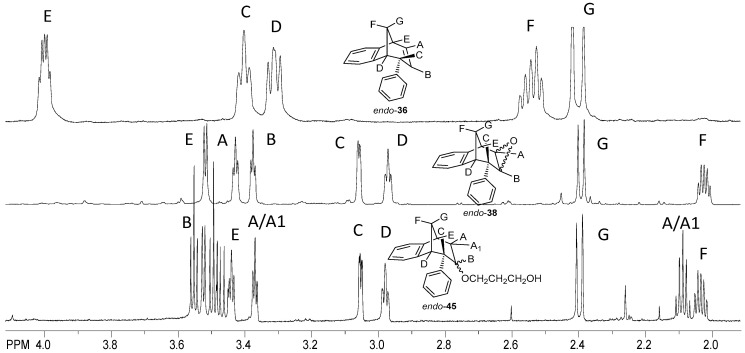
Partial ^1^H NMR spectra of starting compound *endo*-**36** and reaction products epoxyde *endo*-**38** and ether *endo*-**45**.

Figure 10 shows the aliphatic part of the ^1^H NMR spectrum of the isolated epoxy derivative endo-**38** in comparison with the corresponding starting *endo*-**36**. It can be seen that there was no signal for protons on the *sp2* carbon (between 5.00 and 6.50 ppm), which means that the double bond was broken and the epoxy ring has been formed. Instead of the signals in the mentioned region, new triplets can be seen at 3.45 and 3.39 ppm for protons A and B with coupling constants of 3.9 and 4.1 Hz, respectively.

**Figure 11:**
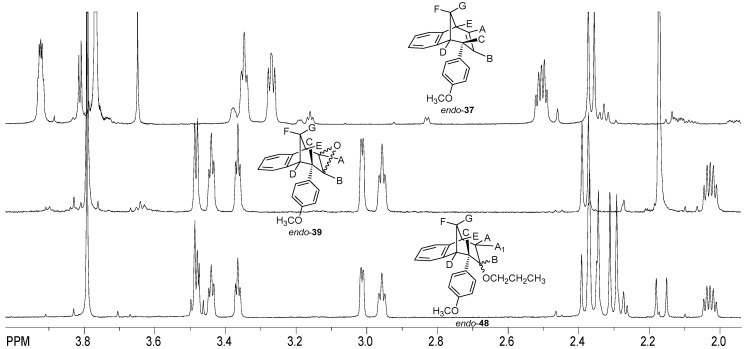
Partial ^1^H NMR spectra of starting compound *endo*-**37** and reaction products epoxyde *endo*-**39** and ether *endo*-**48**.

The signal at 3.54 ppm was a doublet for the proton E with the coupling constant of 4.8 Hz. The triplet and doublet at 3.07 and 2.99 ppm belonged to the signals of protons C and D with coupling constants of 5.0 and 4.1 Hz, respectively. Proton G showed a doublet at 2.41 ppm with a coupling constant of 10.8 Hz. The signal at chemical shift 2.05 ppm was a multiplet belonging to proton F. The difference between the signals of *endo*-**38** and the corresponding ether *endo*-**45** is the presence of an expected number of new signals in the aliphatic region for the new aliphatic chain as a substituent following the substitution of the hydroxy proton. The same tendency can be seen for the methoxy derivatives starting with photoproduct *endo*-**37**, corresponding epoxide *endo*-**39** and ether *endo*-**48** (Figure 11).

**Figure 12:**
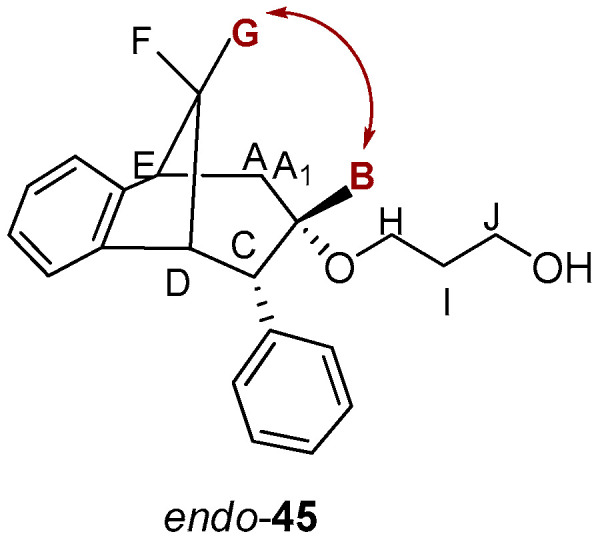

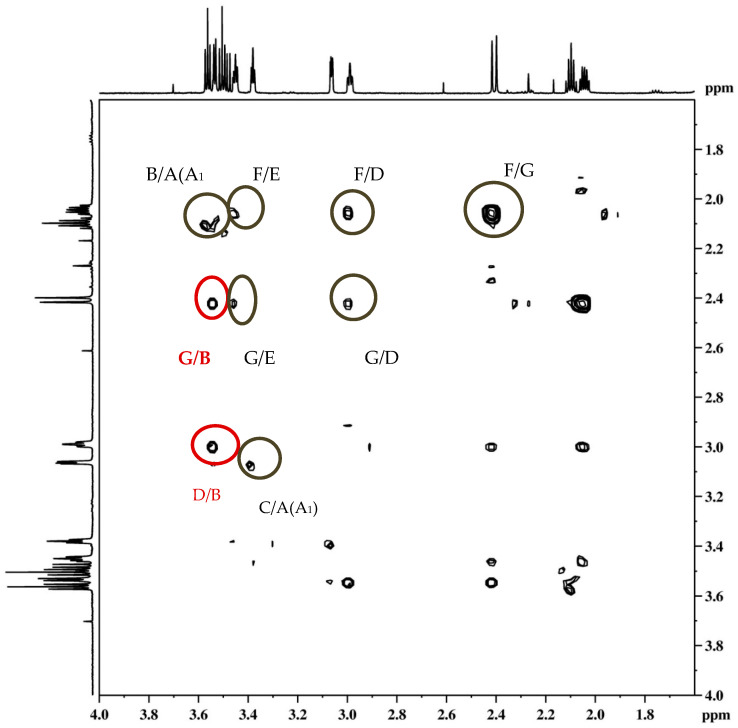
NOESY spectrum of ether *endo*-**45**.

The stereochemistry on the reactive carbon was determined for some ethers according to their NOESY spectra. From the NOESY spectrum of the ether *endo*-**45** (Figure 12) the coupling of proton F with protons E, D and G, protons A/A1 with protons C and B, and proton G with protons E and D can be observed. The most significant coupling was of proton B with protons G and D within known stereochemistry.

On the basis of the crucial interactions, it was concluded that the obtained stereoisomer was one in which the ether group was below the imaginary plane of the bicyclic skeleton. Accordingly, proton B was located above the plane of the bicyclic skeleton. These conclusions were also confirmed by calculations suggesting at the same time the stereochemistry of the corresponding alcohol *endo*-**40** and epoxide *endo*-**38** for the synthesis of the final ethers according to Scheme 3.

### 2.4. Friedel-Crafts Acylation of the Furo-Benzobicyclo[3.2.1]octadiene Photoproduct

**Scheme 6:**
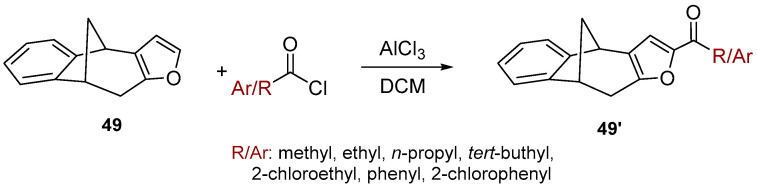
Acylation of the furo-benzobicyclo[3.2.1]octadiene photoproduct **49**.

**Figure 13:**
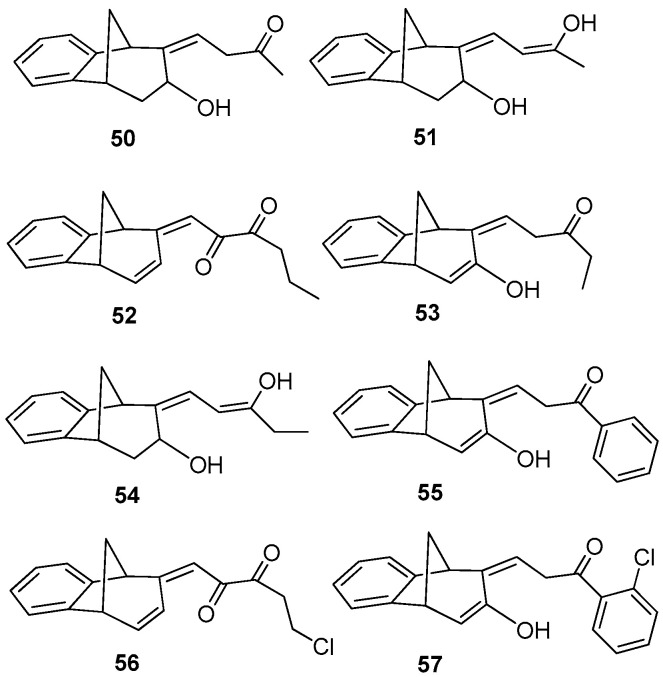
Isolated products of acylation reaction.

Benzobicyclo[3.2.1]octadiene photoproduct **49** [41] with the annulated furan ring was the starting substrate (Scheme 6) for syntheses of the fourth group of compounds (Figure 2D). The Friedel–Crafts acylation reaction of **49** was performed with various carbonyl chlorides in the seal tube. During the reaction under applied conditions, in all cases the furan ring of **49** opened to give novel mono- or dihydroxy and mono- or dicarbonyl derivatives **50**–**57** (7–59%) with the characteristic benzobicyclo[3.2.1]-skeleton (Figure 13).

**Figure 14:**
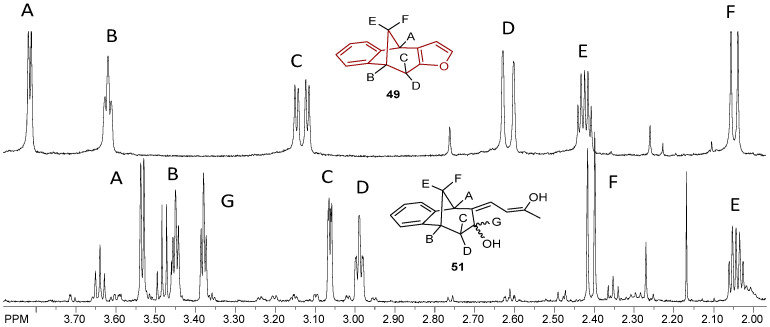
Partial ^1^H NMR spectra of starting compound **49** and the acylation product **51** with the opened furan ring.

The structure and purity of the synthesized products of acylation were confirmed by NMR techniques as well as High Resolution Mass Spectrometry (HRMS) analyses (see experimental and Figure 14). In comparison with the aliphatic part of the proton NMR spectrum of **49** and characteristic six signals, seven signals were present in the same region for compound **53**. Together with the absence of the singlet for the furyl proton but two new doublets with allylic coupling constants, the opening of the heterocyclic ring as well as the structure were confirmed. However, as in the cases of several derivatives, the formed tautomers were in an equilibrium, and the products were isolated at lower amounts. Nevertheless, only two compounds from this group were tested as cholinesterase inhibitors.

Acylated furo-benzobicyclo[3.2.1]octadiene **51** exhibited the maximum of anti-AChE activity of all of the tested compounds (Table 4). It seems that a smaller saturated molecule with two effective nucleophile groups can contribute to interactions with the AChE active site. Even though compound **51** was not a potent inhibitor of BChE, its structure is similar to opened oxime ether **31** (Figure 4), a potent inhibitor of BChE. However, none of tested compounds inhibited the enzymes in nanomolar range like etopropazine, a drug for Parkinson’s disease [42].

## 3. Materials and Methods

### 3.1. General

Petroleum ether (PE)(VWR Prolabo Chemicals, Fontenay-sous-Bois, France), bp 40–60 °C, was used. Solvents were purified by distillation. Column chromatography was carried out on columns with silica gel (Fluka 0.063–0.2 nm and Fluka 60 Å, technical grade, Merck, New York, NY, USA). Thin layer chromatography (TLC) was carried out using plates coated with silica gel (0.2 mm, 0.5 mm, 1.0 mm, Kiselgel 60 F_254_, Merck, New York, NY, USA). Organic layers were routinely dried with anhydrous MgSO_4_ and evaporated using a rotary evaporator (Heidolph, Schwabach, Germany). ^1^H and ^13^C NMR spectra were recorded on a spectrometer (Bruker AV-600 Spectrometer, Billerica, MA, USA) at 600 MHz. All NMR spectra were measured in CDCl_3_ using tetramethylsilane (Merck, New York, NY, USA) as reference. The following abbreviations were used: Sh—shoulder (in UV spectra), s—singlet, d—doublet, t—triplet, q—quartet, dd—doublet of doublets, m—multiplet, br—broad, PE—petroleum ether, E—diethyl ether. UV spectra were measured on a UV/VIS spectrophotometer (Varian Cary 50 UV/VIS Spectrophotometer, Palo Alto, CA, USA). Mass spectra were obtained on a UPLC-MS system (Acquity UPLC coupled with SQD mass spectrometer, Milford, MA, USA). Melting points were obtained using a microscope equipped apparatus (Original Kofler Mikroheitztisch apparatus Reichert, Vienna, Austria) and have not been corrected. HRMS analyses were carried out on a mass spectrometer (MALDI TOF/TOF analyzer (Merck, New York, NY, USA), equipped with Nd:YAG laser (Merck, New York, NY, USA) operating at 355 nm with a firing rate of 200 Hz in the positive (H^+^) or negative (-H) ion reflector mode. Compounds *endo*-**1**, *endo*-**36**, *endo*-**37** and **49** were prepared in our laboratory by photochemical strategy and published before [31,41,43,44].

### 3.2. Photochemistry

#### Photochemical Synthesis of *endo*-**1**, *endo*-**36** and *endo*-**37**

The solutions of a mixture of isomers of corresponding Wittig reaction products were purged with argon for 20 min and irradiated at 350 nm in petroleum ether (5 × 10^−3^ M) in a Rayonet reactor in a Pyrex tube for 4 h. The solvent was removed in vacuum and the oily residue chromatographed on silica gel column using petroleum ether as eluent.


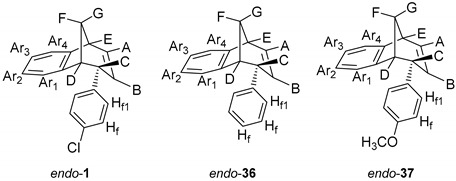


After column chromatography, 77% of *endo*-**1**, 90% of *endo*-**36** and 70% of *endo*-**37** was isolated. High-molecular-weight products remained on the column.

*11-(4-Chlorophenyl)tricyclo[6.3.1.0^²,7^]dodeca-2,4,6,9-tetraene* (*endo*-**1**) [39]: colorless oil; *R_f_* 0.66 (petroleum ether/dichloromethane = 9:1); UV (96% EtOH) *λ*_max_ (log *ε*) 276 (3.19), 268 (3.22), 224 (4.20, sh); ^1^H NMR (CDCl_3_, 600 MHz) δ/ppm 7.14 (d, *J* = 8.4 Hz, 2H, H_ar2_), 7.12 (d, *J* = 7.3 Hz, 1H), 7.03 (td, *J* = 7.4; 1.0 Hz, 1H), 6.84 (td, *J* = 7.4; 1.0 Hz, 1H), 6.65 (d, *J* = 8.3 Hz, 2H, H_ar1_), 6.40–6.35 (m, 1H, H_A_), 6.23 (d, *J* = 7.3 Hz, 1H, H_ar_), 5.25 (ddd, *J* = 9.6; 4.0; 1.9 Hz, 1H, H_B_), 3.96–3.92 (m, 1H), 3.34 (t, *J* = 9.4; 4.7 Hz, 1H), 3.29 (dd, *J* = 6.1; 4.7 Hz, 1H), 2.54–2.50 (m, 1H, H_F_), 2.37 (d, *J* = 10.0 Hz, 1H, H_G_); ^13^C NMR (CDCl_3_, 150 MHz) δ/ppm 140.6 (s), 137.3 (s), 132.9 (s), 130.9 (s), 134.7 (d), 129.2 (2d), 127.3 (d), 125.7 (d), 125.6 (d), 125.4 (2d), 124.7 (d), 119.7 (d), 48.0 (d), 45.3 (d), 43.7 (t), 39.9 (d); HRMS (*m/z*) for C_18_H_15_Cl: [M + H]^+^_calcd_ = 265.0789, [M + H]^+^_measured_ = 265.0786.

*endo-6-Phenyl-6,9-dihydro-5H-5,9-methano-benzocycloheptene* (*endo-***36**) [38]: colorless crystals; mp 51 °C; *R*_f_ 0.44(petroleum ether); UV (EtOH) *λ*_max_ (log*ε*) 275 (2.88), 263 (2.90), 203 (4.46); ^1^H NMR (CDCl_3_, 600 MHz) *δ* 7.17–7.18 (m, 3H, H_f_), 7.12 (d, *J* = 7.3 Hz, 1H, H_Ar4_), 7.03 (t, *J* = 7.4 Hz, 1H, H_Ar3_), 6.81 (t, *J* = 7.4 Hz, 1H, H_Ar2_), 6.73–6.76 (m, 2H, H_f1_), 6.37 (ddd, *J*_AB_ = 9.5 Hz; *J*_AE_ = 6.0 Hz; *J*_AC_ = 2.5 Hz, 1H, H_A_), 6.18 (d, *J* = 7.3 Hz, 1H, H_Ar1_), 5.33 (dt, *J*_AB_ = 9.5 Hz; *J*_BC_ = 2.5 Hz, 1H, H_B_), 3.98 (m, 1H, H_C_), 3.38 (t, *J*_CD_ = *J*_DF_ = 4.7 Hz, 1H, H_D_), 3.29 (dd, *J*_AE_ = 6.0 Hz; *J*_EF_ = 4.7 Hz, 1H, H_E_), 2.52 (dt, *J*_FG_ = 9.9 Hz; *J*_EF_ = *J*_DF_ = 4.7 Hz, 1H, H_F_), 2.38 (d, *J*_FG_ = 9.9 Hz, 1H, H_G_); ^13^C NMR (CDCl_3_, 150 MHz) *δ* 152.27 (s), 142.44 (s), 141.93 (s), 132.71 (d), 128.21 (d), 127.60 (d), 126.34 (d), 126.10 (d), 126.05 (d), 125.97 (d), 124.89 (d), 120.03 (d), 48.59 (d), 46.29 (d), 44.13 (t, C_FG_), 40.37 (d); MS *m/z* (EI) 232 (M^+^, 100%), 117 (25), 115 (10); HRMS (*m/z*) for C_18_H_16_: [M + H]^+^_calcd_ = 232.1634, [M + H]^+^_measured_ = 232.1655.

*11-(4-Methoxyphenyl)tricyclo[6.3.1.0^²,7^]dodeca-2,4,6,9-tetraene* (*endo*-**37**) [40]: colorless crystals; mp 45–47 °C; *R*_f_ (petroleum ether/dichloromethane = 8:2) = 0.27; ^1^H NMR (CDCl_3_, 600 MHz) δ/ppm: 7.11 (d, 1H, *J* = 7.3 Hz), 7.03 (dt, 1H, *J* = 7.3; 1.0 Hz), 6.82 (dt, 1H, *J* = 7.3; 1.0 Hz), 6.72 (d, 2H, *J* = 8.6 Hz), 6.64 (d, 2H, *J* = 8.6 Hz), 6.32-6.36 (m, 1H), 6.25 (d, 1H, *J* = 7.3 Hz), 5.28 (dt, 1H, *J* = 9.6; 2.6 Hz), 3.91-3.94 (m, 1H), 3.77 (s, 3H), 3.35 (t, 1H, *J* = 4.5 Hz), 3.27 (dd, 1H, *J* = 6.3; 4.7 Hz), 2.49-2.53 (m, 1H), 2.37 (d, 1H, *J* = 9.9 Hz); ^13^C NMR (CDCl_3_, 75 MHz) δ/ppm: 157.6 (s), 152.0 (s), 141.7 (s), 134.2 (s), 134.1 (d), 128.8 (2d), 126.3 (d), 125.8 (d), 125.5 (d), 124.5 (d), 119.6 (d), 112.6 (2d), 52.4 (q), 48.2 (d), 45.0 (d), 43.7 (t), 40.3 (d); MS *m/z* (%, fragment): 262 (100, M^+^), 154 (75), 115 (50); HRMS (*m/z*) for C_19_H_18_O: [M + H]^+^_calcd_ = 262.1352, [M + H]^+^_measured_ = 262.1351.

### 3.3. Synthesis of Benzylamine Derivatives

#### Amination Reactions of *endo*-**1** with Primary Benzylic Amines

To a solution of BrettPhos (0.1 eq) and Pd(OAc)_2_ (0.05 eq) in dioxane, 4% of water was added. After the addition of water, the solution was heated to 120 °C for 2 min. The reaction mixture changed color from light yellow, over dark red to dark green. Compound *endo*-1, different amine (2 eq) and KOtBu (1.4 eq) were added into a sealed tube. The reaction mixture was stirred for 20 h on 180 °C. After the removal of the solvent, the crude product residue was purified by repeated column chromatography using petroleum ether/dichlorometane mixture as an eluent. All of the obtained compounds *endo*-**4**–**20** were isolated in the first fractions as yellow oils, while BrettPhos remained on the chromatographic column. In all cases, the conversion of the amination reaction was complete. The spectroscopic data for some of the previously synthesized amines (*endo*-**4**, *endo*-**6**, *endo*-**7** and *endo*-**8**) are given below.


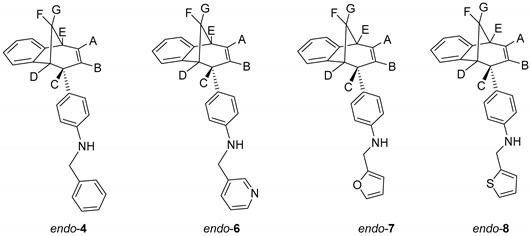


*N-Benzyl-4-((5R,6S,9S)-6,9-dihydro-5H-5,9-methanobenzo[7]annulen-6-yl)aniline* (*endo*-**4**): Column chromatography on silica gel using petroleum ether/dichloromethane (0%–60%) as eluent afforded 0.080 g (48.3%) of *endo*-**4**: *R*_f_ (PE / DCM = 5:1) = 0.43; UV (EtOH) *λ*_max_/nm (*ε*/dm^3^mol^−1^cm^−1^): 251 (11,312), 306 (Sh, 2527); IR *ν*_max_/cm^−1^ (diamond ATR): 3408.78, 1613.45, 1517.61, 1468.98; ^1^H NMR (CDCl_3_, 600 MHz) *δ*/ppm: 7.38–7.25 (m, 5H), 7.10 (d, 1H, *J* = 7.4 Hz), 7.02 (dt, 1H, *J* = 7.4; 1.1 Hz), 6.83 (dt, 1H, *J* = 7.4; 1.1 Hz), 6.54 (d, 2H, *J* = 8.6 Hz), 6.47 (d, 2H, *J* = 8.6 Hz), 6.32 (d, 2H, *J* = 7.4 Hz, H_ar_, H_A_), 5.27 (td, 1H, *J* = 9.5; 1.9 Hz, H_B_), 4.28 (s, 2H), 3.92–3.85 (m, 2H, H_C_, NH), 3.35 (t, 1H, *J* = 4.2 Hz, H_D_), 3.27 (dd, 1H, *J* = 6.3; 4.2 Hz, H_E_), 2.55–2.47 (m, 1H, H_F_), 2.34 (d, 1H, *J* = 10.1 Hz, H_G_); ^13^C NMR (CDCl_3_, 150 MHz) *δ*/ppm: 152.1 (s), 146.1 (s), 141.9 (s), 139.1 (s), 133.8 (d), 130.6 (s), 128.6 (2d), 128.1 (d), 128.1 (d), 127.1 (d), 126.7 (2d), 126.0 (d), 125.9 (d), 125.4 (d), 124.4 (d), 119.5 (d), 111.9 (2d), 48.4 (d), 48.2 (t), 45.2 (d), 43.7 (t), 40.0 (d); MS *m/z* (%, fragment): 337 (100, M^+^); HRMS (*m/z*) for C_25_H_23_N: [M + H]^+^_calcd_ = 338.1822, [M + H]^+^_measured_ = 338.1902.

*4-((5R,6S,9S)-6,9-Dihydro-5H-5,9-methanobenzo[7]annulen-6-yl)-N-(pyridin-3-ylmethyl)-aniline* (*endo*-**6**): Column chromatography on silica gel using dichloromethane/ethanol (variable ratio) as eluent afforded 0.062 g (21.2%) of *endo*-**6** in the first fractions: *R*_f_ (DCM) = 0.1; UV (EtOH) *λ*_max_/nm (ε/dm^3^mol^−1^cm^−1^): 254 (15991); IR *ν*_max_/cm^−1^ (diamond ATR): 3370.11, 1609.55, 1531.76, 1444.89; ^1^H NMR (CDCl_3_, 600 MHz) δ/ppm: 8.62 (s, 1H), 8.52 (dd, 1H, *J* = 5.4; 1.3 Hz), 7.67 (d, 1H, *J* = 7.4 Hz), 7.30–7.23 (m, 3H), 7.13 (d, 1H, *J* = 7.4 Hz), 7.09 (dt, 1H, *J* = 7.7; 1.2 Hz), 7.03 (dt, 1H, *J* = 7.7; 1.2 Hz), 6.85 (d, 1H, *J* = 8.5 Hz), 6.83 (d, 2H, *J* = 8.5 Hz), 6.60-6.58 (m, 2H, H_ar_, H_A_), 5.41 (t, 1H, *J* = 3.7 Hz, H_B_), 4.36 (s, 2H), 3.79–3.76 (m, 2H, H_C_, NH), 3.32 (t, 1H, *J* = 5.1 Hz, H_D_), 2.69 (td, 1H, *J* = 8.3; 1.2 Hz, H_E_), (signal for H_F_ and H_G_ can’t be assigned because of the presence of the catalyst BrettPhos); MS *m*/*z* (%, fragment): 338 (100, M^+^).

Column chromatography on silica gel using dichloromethane/ethanol (variable ratio) as eluent afforded 0.051 g (43.8%) of *endo*-**7** and 0.064 g (51.5%) of *endo***-8**.

*4-((5R,6S,9S)-6,9-Dihydro-5H-5,9-methanobenzo[7]annulen-6-yl)-N-(fur-2-ylmethyl)aniline* (*endo*-**7**): *R*_f_ (PE / DCM = 5:1) = 0.62; UV (EtOH) *λ*_max_/nm (*ε*/dm^3^mol^−1^cm^−1^): 255 (28024), 358 (12474); IR *ν*_max_/cm^−1^ (diamond ATR): 3412.68, 1603.44, 1517.61, 1468.98; ^1^H NMR (CDCl_3_, 600 MHz) δ/ppm: 7.34 (d, 1H, *J* = 1.6 Hz, H_5f_), 7.09 (d, 1H, *J* = 7.2 Hz), 7.00 (t, 1H, *J* = 7.4 Hz), 6.80 (t, 1H, *J* = 7.2 Hz), 6.53 (d, 2H, *J* = 8.5 Hz), 6.49 (d, 2H, *J* = 8.5 Hz), 6.33–6.24 (m, 3H, H_ar_, H_A_), 6.20 (d, 1H, *J*= 3.2 Hz, H_3f_), 5.26 (d, 1H, *J* = 9.4 Hz, H_B_), 4.26 (s, 2H), 3.88–3.83 (m, 1H, H_C_), 3.31 (t, 1H, *J* = 4.8 Hz, H_D_), 3.24 (t, 1H, *J* = 4.8 Hz, H_E_), 2.51–2.43 (m, 1H, H_F_), 2.33 (d, 1H, *J* = 9.7 Hz, H_G_); ^13^C NMR (CDCl_3_, 150 MHz) δ/ppm: 152.9 (s), 152.5 (s), 146.0 (s), 142.3 (s), 141.8 (d), 134.3 (d), 132.2 (s), 129.1 (2d), 121.1 (d), 126.4 (d), 125.9 (d), 124.9 (d), 119.9 (d), 112.8 (2d), 110.2 (d), 106.9 (d), 48.8 (d), 45.7 (d), 44.1 (t), 40.5 (d); MS *m*/*z* (%, fragment): 327 (100, M^+^); HRMS (*m/z*) for C_23_H_21_NO: [M + H]^+^_calcd_ = 328.1609, [M + H]^+^_measured_ = 328.1689.

*4-((5R,6S,9S)-6,9-dihydro-5H-5,9-methanobenzo[7]annulen-6-yl)-N-(thien-2-ylmethyl)aniline* (*endo*-**8**): *R*_f_ (PE / DCM = 5:1) = 0.70; UV (EtOH) *λ*_max_/nm (*ε*/dm^3^mol^−1^cm^−1^): 247 (18373); IR *ν*_max_/cm^−1^ (diamond ATR): 3413.17, 1613.45, 1516.18, 1468.98; ^1^H NMR (CDCl_3_, 600 MHz) δ/ppm: 7.48 (d, 1H, *J* = 4.9 Hz) 7.38 (d, 1H, *J* = 7.2 Hz), 7.30 (t, 1H, *J* = 7.1 Hz), 7.27 (broad s), 7.25–7.22(m, 2H), 7.11 (t, 1H, *J* = 7.2 Hz), 6.83 (d, 2H, *J* = 8.6 Hz), 6.78 (d, 2H, *J* = 8.6 Hz), 6.62-6.56 (m, 2H, H_ar_, H_A_), 5.55 (d, 1H, *J* = 9.5 Hz, H_B_), 4.75 (s, 2H), 4.20–4.13 (m, 2H, H_C_, NH), 3.61 (t, 1H, *J* = 4.7 Hz, H_D_), 3.53 (t, 1H, *J* = 4.7 Hz, H_E_), 2.79–2.74 (m, 1H, H_F_), 2.62 (d, 1H, *J* = 9.7 Hz, H_G_);^13^C NMR (CDCl_3_, 150 MHz) *δ*/ppm: 152.5 (s), 145.9 (s), 143.1 (s), 142.3 (s), 134.4 (d), 132.3 (s), 129.1 (2d), 127.1 (d), 126.7 (d), 126.4 (d), 125.8 (d),125.0 (d), 124.9(d), 124.5 (d), 119.9 (d), 112.7 (2d), 48.8 (d), 45.7 (d), 44.1 (t), 43.7 (t), 40.4 (d); MS *m*/*z* (%, fragment): 343 (100, M^+^); HRMS (*m*/*z*) for C_23_H_21_NS: [M + H]^+^_calcd_ = 344.1394, [M + H]^+^_measured_ = 344.1474.

### 3.4. Synthesis of Oxime Derivatives

#### 3.4.1. Synthesis of Oxime

A mixture of aldehyde **21** (0.223 mmol), hydroxylamine hydrochloride (0.112 mmol, 0.5 eq), and pyridine (0.05 mL) was refluxed in ethanol (1 mL) for 1 h on a water bath. Completion of the reaction was checked by TLC (DCM/PE, 1:3). At the end of the reaction, ethanol was evaporated to dryness. Crude material was purified on column chromatography using different polarity of solvents. The product was additionally purified using preparative TLC (DCM/PE).

#### 3.4.2. Synthesis of Oxime Ethers

A solution of oxime **22** (0.251 mmol) and alkyl bromide (0.276 mmol, 1.1 eq) in dry acetone (2 mL) was refluxed in the presence of dry potassium carbonate (0.376 mmol, 1.5 eq) for 48 h on a water bath. Completion of the reaction was checked by TLC (DCM/PE). At the end of the reaction, acetone was evaporated to dryness. Crude material was purified on column chromatography using different polarity of solvents (DCM/PE) and the product was also additionally purified using preparative TLC.


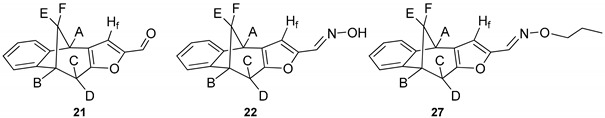


*(4R,9R)-9,10-Dihydro-4H-4,9-methanobenzo[4,5]cyclohepta[1,2-b]furan-2-carbaldehyde* (**21**) [33]: 180 mg (50.0%); *R*_f_ (petroleum ether/dichloromethane = 20:1) = 0.23; UV (EtOH) *λ*_max_ (ε/dm^3^ mol^−1^ cm^−1^): 308 (16794) nm;IR *ν*_max_/cm^−1^ (NaCl): 1666, 1504; ^1^H NMR (CDCl_3_, 300 MHz) δ/ppm: 9.41 (s, 1H), 7.32 (dd, 1H, *J* = 7.2; 1.4 Hz), 7.16–7.08 (m, 4H, 3H), 3.91 (d, 1H, *J* = 4,4 Hz, H_A_), 3.66 (dt, 1H, *J* = 4.9; 0.5 Hz, H_B_), 3.20 (dd, 1H, *J* = 17.8; 4.9 Hz, H_C_), 2.72 (dd, 1H, *J* = 17.8; 0.5 Hz, H_D_), 2.48 (ddd, 1H, *J* = 10.7; 4.9; 4.4 Hz, H_E_), 2.4 (d, 1H, *J* = 10.7 Hz, H_F_); ^13^C NMR (CDCl_3_, 75 MHz) δ /ppm: 176.6 (d), 156.0 (s), 151.3 (s), 150.6 (s), 144.1 (s), 128.5 (s), 126.9 (d), 126.9 (d), 124.1 (d), 121.0 (2d), 42.5 (t), 39.6 (d), 39.1 (d), 31.4 (t); MS *m*/*z* (%, fragment): 224 (100, M^+^), 167 (75), 115 (50); HRMS (*m/z*) for C_19_H_18_O: [M + H]^+^_calcd_ = 224.3355, [M + H]^+^_measured_ = 224.3347.

*(E)-9,10-Dihydro-4H-4,9-methanobenzo[4,5]cyclohepta[1,2-b]furan-2-carbaldehyde oxime* (**22**): 16.3 mg (40%); *R*_f_ (PE / DCM = 1:1) = 0.33; IR *ν*_max_/cm^−1^ (NaCl): 3400, 2943, 1668, 1613, 1572, 1503, 1460; ^1^H NMR (CDCl_3_, 600 MHz) δ/ppm: 7.36 (d, 1H, *J* = 6.6 Hz), 7.18-7.05 (m, 4H), 6.06 (s, 1H), 4.62 (d, 1H, *J* = 2.5 Hz), 3.81 (d, 1H, *J* = 4.0 Hz), 3.59-3.54 (m, 2H), 2.40-2.37 (m, 2H); ^13^C NMR (CDCl_3_, 150 MHz) δ/ppm: 152.1 (s), 146.6 (s), 141.9 (s), 135.9 (s), 131.6 (s), 126.9 (d), 126.4 (d), 125.1 (d), 121.5 (d), 104.6 (2d), 67.4 (t), 48.9 (d), 39.7 (t), 39.6 (d); MS *m*/*z* (%, fragment) (EI): 239 (100); HRMS (*m*/*z*) for C_19_H_19_ON: [M + H]^+^_calcd_ = 240.0946; [M + H]^+^_measured_ = 240.0941.

*(Z)-9,10-Dihydro-4H-4,9-methanobenzo[4,5]cyclohepta[1,2-b]furan-2-carbaldehyde O-propyl oxime* (**27**): 13.6 mg (32%); *R*_f_ (PE / DCM = 1:5) = 0.55; IR *ν*_max_/cm^−1^ (NaCl): 3434, 2918, 1731, 1638, 1234; ^1^H NMR (CDCl_3_, 600 MHz) δ/ppm: 7.32 (d, 1H, *J* = 7.4 Hz), 7.19–7.07 (m, 4H), 6.94 (s, 1H), 3.86 (d, 1H, *J* = 4.5 Hz), 3.68 (d, 1H, *J* = 5.0 Hz), 3.48 (q, 2H, *J* = 7.0 Hz), 3.15 (dd, 1H, *J* = 17.4; 5.0 Hz), 2.67 (dd, 1H, *J* = 17.4; 1.3 Hz), 2.51–2.47 (m, 1H), 2.03 (d, 1H, *J* = 11.2 Hz), 1.34–1.29 (m, 2H), 0.88 (t, 3H, *J* = 6.9 Hz); ^13^C NMR (CDCl_3_, 150 MHz) δ/ppm: 152.0 (s),150.5 (s), 143.8 (s), 135.6 (s), 128.1 (s), 126.9 (d), 126.9 (d), 124.1 (d), 124.1 (d), 120.2 (d), 67.0 (t), 42.5 (t), 39.6 (d), 38.9 (d), 31.2 (t), 29.6 (t), 22.7 (q); MS *m*/*z* (%, fragment) (EI): 281 (100); HRMS (*m*/*z*): [M + H]^+^_calcd_ = 282.1416; [M + H]^+^_measured_ = 282.1419.

### 3.5. Synthesis of Epoxide Derivatives

#### Synthesis of (2*R*,7*R*,8*S*,8a*S*)-8-Phenyl-1a,7,8,8a-tetrahydro-2*H*-2,7-methanobenzo[4,5]cyclohepta[1,2-*b*]oxirene (*endo*-**38**) and (2*R*,7*R*,8*S*,8a*S*)-8-(4-Methoxyphenyl)-1a,7,8,8a-tetrahydro-2*H*-2,7-methanobenzo[4,5]cyclohepta[1,2-*b*]oxirene (*endo*-**39**)

Epoxide derivatives *endo*-**38** and *endo*-**39** were prepared by epoxidation reaction using *meta*-chlorperbenzoic acid (*m*-CPBA). Photoproducts *endo*-**36** and *endo*-**37** were dissolved in 20 mL dry dioxane, and *m*-CPBA was added. The reaction mixture was stirred at room temperature overnight. After the reaction was complete, the saturated water solution of NaHCO_3_ was added. The organic layer was washed twice with water and dried over MgSO_4_, filtered and evaporated to obtain crude material. The crude material was purified using column chromatography.


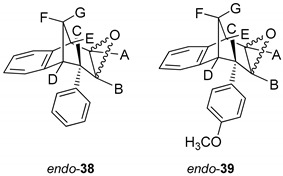


*(2R,7R,8S)-8-Phenyl-1a,7,8,8a-tetrahydro-2H-2,7-methanobenzo[4,5]cyclohepta[1,2-b]-oxirene* (*endo-***38**): Yield 61%; *R*_f_ (petroleum ether/dichloromethane): 0.27; ^1^H NMR (cdcl_3_, 600 mhz) δ/ppm: 7.53 (d, 2H, *J* = 8.8 Hz, Ar), 7.30 (d, 1H, *J =* 7.4 Hz, Ar), 7.16 (t, 2H, *J =* 7.3 Hz, Ar), 6.93 (t, 1H, *J* = 7.3 Hz, Ar), 6.70 (dd, 2H, *J* = 8.8; 7.3 Hz, Ar), 6.26 (d, 1H, *J* = 7.4 Hz, Ar), 3.54 (d, 1H, *J* = 4.8 Hz, H_E_), 3.45 (t, 1H, *J* = 4.1 Hz, H_A_), 3.39 (t, 1H, *J* = 3.9 Hz, H_B_), 3.07 (d, 1H, *J* = 4.1 Hz, H_C_), 2.99 (t, 1H, *J* = 5.0 Hz, H_D_), 2.41 (d, 1H, *J* = 10.8 Hz, H_G_), 2.0–2.02 (m, 1H, H_F_);^13^C NMR (cdcl_3_, 75 mhz) δ/ppm: 144.6 (s), 143.2 (s), 141.2 (s), 133.2 (d), 129.3 (d), 128.2 (2d), 127.3 (2d), 126.4 (d), 125.3 (d), 122.1 (d), 54.1 (d), 52.5 (d), 45.8 (d), 43.2 (t), 40.1 (d), 34.9 (t); MS *m*/*z* (%, fragment) (EI): 248; HRMS (*m*/*z*) for C_18_H_16_O: [M + H]^+^_calcd_ = 249.1201, [M + H]^+^_measured_ = 249.1190.

*(2R,7R,8S,8as)-8-(4-Methoxyphenyl)-1a,7,8,8a-tetrahydro-2H-2,7-methanobenzo[4,5]cyclohepta[1,2-b]oxirene* (*endo***-39**): Yield 50%; *R*_f_ (petroleum ether): 0.13; ^1^H NMR (CDCl_3_, 600 MHz) δ/ppm: 7.29 (d, 1H, *J* = 7.3 Hz, Ar), 7.16 (t, 1H, *J* = 7.2 Hz, Ar), 6.95 (t, 1H, *J* = 7.2 Hz, Ar), 6.75 (d, 2H, *J* = 8.3 Hz, Ar), 6.61 (d, 2H, *J* = 8.3 Hz, Ar), 6.32 (d, 1H, *J* = 7.3 Hz, Ar), 3.79 (s, 3H, OCH_3_), 3.48 (d, 1H, *J* = 5.0 Hz, H_E_), 3.44 (t, 1H, *J* = 4.0 Hz, H_A_), 3.36 (t, 1H, *J* = 3.8 Hz, H_B_), 3.00 (d, 1H, *J* = 4.0 Hz, H_C_), 2.95 (t, 1H, *J* = 5.0 Hz, H_D_), 2.38 (d, 1H, *J* = 10.8 Hz, H_G_), 2.05–2.00 (m, 1H, H_F_); ^13^C NMR (CDCl_3_, 150 MHz) δ/ppm: 152.5 (s), 142.9 (s), 135.9 (s), 133.8 (s), 129.6 (2d), 126.8 (d), 126.7 (d), 125.8 (d), 122.5 (d), 113.8 (2d), 55.2 (d), 54.2 (d), 53.2 (q), 44.8 (d), 42.9 (t), 40.6 (d), 35.3 (d);MS *m*/*z* (%, fragment) (EI): 278; HRMS (*m*/*z*) for C_19_H_16_O_2_: [M + H]^+^_calcd_ = 279.1306, [M + H]^+^_measured_ = 279.1325.

### 3.6. Epoxide Ring Opening

To a solution of starting epoxide (0.8g) in THF, lithium aluminum hydride (2.5 eq) was added. The reaction mixture was stirred at 76 °C during 24 h. After the reaction was complete, NaHCO_3_ was added and pH was adjusted to 7. The water layer was washed twice with diethyl ether. Organic layers were dried over MgSO_4_, filtered and evaporated to obtain crude material. Crude material was purified on column chromatography using petroleum ether/dichloromethane as eluent.


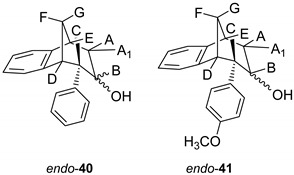


*(6S)-6-Phenyl-6,7,8,9-tetrahydro-5H-5,9-methanobenzo[7]anulen-7-ol* (*endo-***40**): Yield 40%; *R*_f_ (petroleum ether/ dichloromethane 40%): 0.20; ^1^H NMR (CDCl_3_, 600 MHz) δ/ppm 8.00–7.30 (m, 4H, Ar), 6.93–6.20 (m, 5H, Ar), 4.13 (m, 1H, H_B_) 3.40 (dd, 1H, *J* = 12.7, 4.7 Hz, H_A/A1/E_) 3.20 (t, 1H, *J* = 4.5 Hz, H_A/A1/E_), 3.14 (d, 1H, *J* = 4.5 Hz, H_A/A1/E_), 2.56 (d, 1H, *J* = 10.8 Hz, H_G_), 2.35 (s, 1H, OH), 2.25–2.20 (m, 1H, H_F_) 1.63 (d, 1H, *J* = 4.8 Hz, H_C/D_), 1.60 (d, 1H, *J* = 4.8 Hz, H_C/D_); ^13^C NMR (CDCl_3_, 150 MHz) δ/ppm: 143.8 (s), 142.3 (s), 133.9 (s), 129.3 (d), 128.9 (d), 127.6 (d), 127.2 (2d), 126.5 (d), 124.3 (2d), 122.1 (d), 68.1 (d), 63.9 (t), 47.9 (d), 47.0 (d), 41.5 (d), 38.7 (t);MS *m/z* (%, fragment) (EI): 250; HRMS (*m/z*) for C_18_H_18_O: [M + H]^+^_calcd_ = 251.1357, [M + H]^+^_measured_ = 251.1349.

*(5R,6S,9S)-6-(4-methoxyphenyl)-6,7,8,9-tetrahydro-5H-5,9-methanobenzo[7]anulen-7-ol* (*endo*-**41**): Yield 35%; *R*_f_ (petroleum ether/ dichloromethane): 0.20; ^1^H NMR (CDCl_3_, 600 MHz) δ/ppm 7.85–7.32 (m, 3H, Ar), 6.98–6.24 (m, 5H, Ar), 4.31–4.28 (m, 1H, H_B_) 3.79 (s, 1H, OCH_3_) 3.65–3.63 (m, 1H, H_A/A1/E_) 3.44 (t, 1H, *J* = 4.4 Hz, H_A/A1/E_), 3.36 (t, 1H, *J* = 3.8 Hz, H_A/A1/E_), 3.01 (dd, 1H, *J* = 4.0, 1.4 Hz, H_C/D_), 2,96 (t, 1H, *J* = 5,2 Hz, H_C/D_) 2,38 (d, 1H, *J* = 11,0 Hz, H_G_), 2,27 (s, 1H, OH), 2,05–2,00 (m, 1H, H_F_); MS *m*/*z* (%, fragment) (EI): 280; HRMS (*m*/*z*) for C_19_H_20_O_2_: [M + H]^+^_calcd_ = 281.1463, [M + H]^+^_measured_ = 281.1609.

### 3.7. Synthesis of Ethers from Alcohols

Alcohol obtained after epoxide cleavage (0.05 g) was dissolved in acetone (2 mL), K_2_CO_3_ (1.5 eq) and corresponding alkyl bromides (1.1 eq) were added. The reaction mixture was stirred over 24 h at 56 °C. After the reaction was complete, water and diethylether were added. The organic layer was dried over MgSO_4_, filtered and evaporated to obtain a crude product. The crude material was purified on column chromatography using petroleum ether and dichloromethane as eluents.


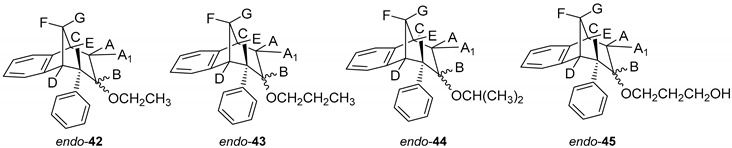


*(5R,6S,9S)-7-Ethoxy-6-phenyl-6,7,8,9-tetrahydro-5H-5,9-methanobenzo[7]annulene (endo-***42**): Yield 20%; *R*_f_ (petroleum ether/dichloromethane): 0.52; ^1^H NMR (CDCl_3_, 600 MHz) δ/ppm: 7.72 (d, 1H, *J* = 7.3 Hz), 7.33–7.19 (m, 4H), 7.05–7.01(m, 2H), 7.14 (t, 1H, *J* = 7.4 Hz), 6.74 (t, 1H, *J* = 7.3 Hz), 4.16–4.11 (m, 2H), 3.35–3.30 (m, 2H), 3.01 (d, 1H, *J* = 11.0 Hz), 2.18–2.13 (m, 1H), 2.09–2.07 (m, 1H), 1.89 (dd, 2H, *J* = 15.2, 4.5 Hz), 1.76–1.73 (t, 3H, *J* = 6.9 Hz, CH_3_); MS *m/z* (%, fragment) (EI): 280 (100).

*(5R,6S,9S)-6-Phenyl-7-propoxy-6,7,8,9-tetrahydro-5H-5,9-methanobenzo[7]annulene* (*endo-***43**): Yield 40%; *R*_f_ (petroleum ether/dichloromethane): 0.57; ^1^H NMR (CDCl_3_, 600 MHz) δ/ppm: 7.42 (t, 1H, *J* = 7.5 Hz), 7.33 (d,1H, *J* = 7.1 Hz), 7.28 (d, 2H, *J* = 7.9 Hz), 7.23 (t, 2H, *J* = 7.5 Hz), 7.14 (t, 2H, *J* = 7.9 Hz), 6.48 (d, 1H, *J* = 7.1 Hz), 4.68–4.63 (m, 2H), 4.19–4.14 (m, 2H), 3.31 (m, 2H), 3.03 (d, 1H, *J* = 11.0 Hz), 2.18–2.13 (m, 1H), 2.09–2.07 (m, 1H), 1.89 (dd, 2H, *J* = 15.2; 4.5 Hz), 1.71 (d, 1H, *J* = 10.5 Hz), 1.28 (t, 3H, *J* = 6.9 Hz, CH_3_); ^13^C NMR (CDCl_3_, 150 MHz) δ/ppm: 145.3 (s), 145.1 (s), 144.3 (s), 127.6 (d), 127.5 (2d), 126.9 (d), 126.6 (2d), 125.7 (d), 124.5 (d), 122.5 (d), 68.8 (d), 53.3 (d), 47.5 (t), 46.8 (d), 39.7 (t), 36.9 (t), 33.8 (t), 32.4 (d), 25.5 (q);MS *m*/*z* (%, fragment) (EI): 292 (100); HRMS (*m*/*z*) for C_21_H_24_O: [M + H]^+^_calcd_ = 293.1827, [M + H]^+^_measured_ = 293.1846.

*(5R,6S,9S)-7-Isopropoxy-6-phenyl-6,7,8,9-tetrahydro-5H-5,9-methanobenzo[7]annulene* (*endo*-**44**): Yield 50%; *R*_f_ (petroleum ether/dichloromethane) = 0.55; ^1^H NMR (CDCl_3_, 600 MHz) δ/ppm: 7.33 (t, 1H, *J* = 7.0 Hz), 7.30–7.28 (m, 1H), 7.23 (t, 3H, *J* = 7.7 Hz), 7.14 (d, 1H, *J* = 7.3 Hz), 6.98 (d, 1H, *J* = 7.3 Hz), 6.47 (d, 2H, *J* = 7.7 Hz), 4.19–4.14 (m, 1H), 3.50–3.46 (m, 1H, isopropyl-CH), 3.24–3.20 (m, 2H), 2.98 (d, 1H, *J* = 11.1 Hz), 2.17–2.12 (m, 1H), 2.06–2.01 (m, 1H), 1.81 (dd, 1H, *J* = 15.0; 4.3 Hz), 1.67 (d, 1H, *J* = 15.0 Hz), 1.29 (broad s, 6H);^13^C NMR (CDCl_3_, 150 MHz) δ /ppm: 145.4 (s), 145.2 (s), 144.3 (s), 127.5 (2d), 126.9 (d), 126.8 (d), 126.5 (2d), 125.7 (d), 124.8 (d), 122.5 (d), 68.8 (d), 53.3 (d), 46.8 (d), 43.2 (d), 39.7 (d), 36.9 (t), 34.1 (t), 27.4 (2q); MS *m*/*z* (%, fragment) (EI): 292 (100); HRMS (*m*/*z*) for C_21_H_24_O: [M + H]^+^_calcd_ = 293.1827, [M + H]^+^_measured_ = 293.1814.

*3-(((5R,6S,9S)-6-Phenyl-6,7,8,9-tetrahydro-5H-5,9-methanobenzo[7]annulen-7-il)oxy)propan-1-ol* (*endo*-**45**): Yield 45%; *R*_f_ (petroleum ether/dichloromethane): 0.50; ^1^H NMR (CDCl_3_, 600 MHz) δ/ppm 7.28 (d, 1H, *J* = 7.3 Hz, Ar), 7.21–7.18 (m, 3H, Ar), 7.15 (t, 1H, *J* = 7.3 Hz, Ar), 6.92 (t, 1H, *J* = 7.3 Hz, Ar), 6.69 (dd, 2H, *J* = 6.6; 3.3 Hz, Ar), 6.24 (d, 1H, *J* = 7.3 Hz, Ar), 3.57–3.44 (m, 4H), 3.52 (d, 1H, *J* = 4.8 Hz, H_B_), 3.44 (t, 1H, *J* = 4.2 Hz, H_E_), 3.37 (t, 1H, *J* = 3.8 Hz, H_A/A1_), 3.05 (dd, 1H, *J* = 4.2 Hz, H_C_), 2.98 (t, 1H, *J* = 5.1 Hz, H_D_), 2.40 (d, 1H, *J* = 10.7 Hz, H_G_), 2.11–2.06 (m, 1H, H_A/A1_), 2.06–2.01 (m, 1H, H_F_), 1.42 (s, 1H, OH), 1.24 (broad s, 2H); ^13^C NMR (CDCl_3_, 150 MHz) δ /ppm: 145.2 (s), 143.5 (s), 141.7 (s), 128.6 (2d), 127.7 (2d), 126.8 (d), 126.3 (d), 126.0 (d), 125.7 (d), 122.5 (d), 68.2 (d), 54.5 (t), 52.9 (d), 50.4 (t), 45.9 (d), 43.7 (d), 40.6 (t), 39.9 (t), 35.5 (t); MS *m*/*z* (%, fragment) (EI): 308; HRMS (*m*/*z*) for C_21_H_24_O_2_: [M + H]^+^_calcd_ = 309.1776, [M + H]^+^_measured_ = 309.4140.


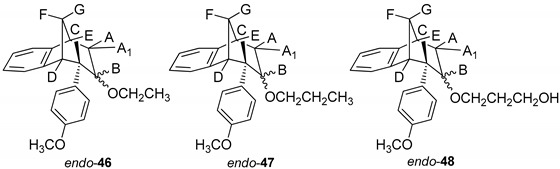


*(5R,6S,9S)-7-Ethoxy-6-(4-methoxyphenyl)-6,7,8,9-tetrahydro-5H-5,9-methanobenzo-[7]annulene* (*endo*–**46**): Yield 20%; *R*_f_ (petroleum ether/dichloromethane): 0.67; ^1^H NMR (CDCl_3_, 600 MHz) δ/ppm 7.30 (d, 1H, *J* = 7.5 Hz), 7.17 (t, 1H, *J* = 7.5 Hz), 6.96 (t, 1H, *J* = 7.5 Hz), 6.76 (d, 2H, *J* = 8.6 Hz), 6.62 (d, 2H, *J* = 8.6 Hz), 6.33 (d, 1H, *J* = 7.5 Hz), 3.79 (s, 3H), 3.51–3.48 (m, 2H), 3.45 (t, 1H, *J* = 4.8 Hz), 3.38 (t, 1H, *J* = 4.2 Hz), 3.03 (d, 1H, *J* = 4.2 Hz), 2.97 (t, 1H, *J* = 4.8 Hz), 2.38-2.33 (m, 2H), 2.39 (d, 1H, *J* = 10.8 Hz), 2.06–2.01 (m, 1H), 1.27 (s, 3H); MS *m*/*z* (%, fragment) (EI): 308 (100).

*(5R,6S,9S)-6-(4-Methoxyphenyl)-7-propoxy-6,7,8,9-tetrahydro-5H-5,9-methanobenzo[7]annulene* (*endo*-**47**): Yield 35%; *R*_f_ (petroleum ether/dichloromethane):0.57; ^1^H NMR (CDCl_3_, 600 MHz) δ/ppm 7.21 (d, 1H, *J* = 7.3 Hz), 7.08 (t, 1H, *J* = 7.4 Hz), 6.87 (t, 1H, *J* = 7.4 Hz), 6.67 (d, 2H, *J* = 8.6 Hz), 6.53 (d, 2H, *J* = 8.6 Hz), 6.24 (d, 1H, *J* = 7.3 Hz), 3.70 (s, 3H, OCH_3_), 3.51 (q, 2H, *J* = 6.6 Hz), 3.40 (t, 1H, *J* = 4.8 Hz), 3.36 (t, 1H, *J* = 4.3 Hz), 2.93 (d, 1H, *J* = 3.7 Hz), 2.87 (t, 1H, *J* = 4.9 Hz), 2.39–2.35 (m, 4H), 2.30 (d, 1H, *J* = 10.8 Hz), 1.99–1.96 (m, 1H), 1.18 (broad s, 3H); ^13^C NMR (CDCl_3_, 150 MHz) δ/ppm: 157.5 (s), 144.5 (s), 143.2 (s), 133.5 (s), 129.2 (d), 127.1 (d), 126.4 (2d), 126.2 (d), 122.1 (2d), 112.6 (d), 54.7 (d), 54.0 (d), 52.7 (d), 45.5 (d), 42.4 (d), 40.1 (d), 34.7 (t), 30.4 (q); MS *m*/*z* (%, fragment) (EI): 322; HRMS (*m*/*z*) for C_22_H_26_O_2_: [M + H]^+^_calcd_ = 323.1932, [M + H]^+^_measured_ = 323.4406.

*3-(((5R,6S,9S)-6-(4-Methoxyphenyl)-6,7,8,9-tetrahydro-5H-5,9-methanobenzo-[7]annulen-7-il)oxy)propan-1-ol* (*endo*-**48**): Yield 15%; *R*_f_ (petroleum ether/dichloromethane): 0.64; ^1^H NMR (CDCl_3_, 600 MHz) δ/ppm 6.95 (d, 1H, *J* = 7.4 Hz), 6.82 (t, 1H, *J* = 7.3 Hz), 6.75 (t, 1H, *J* = 7.3 Hz), 6,71 (d, 2H, *J* = 8,5 Hz, Ar), 6,63 (d, 1H, *J* = 7,4 Hz, Ar), 6,46 (d, 2H, *J* = 8,6 Hz, Ar), 4,20–4,16 (m, 1H), 3,72–3,66 (m, 4H, CH_2_CH_2_), 3,64 (s, 3H, OCH_3_), 3,53 (t, 1H, *J* = 4,8 Hz), 3,31–3,25 (m, 1H), 2,96 (s, 1H, OH), 2,57 (dt, 1H, *J* = 14,3; 3,4 Hz), 2,44–2,39 (m, 1H), 2,28–2,23 (m, 1H), 2,04–1,97 (m, 2H), 1,17 (broad s, 2H); MS *m*/*z* (%, fragment) (EI): 338; HRMS (*m*/*z*) for C_22_H_26_O_3_: [M + H]^+^_calcd_ = 339.1881, [M + H]^+^_measured_ = 339.4404.

### 3.8. Friedel-Crafts Acylation

To the solution of compound 49 [41] (100 mg, 0.510 mol) in dichloromethane (2 mL), AlCl_3_ (68 mg, 0.510 mol) and different carbonyl chlorides (1.1 eq) were added. The reaction was performed in a sealed tube and left overnight at room temperature. After removal of the solvent, the residue crude product was purified by repeated column chromatography using petroleum ether/dichloromethane mixture as eluent. All of the obtained compounds **50**–**57** were isolated in the first fractions. 


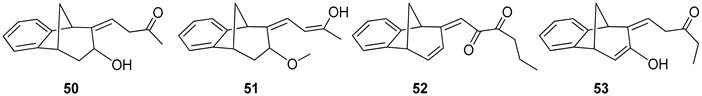


*(Z)-4-((5R,9R)-7-Hydroxy-5,7,8,9-tetrahydro-6H-5,9-methanobenzo[7]annulen-6-ilydene)butan-2-one* (**50**): 16 mg (13%); yellow oil; *R*_f_ (dichloromethane): 0.5;^1^H NMR (CDCl_3_, 600 MHz) δ/ppm: 7.54 (d, 1H, *J* = 7.7 Hz, H_Ar_), 7.29 (t, 1H, *J* = 7.7 Hz, H_Ar_), 7.14 (t, 1H, *J* = 7.7 Hz, H_Ar_), 7.10 (d, 1H, *J* = 7.7 Hz, H_Ar_), 6.79 (m, 1H), 3.83 (m, 1H), 3.62 (d, 1H, *J* = 3.6 Hz), 3.24 (t, 1H, *J* = 6.6 Hz), 3.01 (d, 1H, *J* = 4.6 Hz), 2.45 (m, 1H), 2.28 (s, 1H, -OH), 2.31 (d, 1H, *J* = 10.7 Hz,), 2.29 (m, 2H), 2.06–2.02 (m, 1H), 1.95 (s, 3H); MS *m*/*z* (%, fragment) (EI): 242 (100); HRMS (*m*/*z*) for C_16_H_18_O_2_: [M + H]^+^_calcd_ = 243.1306, [M + H]^+^_measured_ = 243.3129.

*(5R,9R,Z)-6-((Z)-3-Hydroxybut-2-ene-1-ilydene)-6,7,8,9-tetrahydro-5H-5,9-methano-benzo[7]annulen-7-ol* (**51**): 30 mg (30%); yellow oil; *R*_f_ (dichloromethane/petroleum ether 70%): 0.10; ^1^H NMR (CDCl_3_, 600 MHz) δ/ppm: 7.30 (d, 1H, *J* = 7.5 Hz, H_Ar_), 7.16 (t, 1H, *J* = 7.5 Hz, H_Ar_), 6.93 (t, 1H, *J* = 7.5 Hz, H_Ar_), 6.70 (m, 2H), 6.26 (d, *J* = 7.3 Hz, 1H), 3.53 (d, 1H, *J* = 5.1 Hz), 3.45 (t, 1H, *J* = 4.4 Hz), 3.38 (t, 1H, *J* = 3.9 Hz), 3.06 (dd, 1H, *J* = 4.0; 1.5 Hz), 2.99 (t, 1H, *J* = 5.1 Hz), 2.41 (d, 1H, *J* = 10.9 Hz), 2.10 (s, 1H, OH), 2.06–2.02 (m, 1H), 1.53 (s, 3H); MS *m*/*z* (%, fragment) (EI): 242 (100); HRMS (*m*/*z*) for C_16_H_18_O_2_: [M + H]^+^_calcd_ = 243.1306, [M + H]^+^_measured_ = 243.3131.

*(E)-1-((5S,9R)-5,9-Dihydro-6H-5,9-methanobenzo[7]annulen-6-ilydene)hexa-2,3-dione* (**52**): 21 mg (15%); yellow oil; *R*_f_ (dichloromethane): 0.40; ^1^H NMR (CDCl_3_, 600 MHz) δ/ppm: 8.07 (d, 1H, *J*= 7.9 Hz, H_Ar_), 7.83 (d, 1H, *J* = 7.9 Hz, H_Ar_), 7.13–7.09 (m, 2H), 6.26 (dd, 1H, *J* = 6.8; 1.8 Hz), 6.02 (d, 1H, *J* = 9.6 Hz), 5.91 (d, 1H, *J* = 9.6 Hz), 4.25–4.19 (m, 2H), 3.55–3.51 (m, 2H), 2.71 (d, 1H, *J* = 11.2 Hz), 2.35–2.31 (m, 2H), 2.04–2.01 (m, 1H), 1.63 (s, 3H); MS *m*/*z* (%, fragment) (EI): 266 (100); HRMS (*m*/*z*) for C_18_H_18_O_2_: [M + H]^+^_calcd_ = 267.1306, [M + H]^+^_measured_ = 267.3343.

*(Z)-1-((5R,9R)-7-Hydroxy-5,9-dihydro-6H-5,9-methanobenzo[7]annulen-6-ilydene)-pentan-3-one* (**53**): 76 mg (59%); yellow oil; *R*_f_ (dichloromethane): 0.29; ^1^H NMR (CDCl_3_, 600 MHz) δ/ppm: 8.04 (d, 1H, *J* = 8.5 Hz, H_Ar_), 7.93 (d, 1H, *J* = 9.1 Hz, H_Ar_), 7.50 (t, 1H, *J* = 7.7 Hz, H_Ar_), 7.16 (m, 1H, H_Ar_), 6.30 (dd, 1H, *J* = 3.1; 1.9 Hz), 6.10 (d, 1H, *J* = 3.1 Hz), 4.59 (d, 1H, *J* = 6.3 Hz), 4.42 (d, 1H, *J* = 5.6 Hz), 4.33 (s, 1H, OH), 4.10–4.07 (m, 2H), 2.28 (d, 1H, *J* = 11.5 Hz), 1.93–1.83 (m, 1H) 1.80–1.69 (m, 2H), 1.00 (t, 3H, *J* = 5.4 Hz); MS *m*/*z* (%, fragment) (EI): 254 (100); HRMS (*m/z*) for C_17_H_18_O_2_: [M + H]^+^_calcd_ = 255.1306, [M + H]^+^_measured_ = 255.3236.


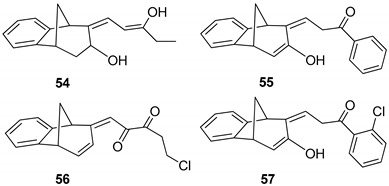


*(9Z)-9-[(Z)-3-Hydroxypent-2-enilydene]tricyclo[6.3.1.02,7]dodeca-2(7),3,5-trien-10-ol* (**54**): 8.9 mg (12%); yellow oil; *R*_f_ (dichloromethane/petroleum ether 20%): 0.73; ^1^H NMR (CDCl_3_, 600 MHz) δ/ppm: 7.44–7.02 (m, 4H), 6,64 (m, 1H), 6.27 (d, 1H, *J* = 7.8 Hz), 3.63 (q, 2H, *J* = 6.8 Hz), 3.51 (d, 1H, *J* = 4.5 Hz), 3.44 (t, 1H, *J* = 4.5 Hz), 3.37 (t, 1H, *J* = 4.5 Hz), 2.99 (dd, 1H, *J* = 3.0; 1.5 Hz), 2.95 (t, 1H, *J* = 4.5 Hz), 2.38 (d, 1H, *J* = 10.6 Hz), 2.05-2.02 (m, 1H), 1.56 (t, 3H, *J* = 7.2 Hz); MS *m*/*z* (%, fragment) (EI): 256 (100).

*(Z)-3-((5R,9R)-7-Hydroxy-5,9-dyhidro-6H-5,9-methanobenzo[7]annulen-6-ilydene)-1-phenylpropan-1-one* (**55**): 29.4 mg (19%); colourless oil; *R*_f_ (dichloromethane): 0.38; ^1^H NMR (CDCl_3_, 600 MHz) δ/ppm: 8.01 (d, 2H, *J* = 8.7 Hz, H_Ar_), 7.90 (t, 2H, *J* = 8.9 Hz, H_Ar_), 7.71 (d, 1H, *J* = 8.3 Hz, H_Ar_), 7.68 (d, 1H, *J* = 9.2 Hz, H_Ar_), 7.50–7.43 (m, 3H, H_Ar_), 6.30 (m, 1H), 6.10 (d, 1H, *J* = 3.3 Hz), 4.71 (s, 1H, OH), 4.58 (d, 1H, *J* = 4.3 Hz), 4.39 (d, 1H, *J* = 4.9 Hz), 4.12 (t, 2H, *J* = 4.9 Hz), 2.40 (d, 1H, *J* = 10.5 Hz), 2.33–2.28 (m, 1H); MS *m*/*z* (%, fragment) (EI): 302 (100).

*(E)-5-Chloro-1-((5S,9R)-5,9-dyhdro-6H-5,9-methanobenzo[7]annulen-6-ilydene)penta-2,3-dione* (**56**): 7.5 mg (7.3%); colorless oil; *R*_f_ (dichloromethane): 0.42; ^1^H NMR (CDCl_3_, 600 MHz) δ/ppm: 8.01 (d, 1H, *J* = 7.6 Hz, H_Ar_), 7.13 (d, 1H, *J* = 7.6 Hz, H_Ar_), 7.10–7.05 (m, 2H, H_Ar_), 6.26 (dd, 1H, *J* = 6.5; 1.6 Hz), 6.14 (d, 1H, *J* = 9.3 Hz), 5.98 (d, 1H, *J* = 9.3 Hz), 4.25–4.19 (m, 2H), 3.55 (m, 2H), 2.71 (d, 1H, *J* = 11.1 Hz), 2.33 (m, 2H), 2.07–2.03 (m, 1H); MS *m*/*z* (%, fragment) (EI): 286 (100), 288 (32).

*(Z)-1-(2-Chlorophenyl)-3-((5R,9R)-7-hydroxy-5,9-dihydro-6H-5,9-methanobenzo-[7]annulen-6-ilydene)propan-1-one* (**57**): 9.5 mg (8%); colorless oil; *R*_f_ (dichloromethane): 0.40; ^1^H NMR (CDCl_3_, 600 MHz) δ/ppm: 8.02 (d, 1H, *J* = 8.9 Hz, H_Ar_), 7.90 (t, 2H, *J* = 9.2 Hz, H_Ar_), 7.81 (d, 1H, *J* = 8.3 Hz, H_Ar_), 7.70 (d, 1H, *J* = 9.2 Hz, H_Ar_), 7.50–7.43 (m, 3H, H_Ar_), 6.28 (dd, 1H, *J* = 3.2; 1.8 Hz), 6.08 (d, 1H, *J* = 3.1 Hz), 4.55 (d, 1H, *J* = 6.1 Hz), 4.21 (d, 1H, *J* = 5.7 Hz), 3.98 (t, 2H, *J* = 6.1 Hz), 3.18 (s, 1H, -OH), 2.03–1.99 (m, 2H); MS *m*/*z* (%, fragment) (EI): 336 (100), 338 (32).

### 3.9. Reversible Inhibition of Cholinesterases by Novel Compounds

Inhibition of novel compounds was evaluated for recombinant human AChE and BChE derived from purified human plasma (a generous gift from Dr Florian Nachon, *Département de Toxicologie et Risques Chimiques, Institut de Recherche Biomédicale des Armées*, Bretigny-sur-Orge, France). The inhibition mixture contained a 0.1 M phosphate buffer, pH 7.4, enzyme (4.5 nM BChE or 0.2 nM AChE) tested compound, and DTNB (0.3 mM; Sigma Chemical Co., St. Louis, MO, USA). Enzyme activity was measured upon addition of ATCh (0.2 or 0.1 mM; Sigma Chemical Co., St. Louis, MO, USA) by the Ellman method [45] at 25 °C and 412 nm, on a Tecan Infinite M200PRO plate reader (Tecan Austria, GmbH, Salzburg, Austria). Due to the low solubility, a 100 mM stock solution of the tested compounds was prepared in DMSO, and the same solvent was in controls as well. The IC_50_ values were determined from at least three experiments by a nonlinear fit of the compound concentration logarithm values vs.% of enzyme activity using Prism6 software (GraphPad Prism 6 Software, San Diego, USA).

## 4. Conclusions

The photochemical synthesis resulted in four groups of molecules with different functionalities on the methano-bridged benzobicyclo[3.2.1]octadiene skeleton. The majority of the compounds were more selective for BChE than for AChE, although their inhibitory potency varied with compound functionalities. The structure-activity relationship exhibited an increase in inhibition potency for both enzymes by elimination of the *endo*-benzylamine substituent. Although none of the tested compounds inhibited in nanomolar range, benzobicyclo[3.2.1]octene compounds **51** and **31** arose as lead compounds for the future development of new AChE and BChE inhibitors, respectively. Therefore, for future design of cholinesterase inhibitors and search for therapeutics of neurological disorders, compounds with a benzobicyclo[3.2.1]octene skeleton, a hydroxyl group on it, and an opened furan ring, should be taken into consideration.

## Figures and Tables

**Table 1:** Inhibition of BChE by benzobicyclo[3.2.1]octadiene benzylamines expressed as IC_50_ ± SEM.

Compound	IC_50_/µM
*endo*-**4**	110 ± 77
*endo*-**7**	≥500
*endo*-**8**	>500
*endo*-**9**	>500
*endo*-**10**	63 ± 13
*endo*-**11**	≥500
*endo*-**13**	>500
*endo*-**14**	>500
*endo*-**15**	>500
*endo*-**16**	>500
*endo*-**17**	>500
*endo*-**18**	>500
*endo*-**19**	>500
*endo*-**20**	>500
**1**	260 ± 120
**9**	240 ± 210
**17**	120 ± 64
**20**	182 ± 98

**Table 2:** Inhibition of BChE by oximes and oxime ethers with benzobicyclo[3.2.1]-skeleton expressed as IC_50_ ± SEM.

**Compound**	**IC_50_/µM**
**22**	25 ± 6
**23**	450 ± 160
**24**	350 ± 100
**25**	>500
**26**	380 ± 250
**27**	74 ± 19
**28**	24 ± 3
**29**	100 ± 32
**30**	61 ± 36
**31**	90 ± 40
**32**	31 ± 4
**33**	140 ± 49
**34**	78 ± 31
**35**	60 ± 11

**Table 3:** Inhibition of human AChE and BChE by epoxides, alcohols and ethers possessing benzobicyclo[3.2.1]-skeleton expressed as IC_50_ ± SEM.

Compounds	IC_50_/µM	
	AChE	BChE
*endo*-**38**	62 ± 8	22 ± 5
*endo*-**39**	>100	33 ± 6
*endo*-**43**	>100	17 ± 2
*endo*-**45**	>100	90 ± 24
*endo*-**46**	270 ± 110	>500
*endo*-**47**	>100	>200

**Table 4:** Inhibition of human AChE and BChE by acylated furo-benzobicyclo [3.2.1]octenes/octadienes expressed as IC_50_ ± SEM.

Compound	IC_50_/µM	
	AChE	BChE
**51**	8.3 ± 4.0	>300
**53**	188 ± 133	146 ± 30
Ethopropazine	73 ± 8.3	0.046 ± 0.037

## Supplementary Materials

The following are available online. Figures S1–S31 with ^1^H NMR, ^13^C NMR, COSY, LR COSY, and NOESY spectrum of prepared compounds, and Cartesian coordinates of optimized geometries of reactants, transition states, and products of reactions 1–4.
