# Supplementary material for: Benzobicyclo[3.2.1]octene Derivatives as a New Class of Cholinesterase Inhibitors
Source: Molecules. 2020 Oct 22;25(21):4872. doi: 10.3390/molecules25214872 (PMC7659976; doi:10.3390/molecules25214872)
Supplement: Supplementary file 1 [file molecules-25-04872-s001.pdf]

molecules

MDPI

Supplemental Material

Benzobicyclo[3.2.1]octene derivatives as a new class of cholinesterase inhibitors

Tena Čadež[1§], Ana Grgičević[2§], Ramiza Ahmetović[2], Danijela Barić[3], Nikolina Maček Hrvat[1], Zrinka Kovarik[1*] and Irena Škorić[2*]

[1] Institute for Medical Research and Occupational Health, Ksaverska cesta 2, 10000 Zagreb, Croatia; tcadez@imi.hr (T.Č); nmacek@imi.hr (N.M.H)
[2] Department of Organic Chemistry, Faculty of Chemical Engineering and Technology, University of Zagreb, Marulićev trg 19, 10000 Zagreb, Croatia; aratkov@fkit.hr (A.G); rahmetov@fkit.hr (R.A)
[3] Division of Physical Chemistry, Rudjer Bošković Institute, Bijenička cesta 54, 10000 Zagreb, Croatia; dbaric@irb.hr (D.B)
§ These authors contributed equally.
* Correspondence: zkovarik@imi.hr (Z.K); iskoric@fkit.hr (I.Š)

Contents

11-(4-chlorophenyl)tricyclo[6.3.1.0²,7]dodeca-2,4,6,9-tetraene (*endo*-1)....2

N-benzyl-4-((5*R*,6*S*,9*S*)-6,9-dihydro-5*H*-5,9-methanobenzo[7]annulen-6-yl)aniline (*endo*-4)....3

4-((5*R*,6*S*,9*S*)-6,9-dihydro-5*H*-5,9-methanobenzo[7]annulen-6-yl)-*N*-(pyridin-3-ylmethyl)aniline (*endo*-6)....4

4-((5*R*,6*S*,9*S*)-6,9-dihydro-5*H*-5,9-methanobenzo[7]annulen-6-yl)-*N*-(fur-2-ylmethyl)aniline (*endo*-7)....5

4-((5*R*,6*S*,9*S*)-6,9-dihydro-5*H*-5,9-methanobenzo[7]annulen-6-yl)-*N*-(thien-2-ylmethyl)aniline (*endo*-8)....6

(4*R*,9*R*)-9,10-dihydro-4*H*-4,9-methanobenzo[4,5]cyclohepta[1,2-b]furan-2-carbaldehyde (21)....7

endo-6-phenyl-6,9-dihydro-5*H*-5,9-methano-benzocycloheptene (*endo*-36)....8

11-(4-methoxyphenyl)tricyclo[6.3.1.0²,⁷]dodeca-2,4,6,9-tetraene (*endo*-37)....10

(2*R*,7*R*,8*S*)-8-phenyl-1a,7,8,8a-tetrahydro-2*H*-2,7-methanobenzo[4,5]cyclohepta[1,2-*b*]-oxirene (*endo*-38)....11

(2*R*,7*R*,8*S*,8a*S*)-8-(4-methoxyphenyl)-1a,7,8,8a-tetrahydro-2*H*-2,7-methanobenzo[4,5] cyclohepta[1,2-*b*]oxirene (*endo*-39)....16

(6*S*)-6-phenyl-6,7,8,9-tetrahydro-5*H*-5,9-methanobenzo[7]anulen-7-ol (*endo*-40)....19

((5*R*,6*S*,9*S*)-6-phenyl-7-propoxy-6,7,8,9-tetrahydro-5*H*-5,9-methanobenzo[7]annulene (*endo*-43)....21

(5*R*,6*S*,9*S*)-7-isopropoxy-6-phenyl-6,7,8,9-tetrahydro-5*H*-5,9-methanobenzo[7]annulene (*endo*-44)....22

3-(((5*R*,6*S*,9*S*)-6-phenyl-6,7,8,9-tetrahydro-5*H*-5,9-methanobenzo[7]annulen-7-il)oxy)propan-1-ol (*endo*-45)....23

(5*R*,6*S*,9*S*)-7-ethoxy-6-(4-methoxyphenyl)-6,7,8,9-tetrahydro-5*H*-5,9-methanobenzo-[7]annulene (*endo*-46)....28

(5*R*,6*S*,9*S*)-6-(4-methoxyphenyl)-7-propoxy-6,7,8,9-tetrahydro-5*H*-5,9-methanobenzo[7] anunlene (*endo*-47)....30

(4R,9R)-9,10-dihydro-4H-4,9-methanobenzo[4,5]cyclohepta[1,2-*b*]furan (49)....36

(5*R*,9*R*,*Z*)-6-((*Z*)-3-hydroxybut-2-ene-1-ilydene)-6,7,8,9-tetrahydro-5*H*-5,9-methano-benzo[7]annulen-7-ol (51)....37

(*Z*)-1-((5*R*,9*R*)-7-hydroxy-5,9-dihydro-6*H*-5,9-methanobenzo[7]annulen-6-ilydene)-pentan-3-one (53)....38

Cartesian coordinates of optimized geometries of reactants, transition states, and products of reactions 1-4....39

11-(4-chlorophenyl)tricyclo[6.3.1.0²,7]dodeca-2,4,6,9-tetraene (*endo*-1)

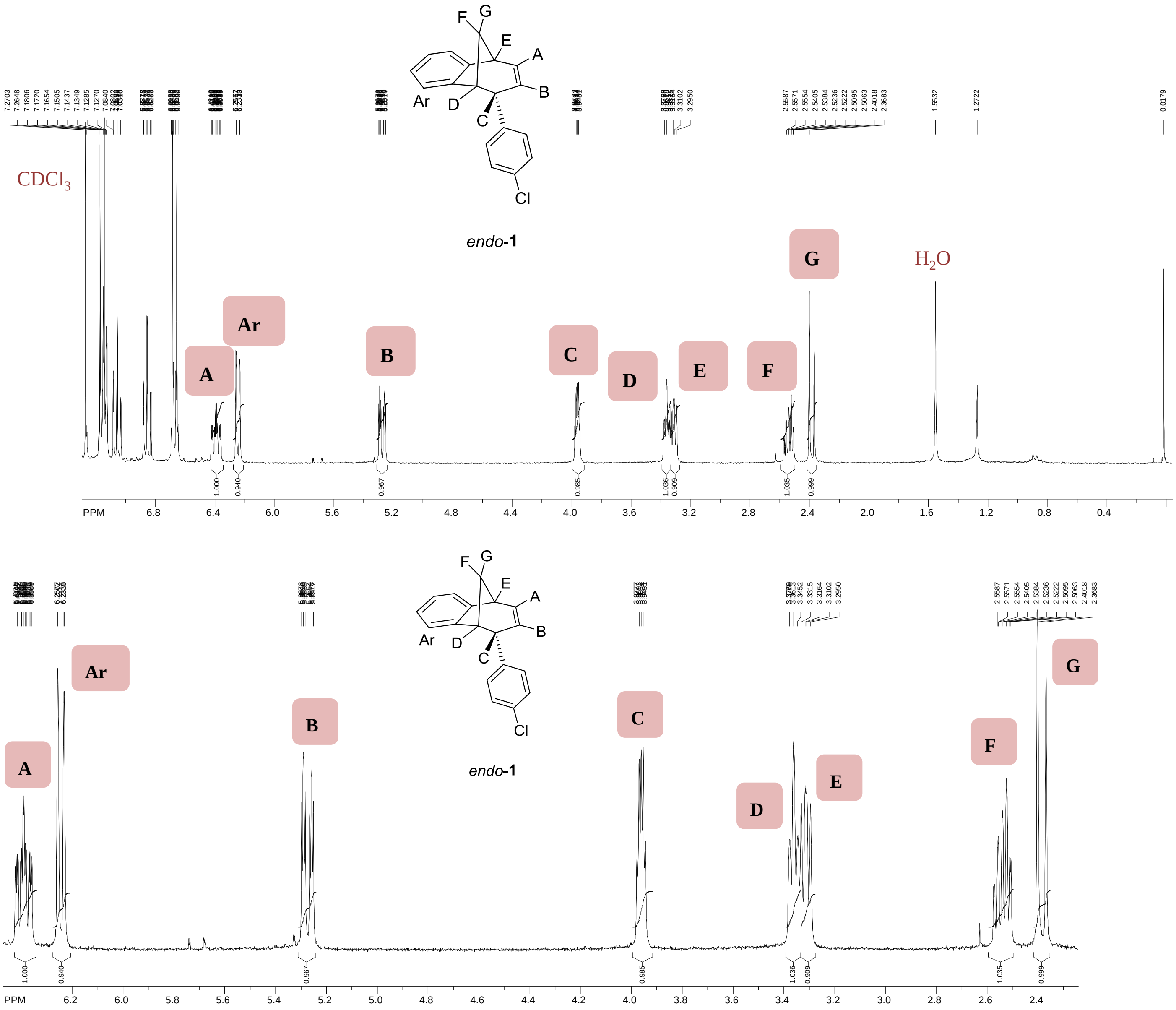

Figure S1. ^{1}H NMR spectrum ($CDCl_3$) of *endo*-**1**.

N-benzyl-4-((5*R*,6*S*,9*S*)-6,9-dihydro-5*H*-5,9-methanobenzo[7]annulen-6-yl)aniline (*endo*-4)

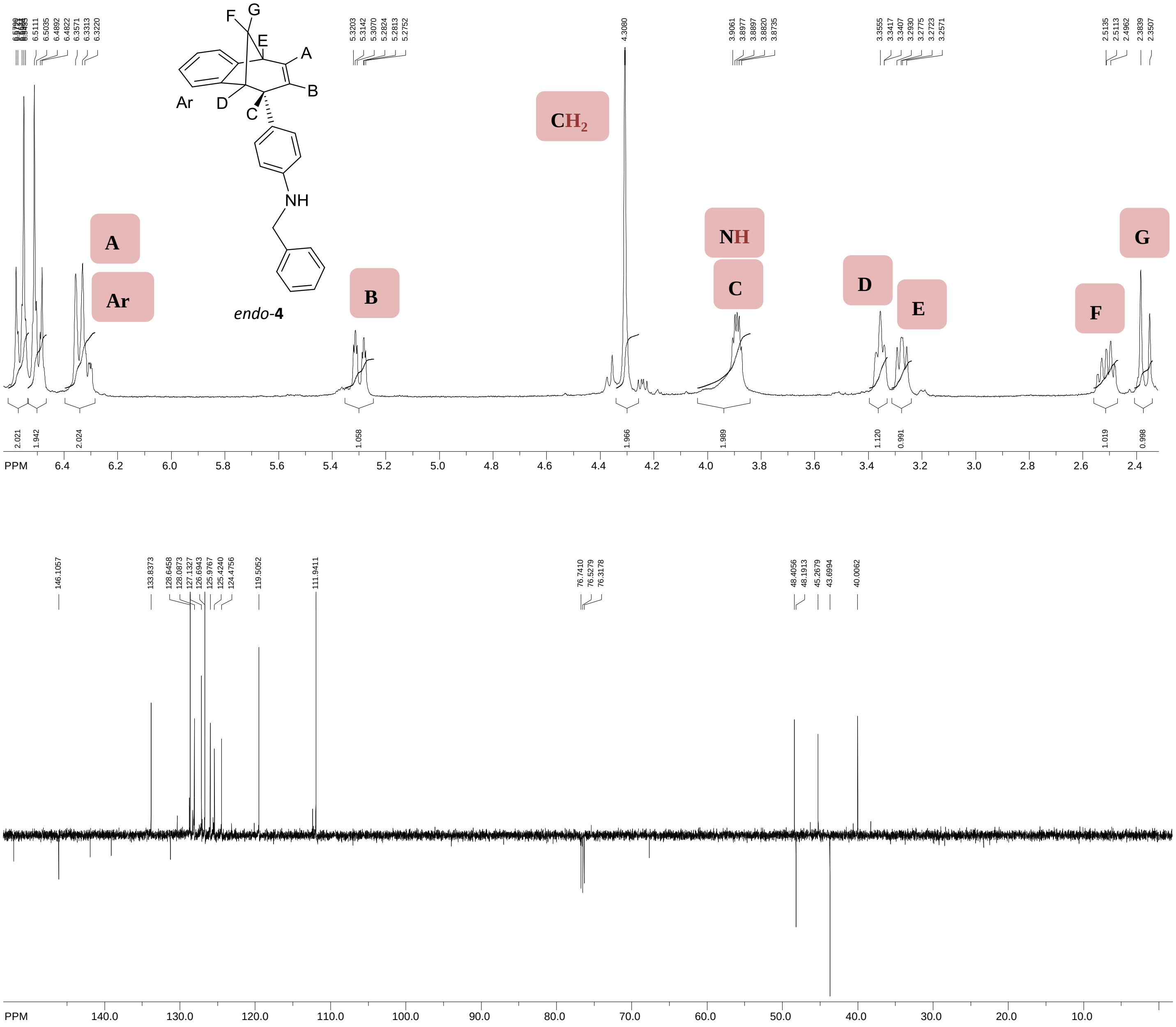

Figure S2. ^1H NMR spectrum (300 MHz, $CDCl_3$; upper panel) and ^{13}C NMR spectrum (150 MHz, $CDCl_3$; lower panel) of *endo*-**4.**

4-((5*R*,6*S*,9*S*)-6,9-dihydro-5*H*-5,9-methanobenzo[7]annulen-6-yl)-*N*-(pyridin-3-ylmethyl)aniline (*endo*-6)

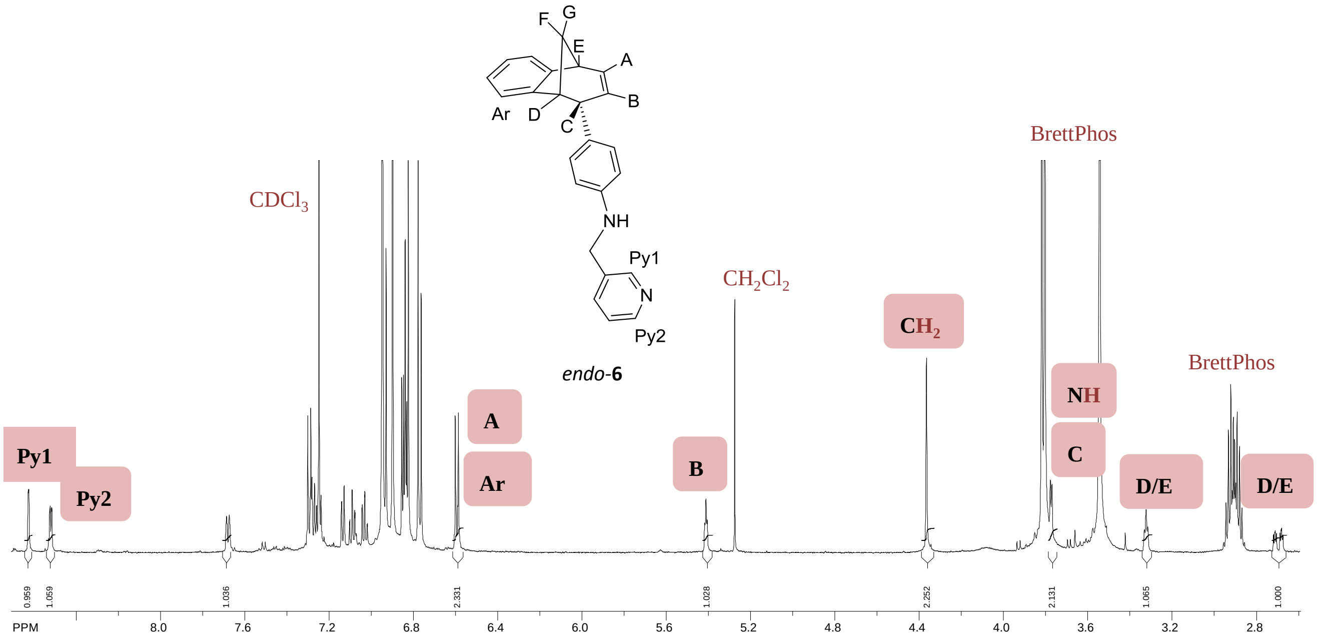

Figure S3. ^{1}H NMR spectrum (300 MHz, $CDCl_3$) of *endo*-**6.**

4-((5*R*,6*S*,9*S*)-6,9-dihydro-5*H*-5,9-methanobenzo[7]annulen-6-yl)-*N*-(fur-2-ylmethyl)aniline (*endo*-7)

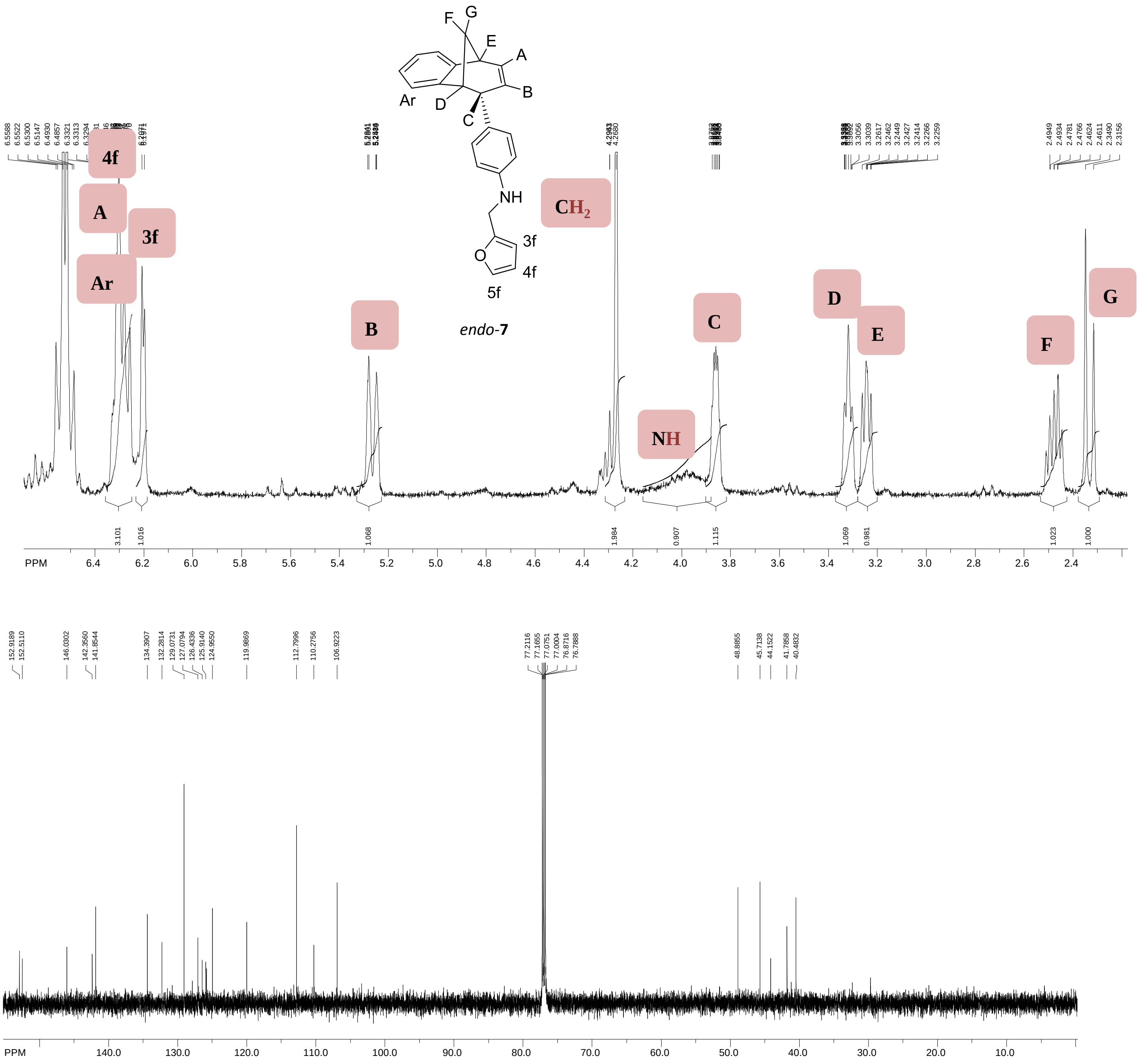

Figure S4. ^{1}H NMR spectrum (300 MHz, $CDCl_3$; upper panel) and ^{13}C NMR spectrum (150 MHz, $CDCl_3$; lower panel) of *endo*-**4.**

4-((5*R*,6*S*,9*S*)-6,9-dihydro-5*H*-5,9-methanobenzo[7]annulen-6-yl)-*N*-(thien-2-ylmethyl)aniline (*endo*-8)

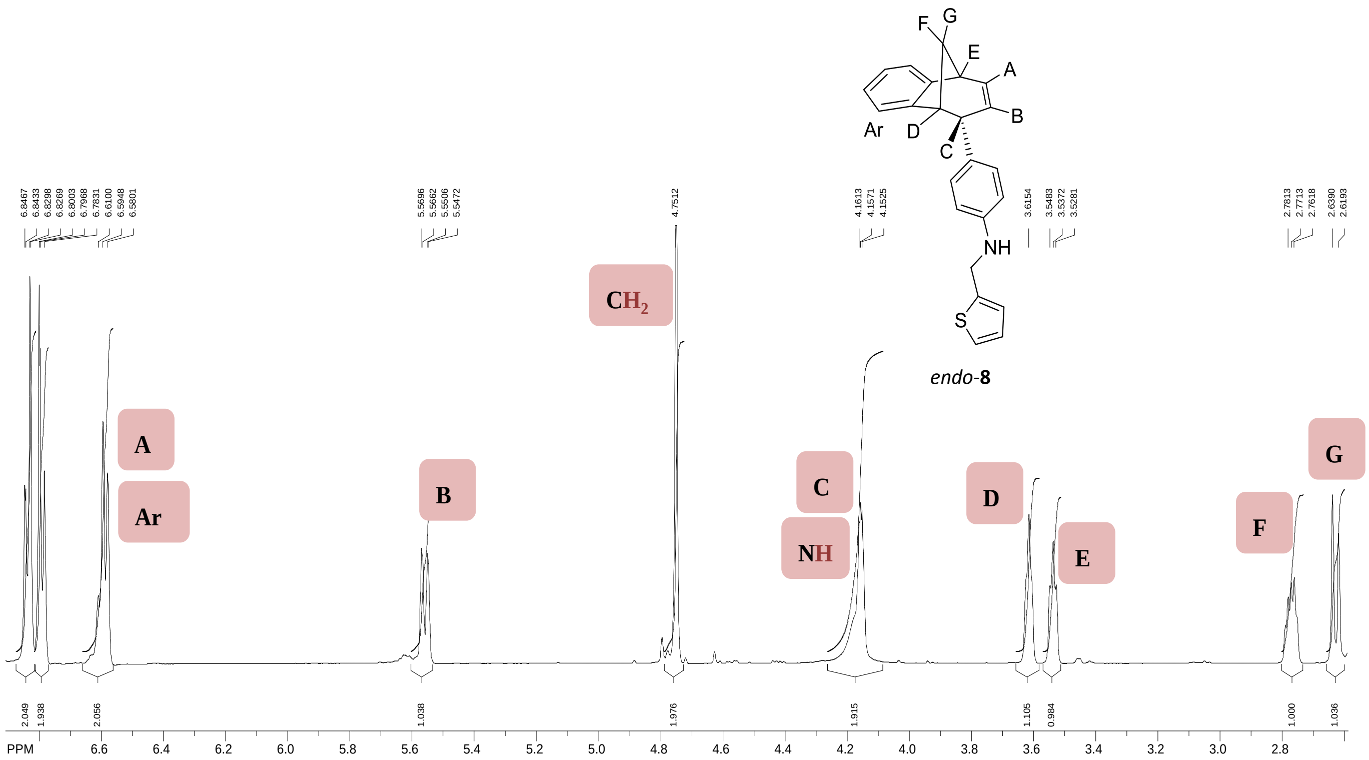

Figure 5S. ^{1}H NMR spectrum (600 MHz, $CDCl_3$) of *endo*-**8.**

(4*R*,9*R*)-9,10-dihydro-4*H*-4,9-methanobenzo[4,5]cyclohepta[1,2-b]furan-2-carbaldehyde (21)

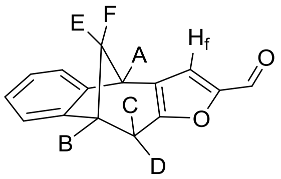

21

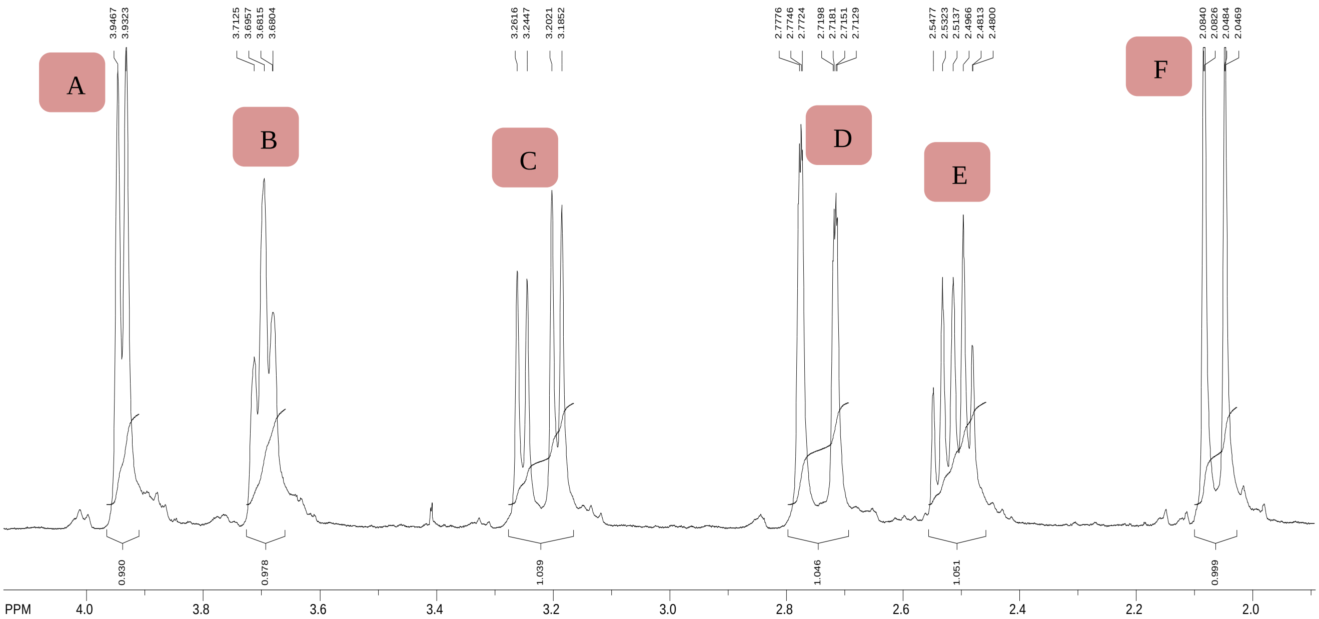

Figure S6. ^{1}H NMR spectrum (600 MHz, $CDCl_3$) of **21.**

endo-6-phenyl-6,9-dihydro-5*H*-5,9-methano-benzocycloheptene (*endo*-36)

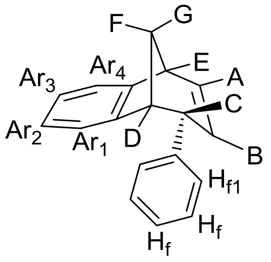

endo-**36**

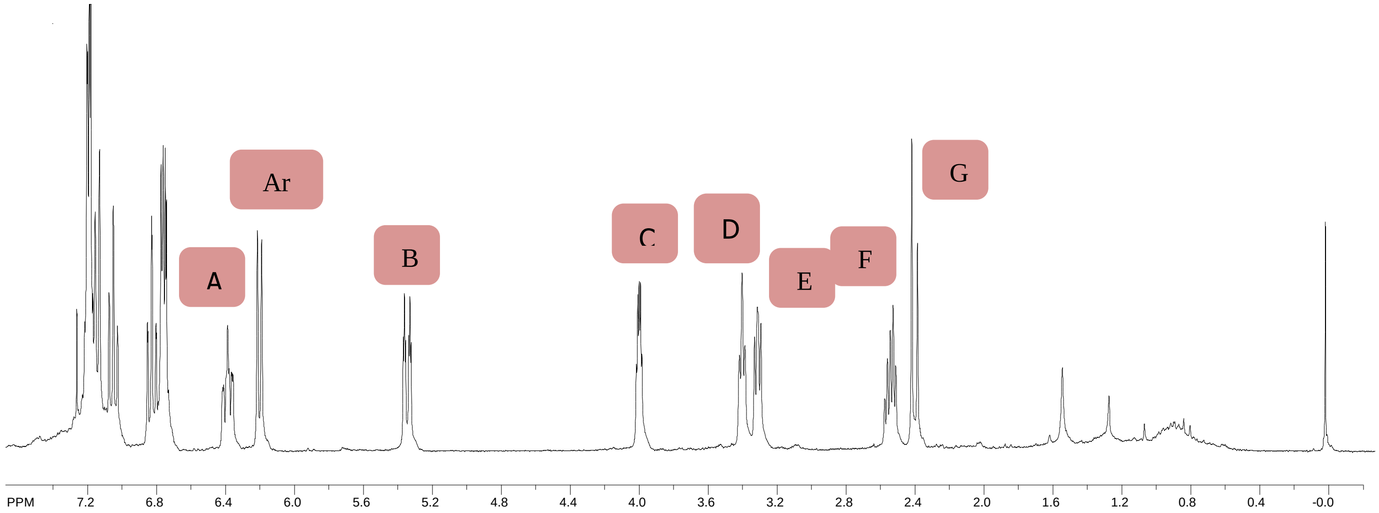

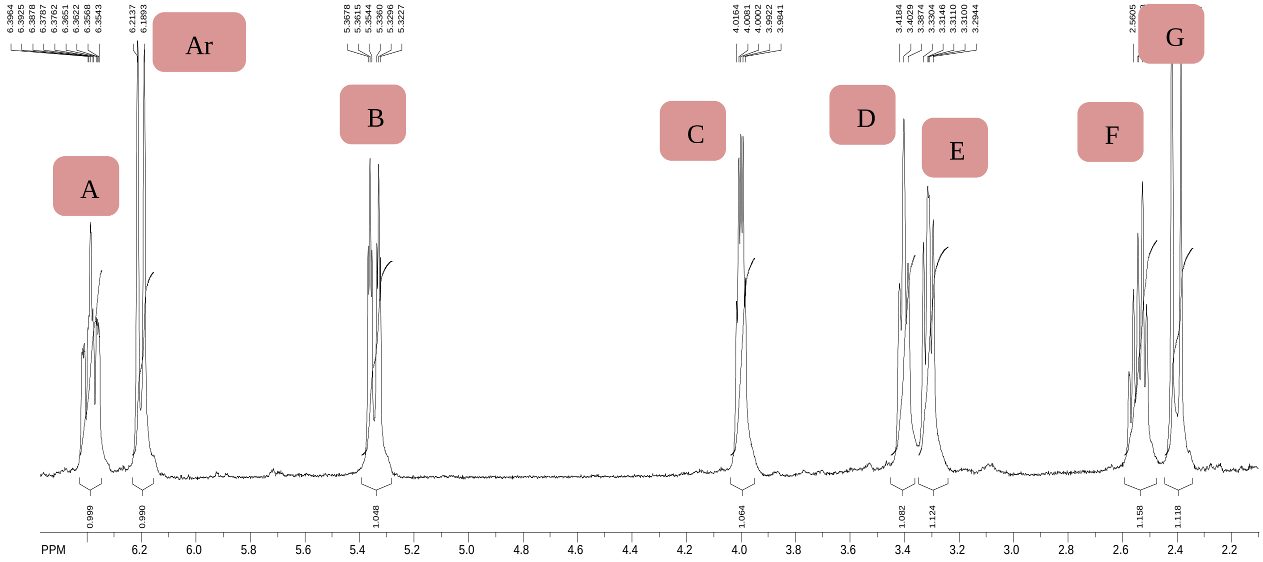

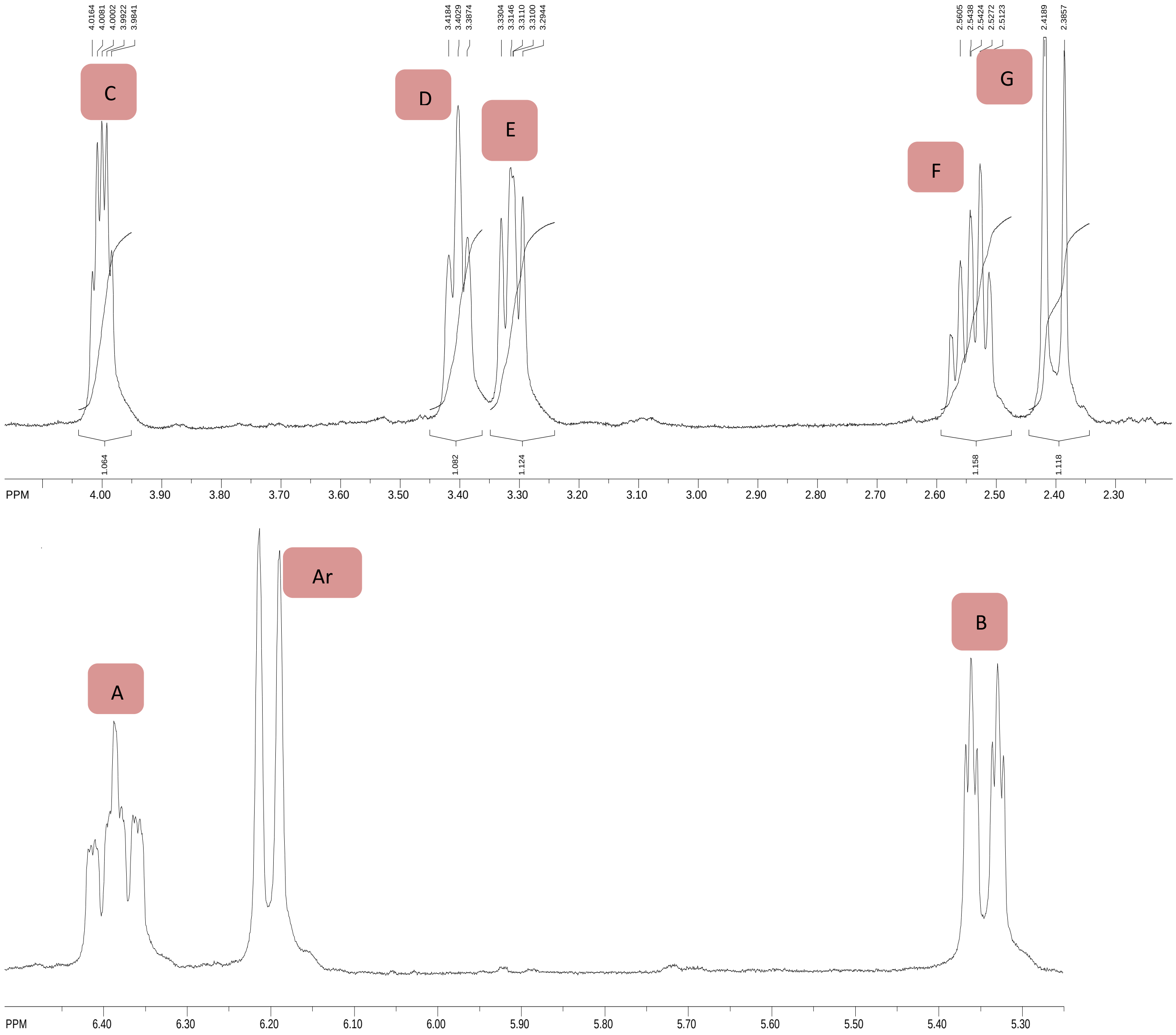

Figure S7. ^{1}H NMR spectrum ($CDCl_3$) of *endo*-**36** (previous page) and enlargement of selected parts.

11-(4-methoxyphenyl)tricyclo[6.3.1.0^{2,7}]dodeca-2,4,6,9-tetraene (*endo*-37)

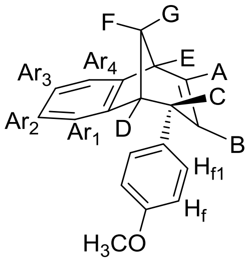

endo-**37**

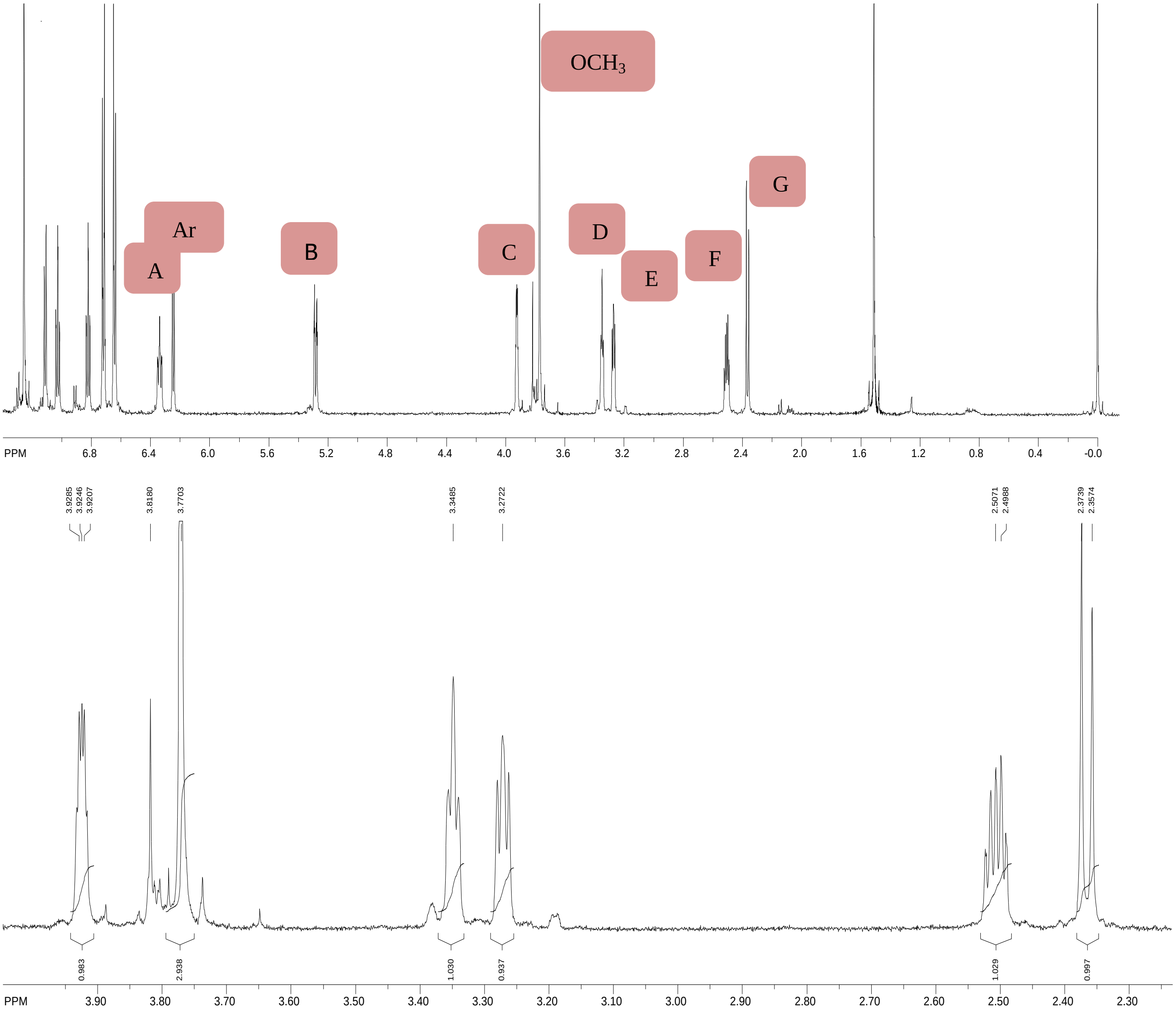

Figure S8. ^{1}H NMR spectrum ($CDCl_3$) of *endo*-**37** and enlargement of the selected part.

(2*R*,7*R*,8*S*)-8-phenyl-1a,7,8,8a-tetrahydro-2*H*-2,7-methanobenzo[4,5]cyclohepta[1,2-*b*]-oxirene (*endo*-38)

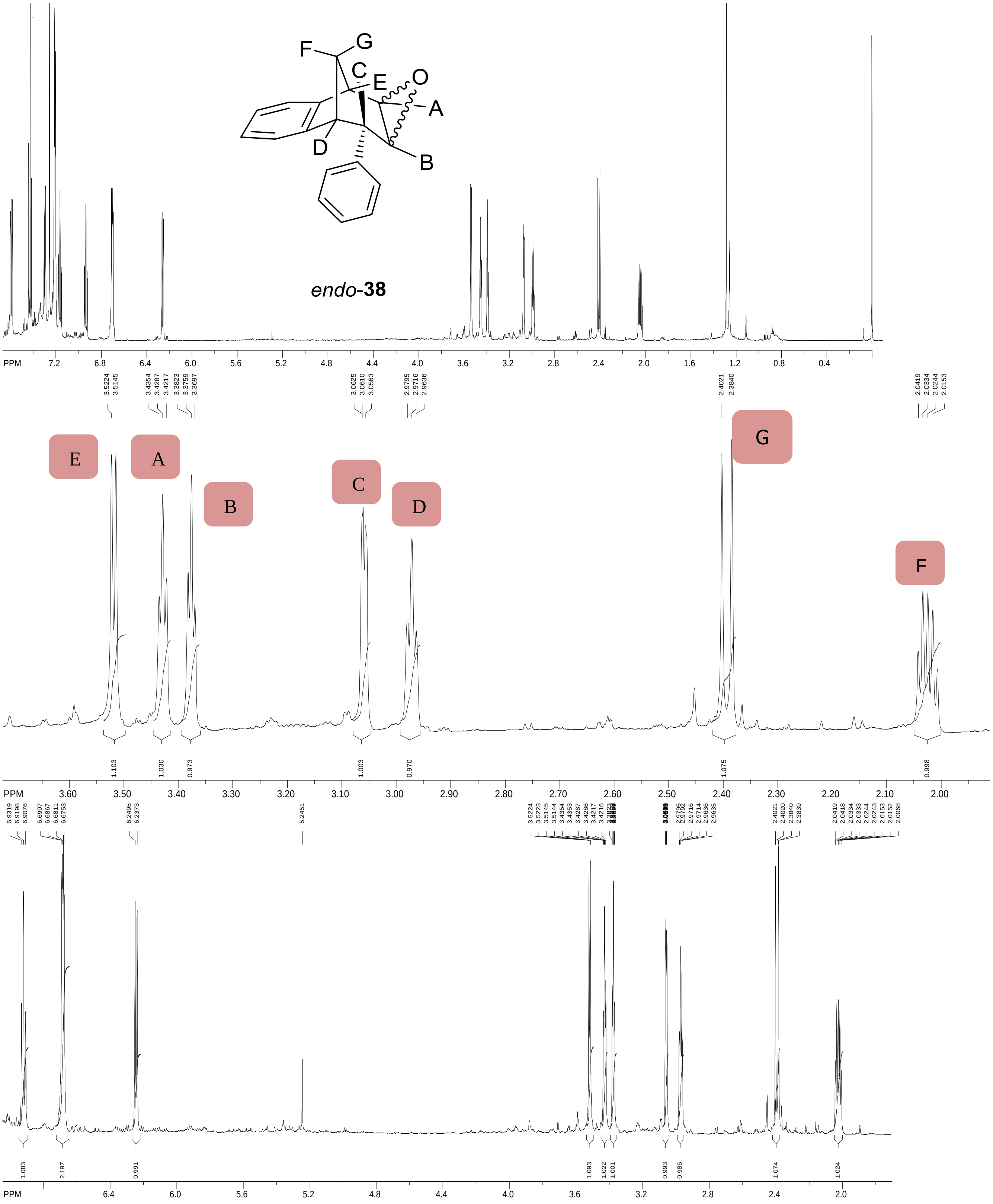

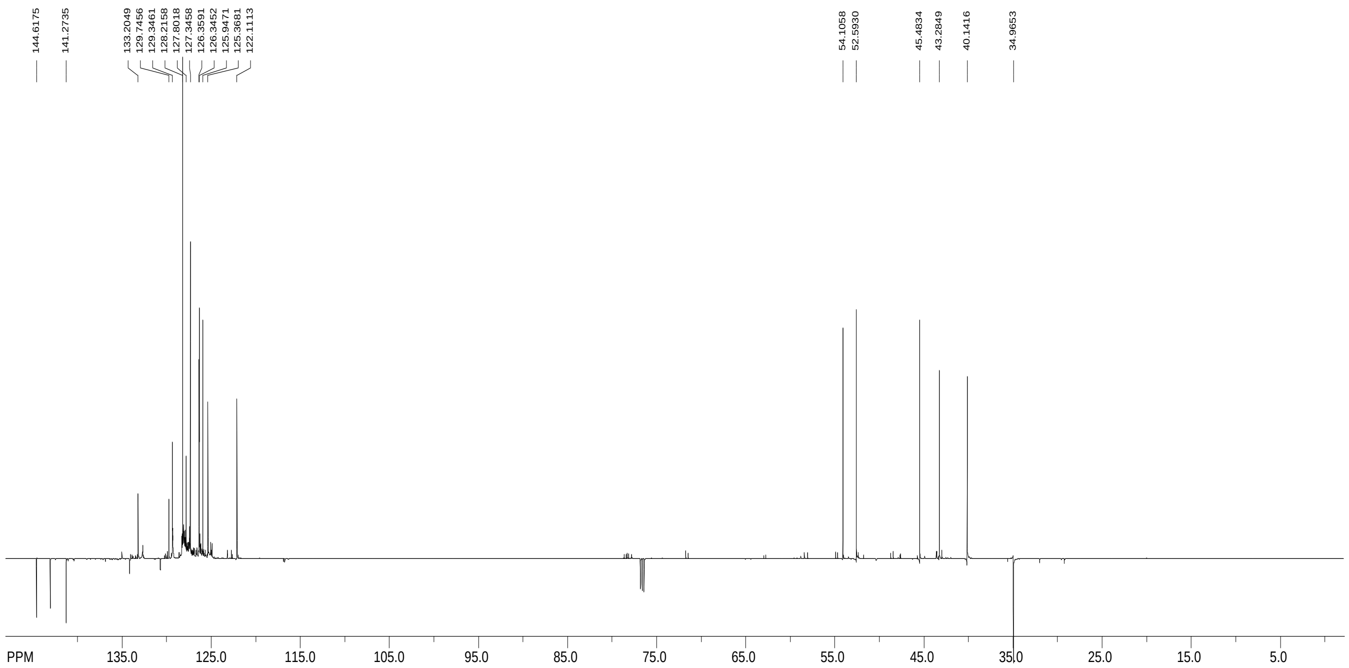

Figure S9. ^{1}H NMR spectrum ($CDCl_3$) and enlargement of selected parts (previous page) and ^{13}C NMR spectrum ($CDCl_3$) of *endo*-**38.**

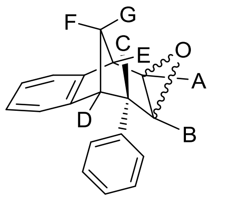

endo-**38**

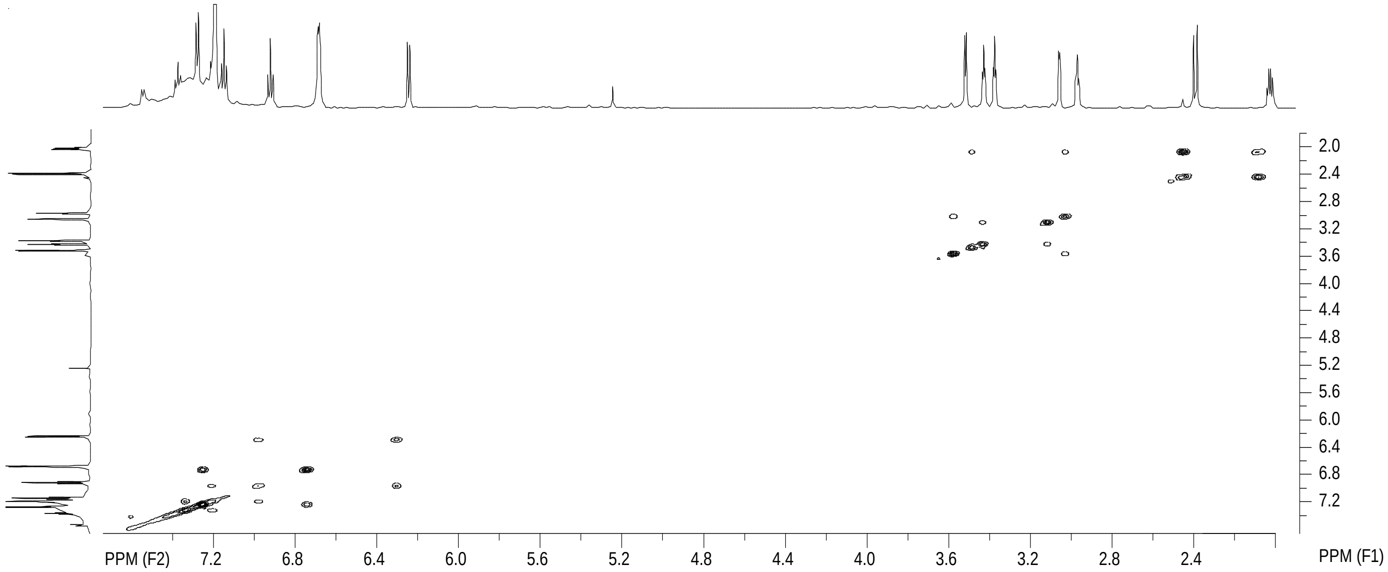

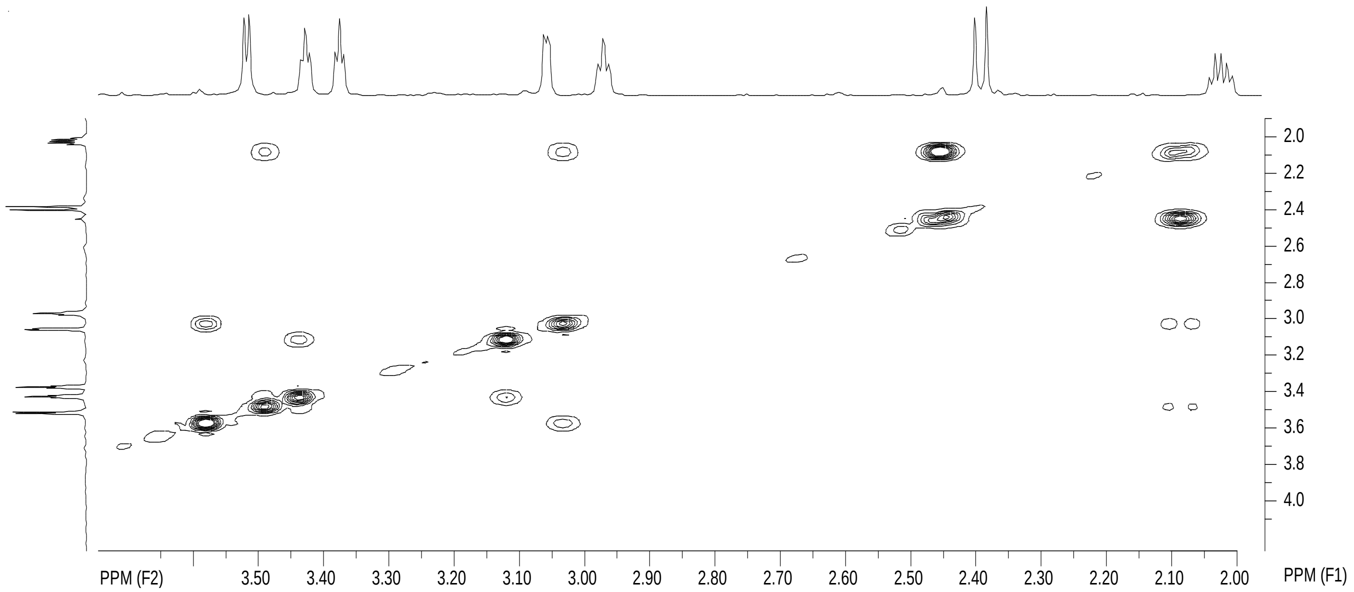

Figure S10. COSY spectrum ($CDCl_3$) and enlargement of selected part of *endo*-**38**.

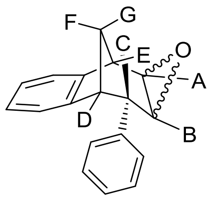

endo-**38**

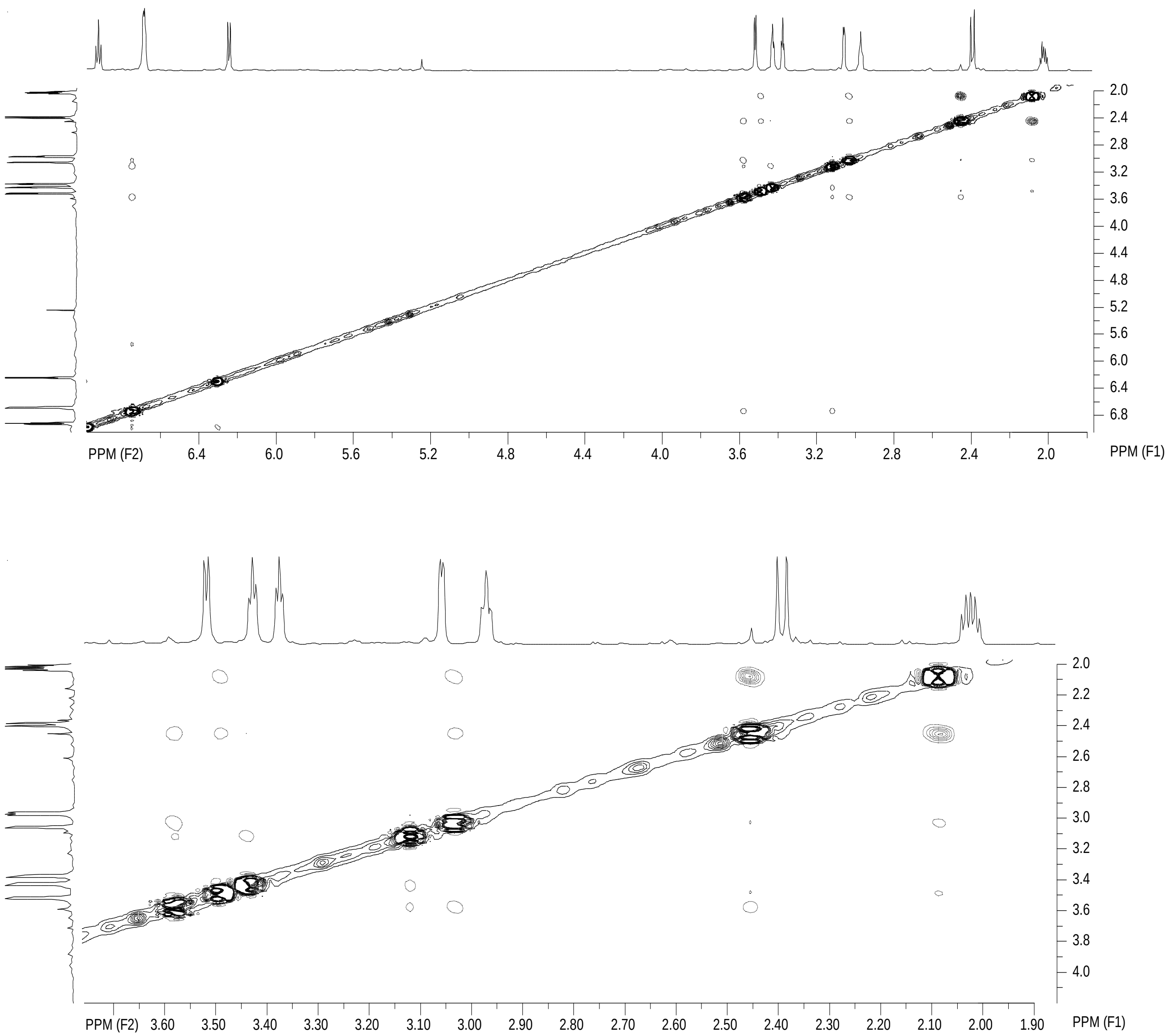

Figure S11. LR COSY spectrum ($CDCl_3$) and its aliphatic part (lower panel) of *endo*-**38.**

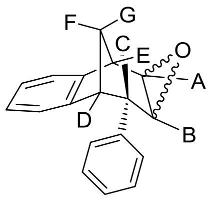

endo-**38**

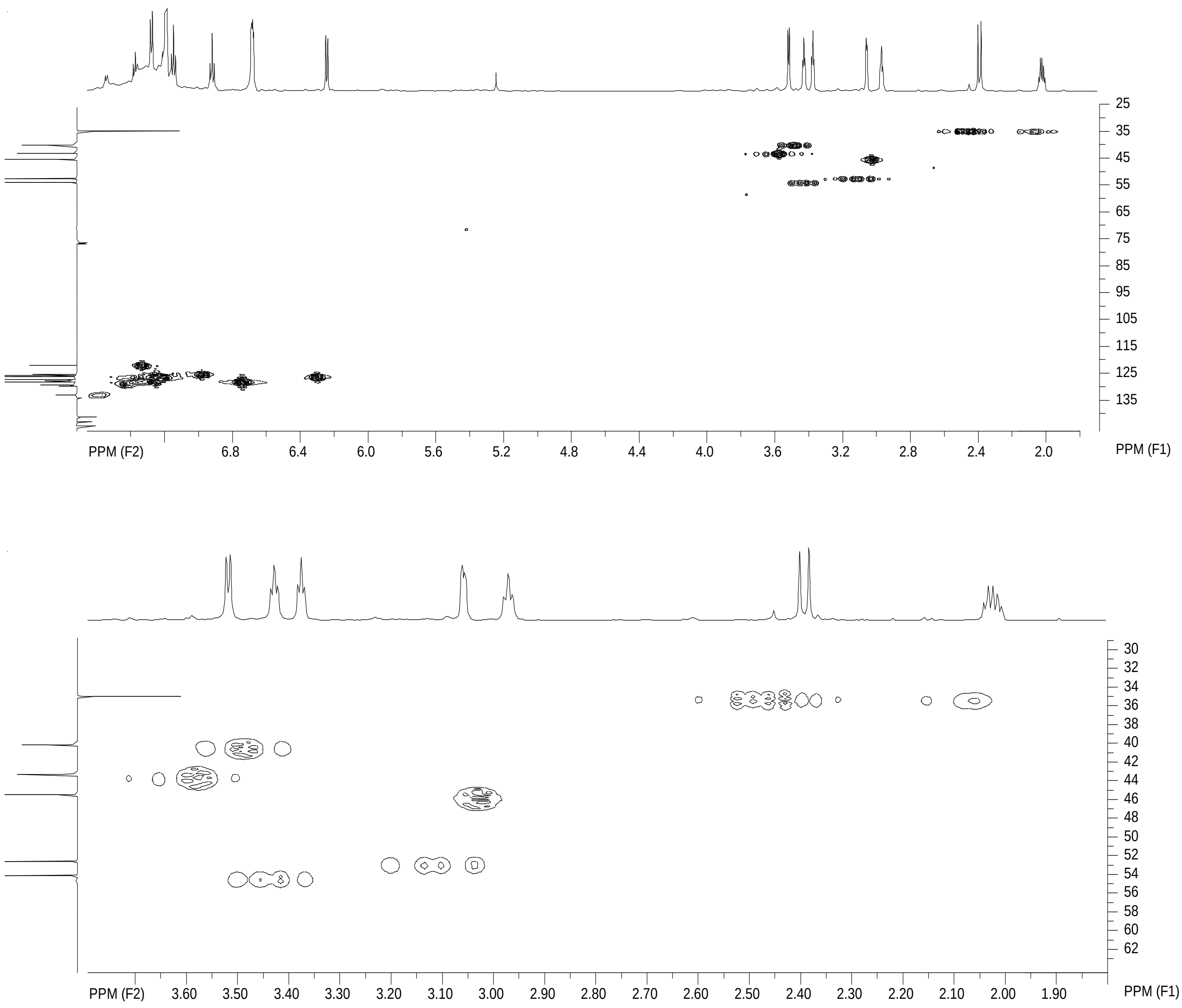

Figure S12. HSQC spectrum ($CDCl_3$) and enlargement of the selected part of *endo*-**38**.

(2*R*,7*R*,8*S*,8a*S*)-8-(4-methoxyphenyl)-1a,7,8,8a-tetrahydro-2*H*-2,7-methanobenzo[4,5]cyclohepta[1,2-*b*]oxirene (*endo*-39)

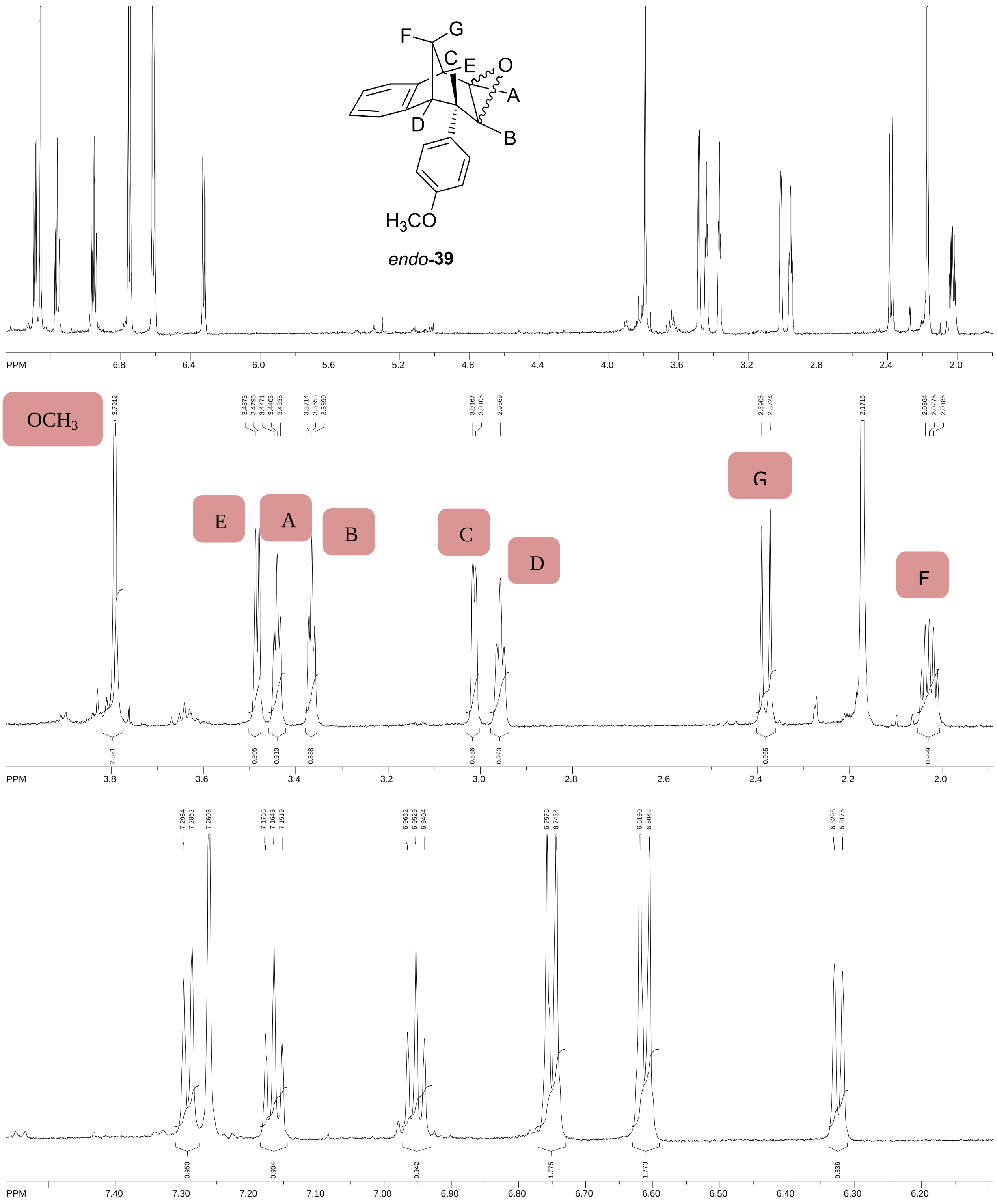

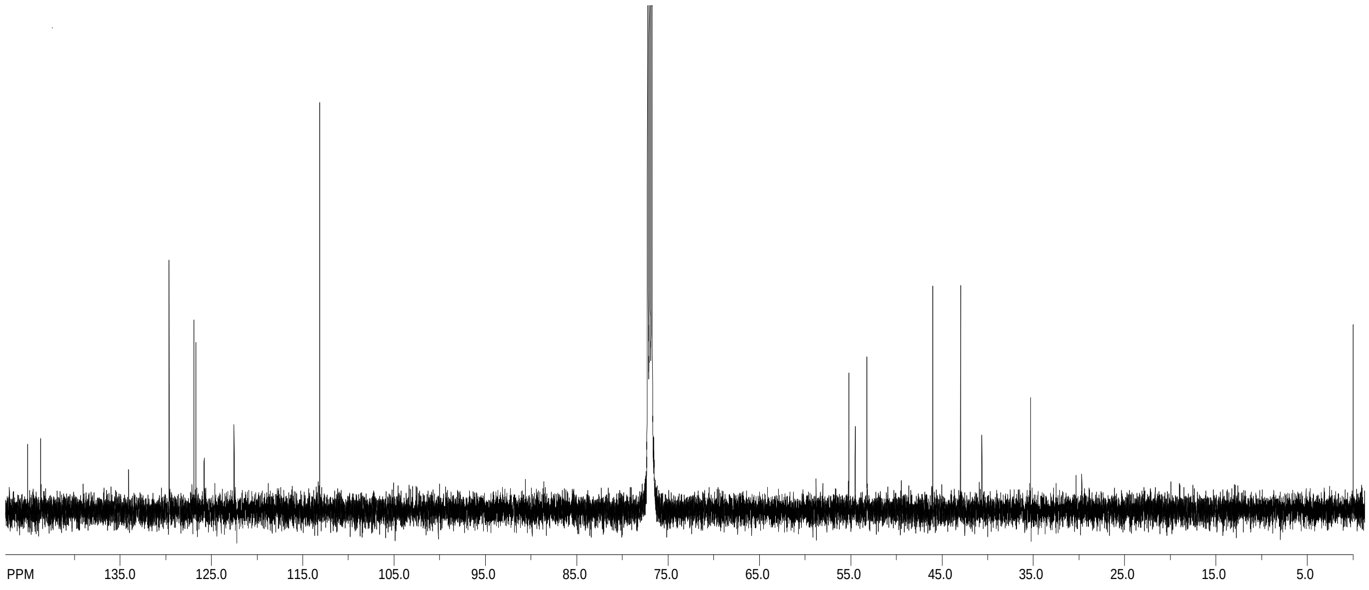

Figure S13. ^{1}H NMR spectrum ($CDCl_3$) and its aliphatic and aromatic part (previous page) and ^{13}C NMR spectrum ($CDCl_3$) of *endo*-**39.**

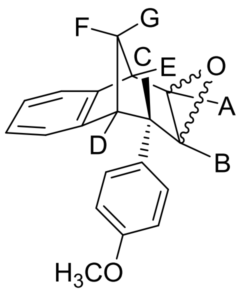

endo-**39**

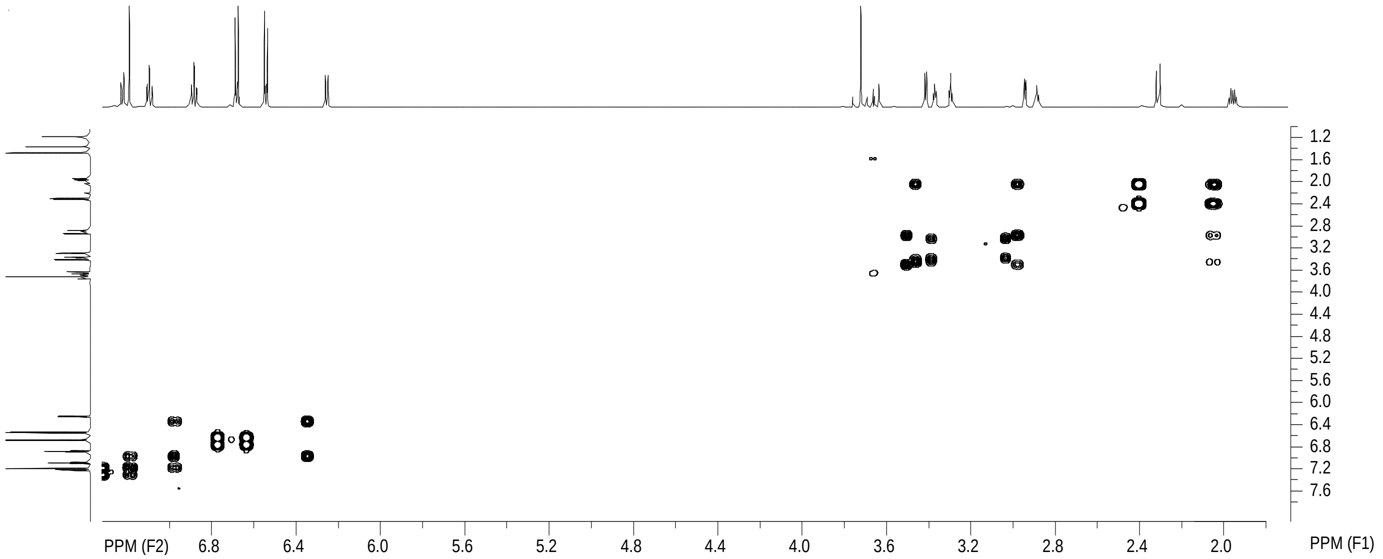

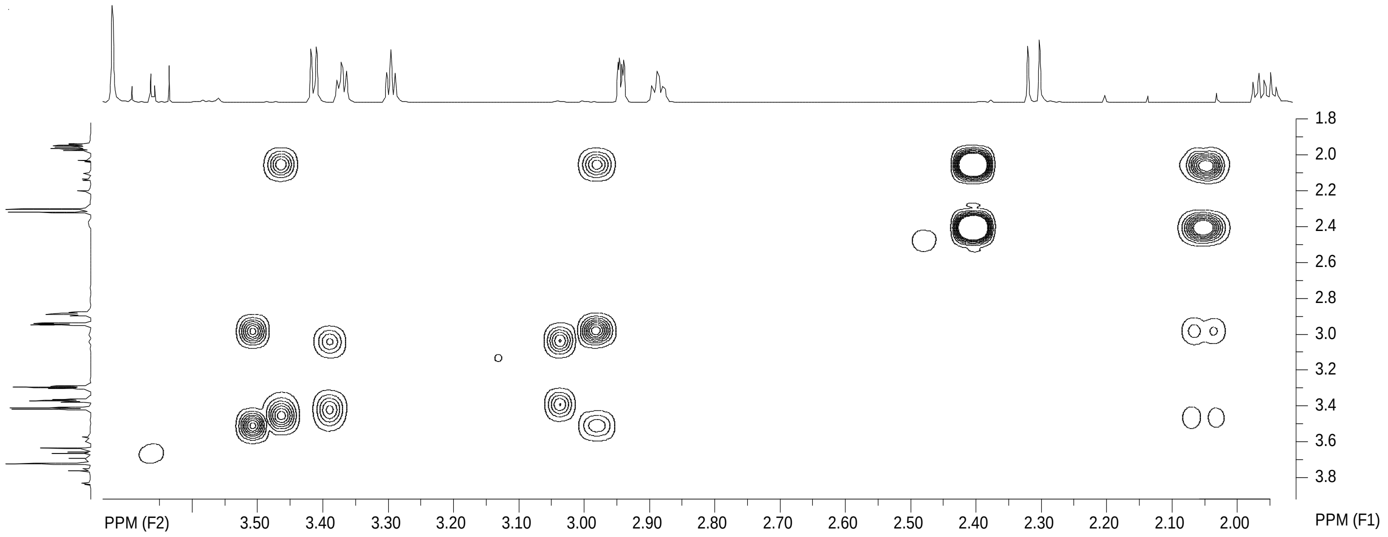

Figure S14. COSY spectrum (CDCl3) and enlargement of the aliphatic part of *endo*-**39.**

(6*S*)-6-phenyl-6,7,8,9-tetrahydro-5*H*-5,9-methanobenzo[7]anulen-7-ol (*endo*-40)

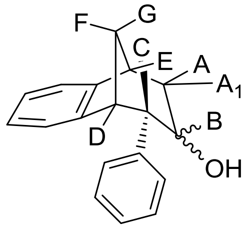

endo-**40**

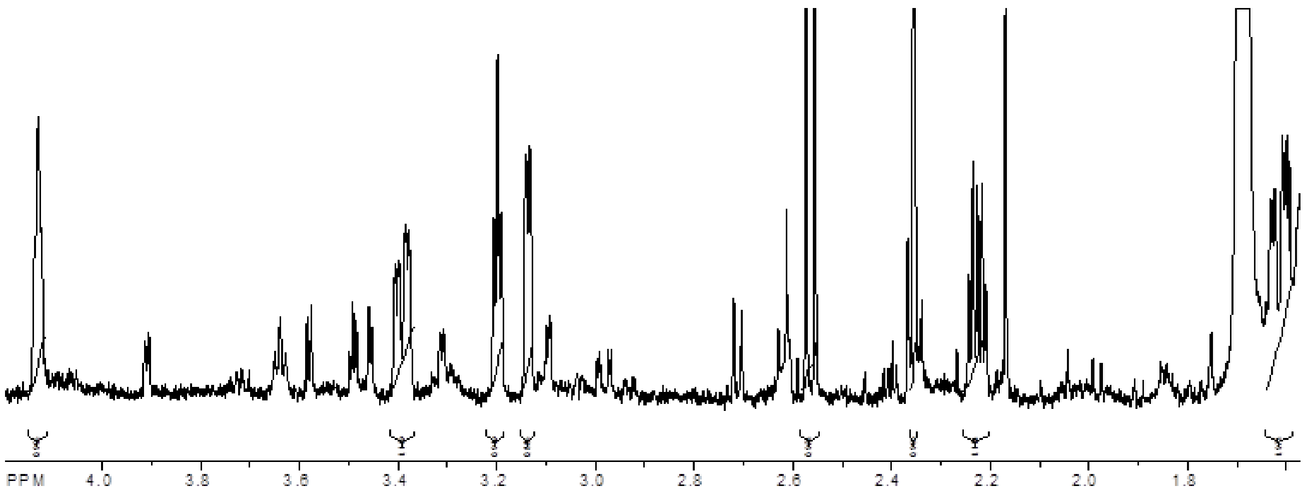

Figure S15. ^{1}H NMR spectrum ($CDCl_3$) of alcohol *endo*-**40** (there is one main alcohol product and at least one minor stereoisomer).

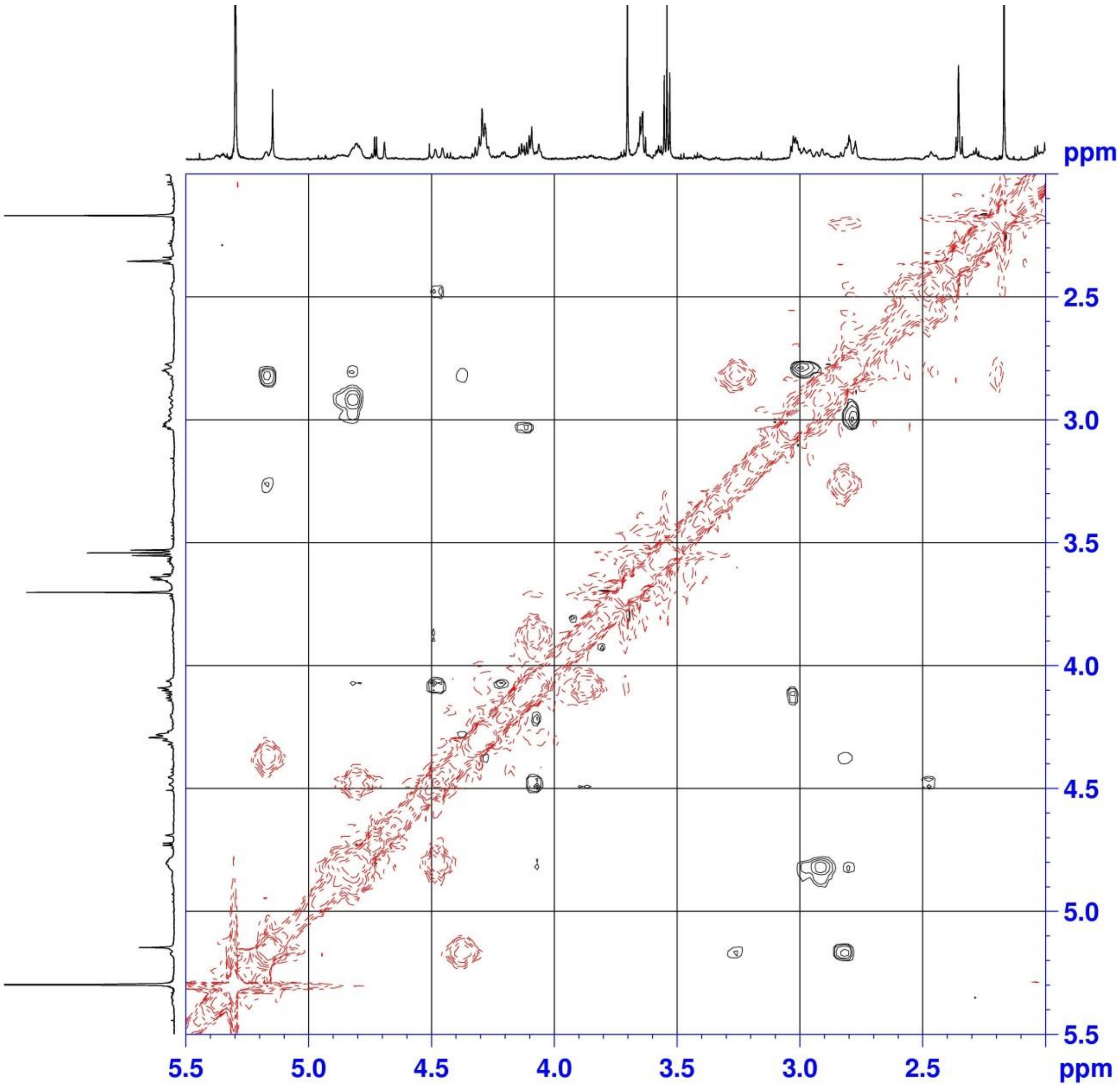

Figure S16. NOESY spectrum ($CDCl_3$) of alcohol *endo*-**40** (there is one main alcohol product and at least one minor stereoisomer).

((5*R*,6*S*,9*S*)-6-phenyl-7-propoxy-6,7,8,9-tetrahydro-5*H*-5,9-methanobenzo[7]annulene (*endo*-43)

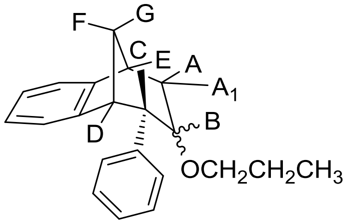

endo-**43**

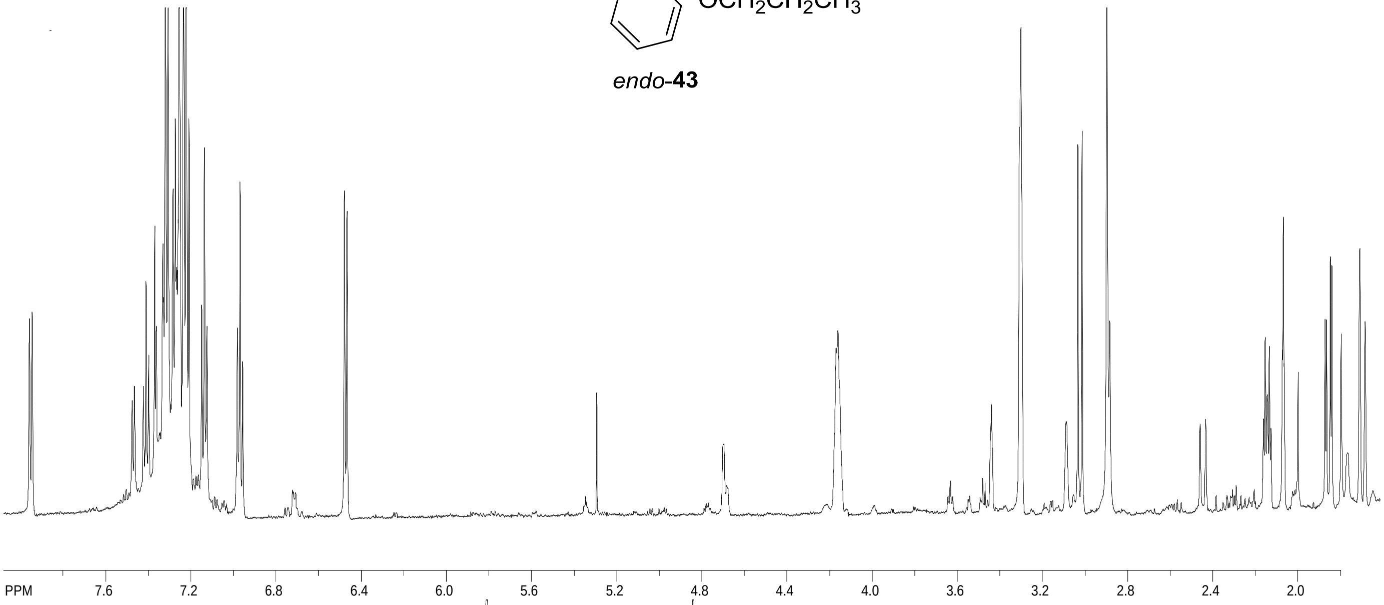

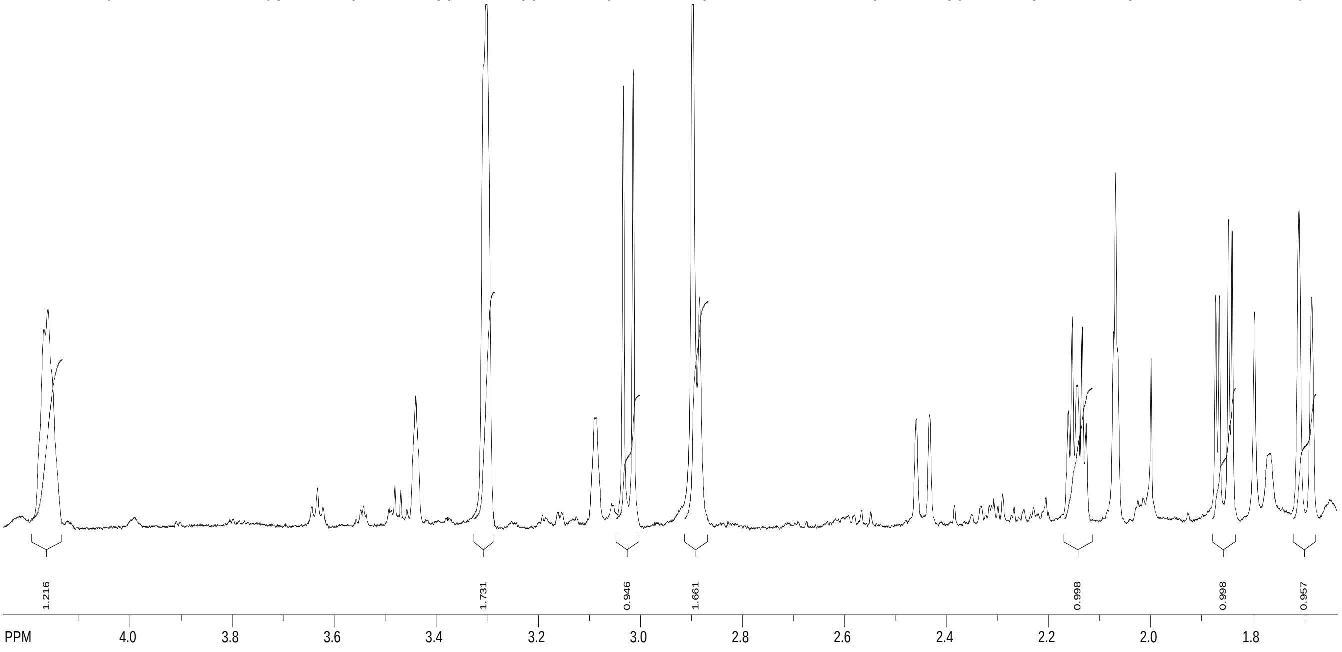

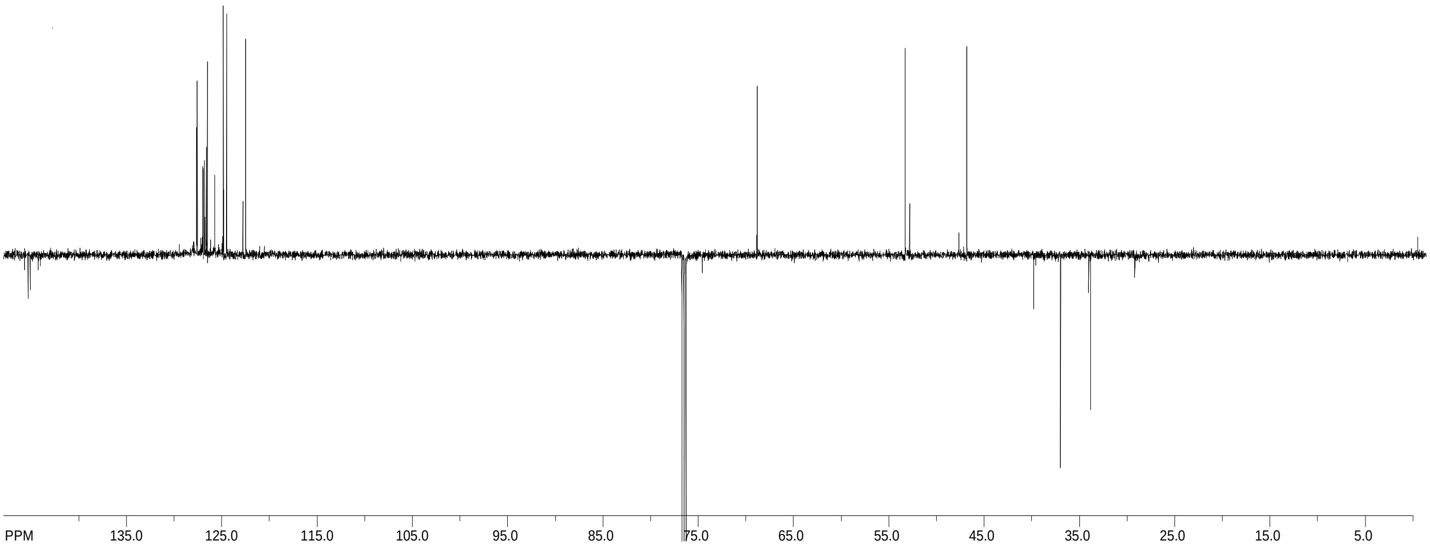

Figure S17.^{1}H NMR spectrum (CDCl3) and its aliphatic part (previous page), and ^{13}C NMR spectrum (CDCl3) of *endo*-**43.**

(5*R*,6*S*,9*S*)-7-isopropoxy-6-phenyl-6,7,8,9-tetrahydro-5*H*-5,9-methanobenzo[7]annulene (*endo*-44)

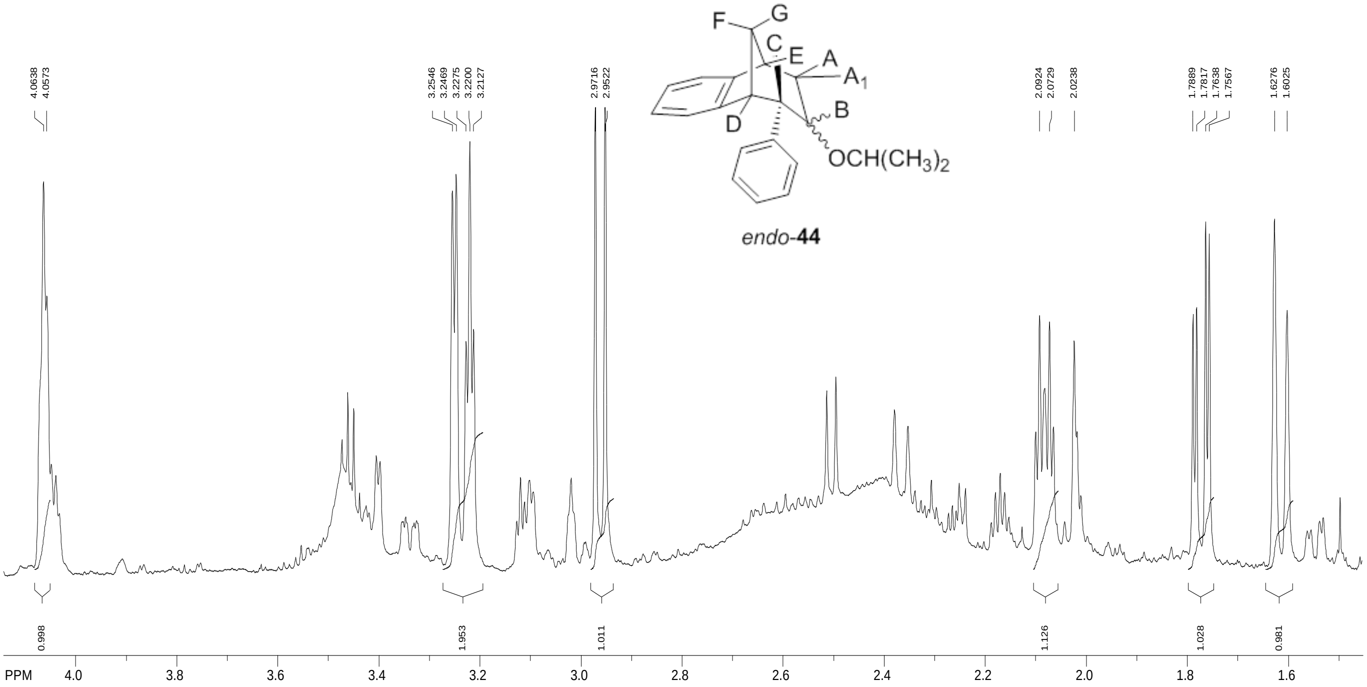

Figure S18. Aliphatic part of the ^{1}H NMR spectrum (CDCl3) of *endo*-**44.**

3-(((5*R*,6*S*,9*S*)-6-phenyl-6,7,8,9-tetrahydro-5*H*-5,9-methanobenzo[7]annulen-7-il)oxy)propan-1-ol (*endo*-45)

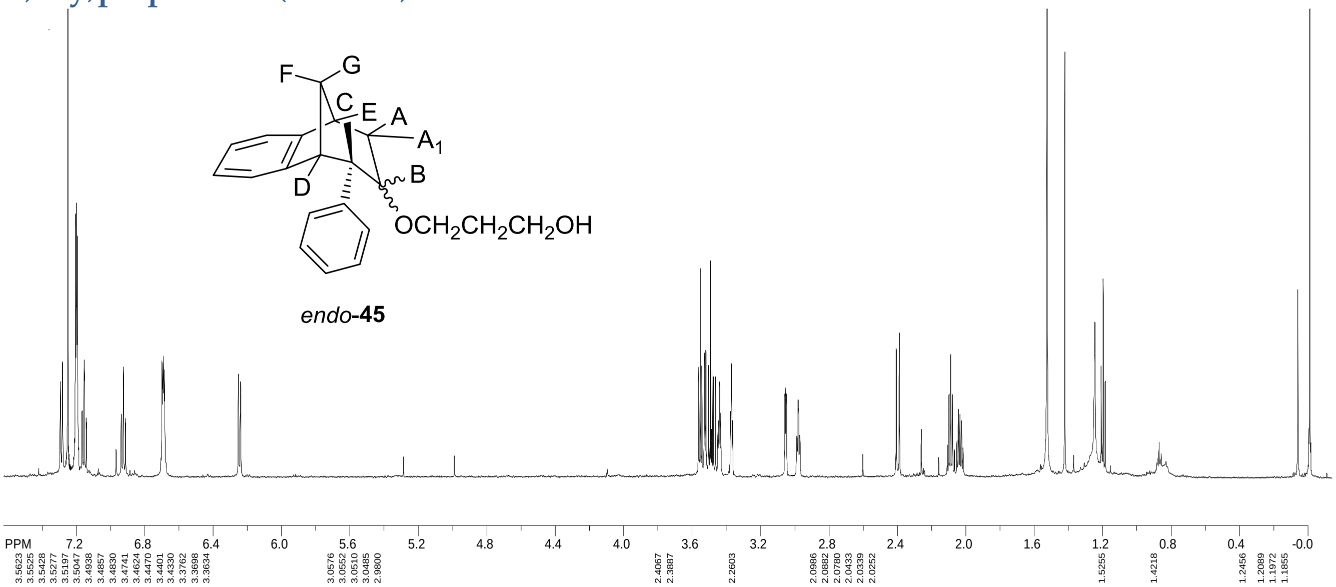

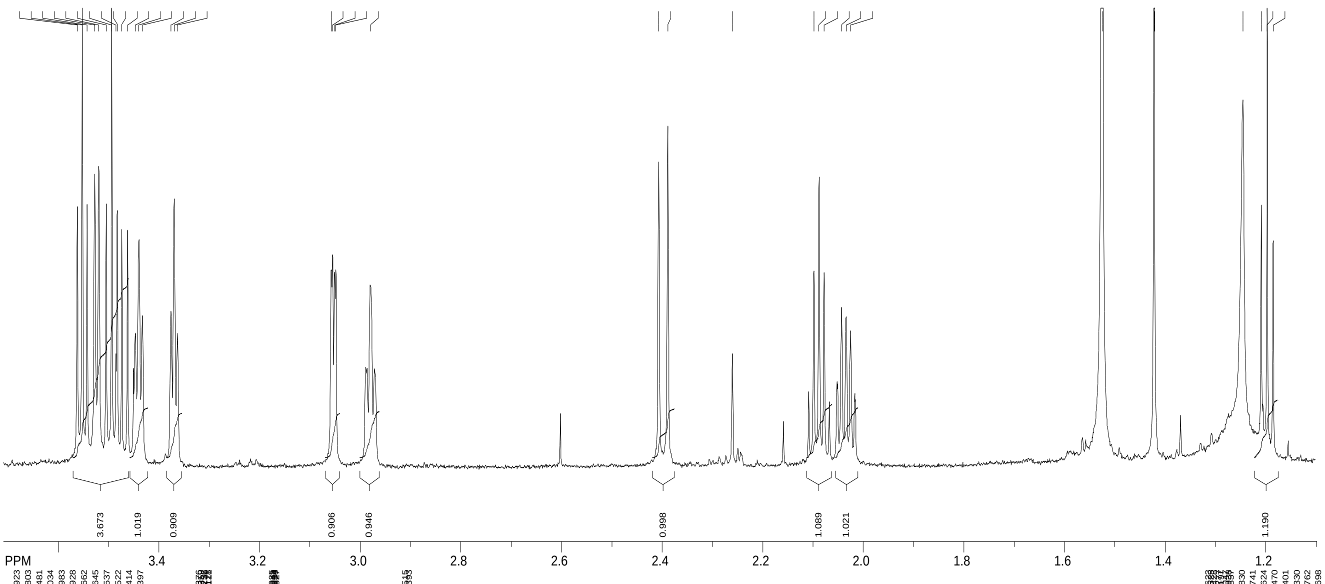

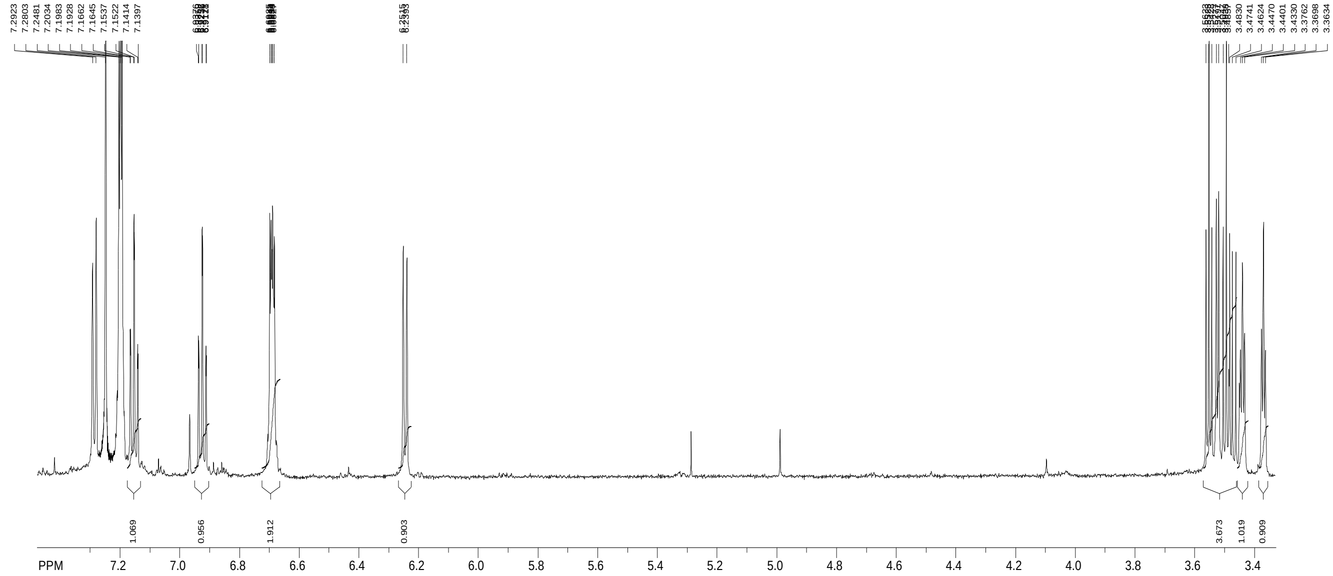

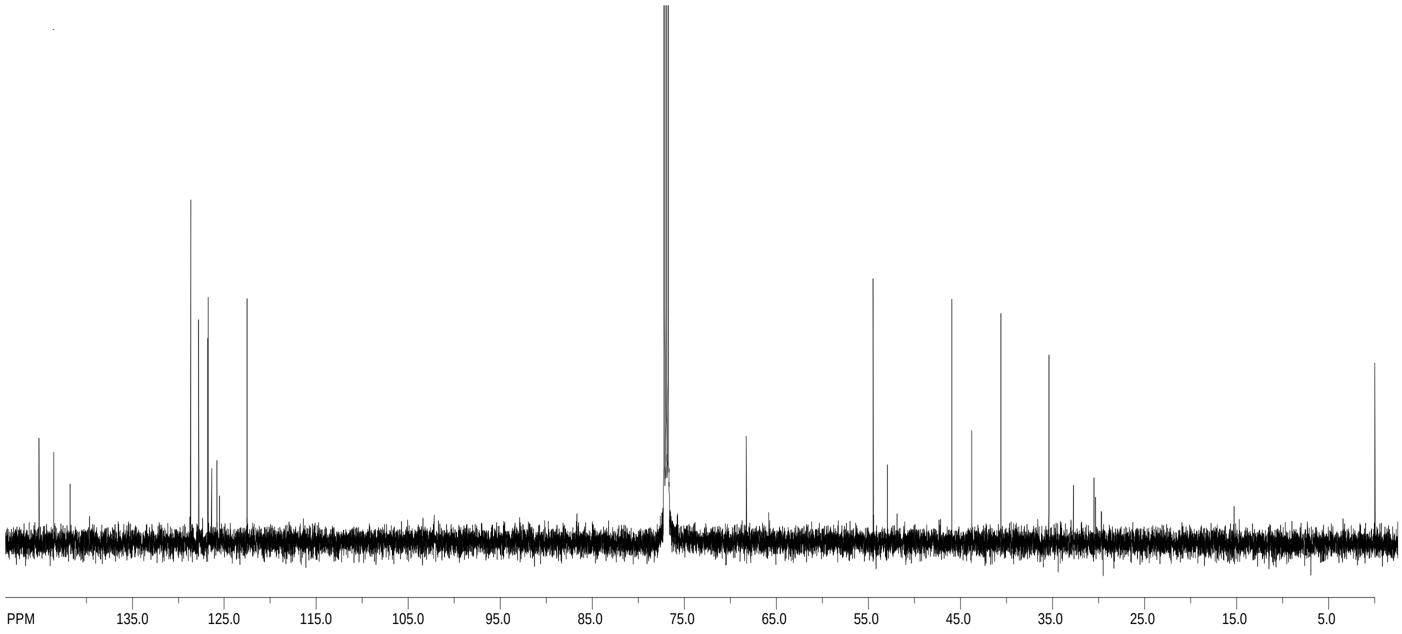

Figure S19. ^{1}H NMR spectrum ($CDCl_3$), its aliphatic and enlarged selected part (previous page), and ^{13}C NMR spectrum ($CDCl_3$) of *endo*-**45**

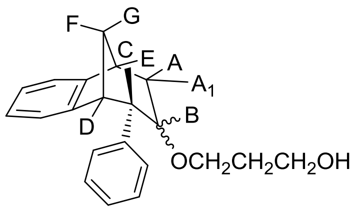

endo-**45**

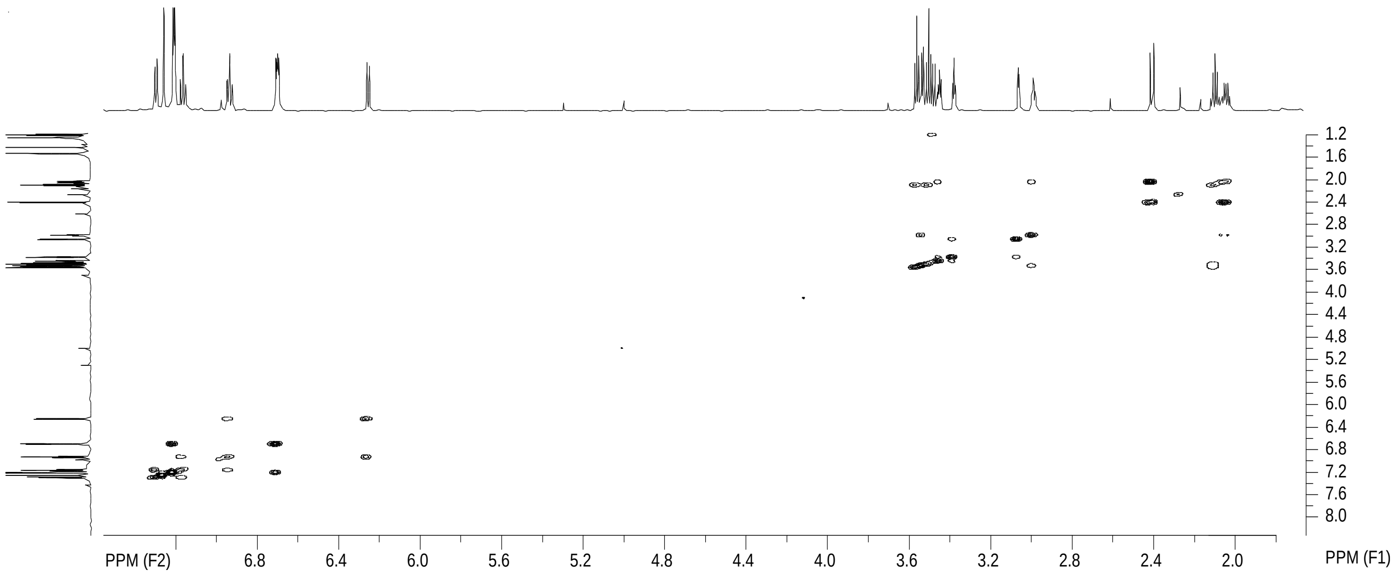

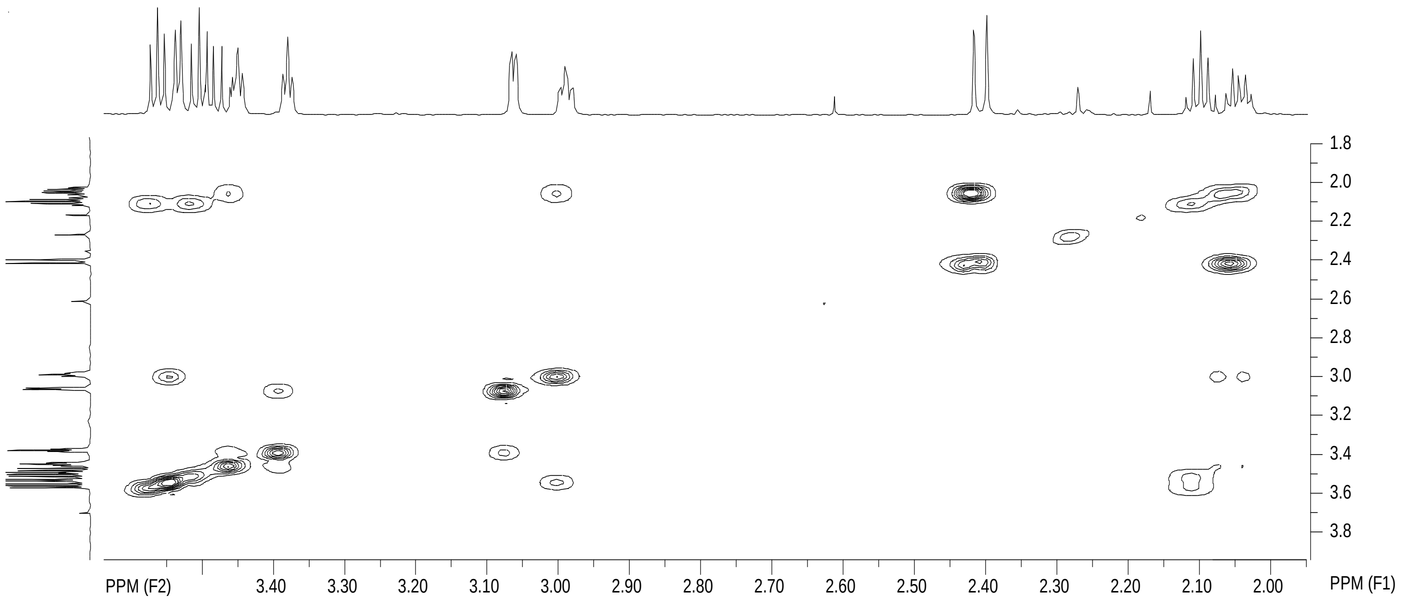

Figure S20. COSY spectrum (CDCl3) and aliphatic part of the COSY spectrum (CDCl3) of *endo*-**45.**

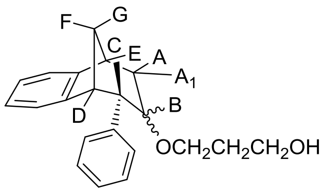

endo-**45**

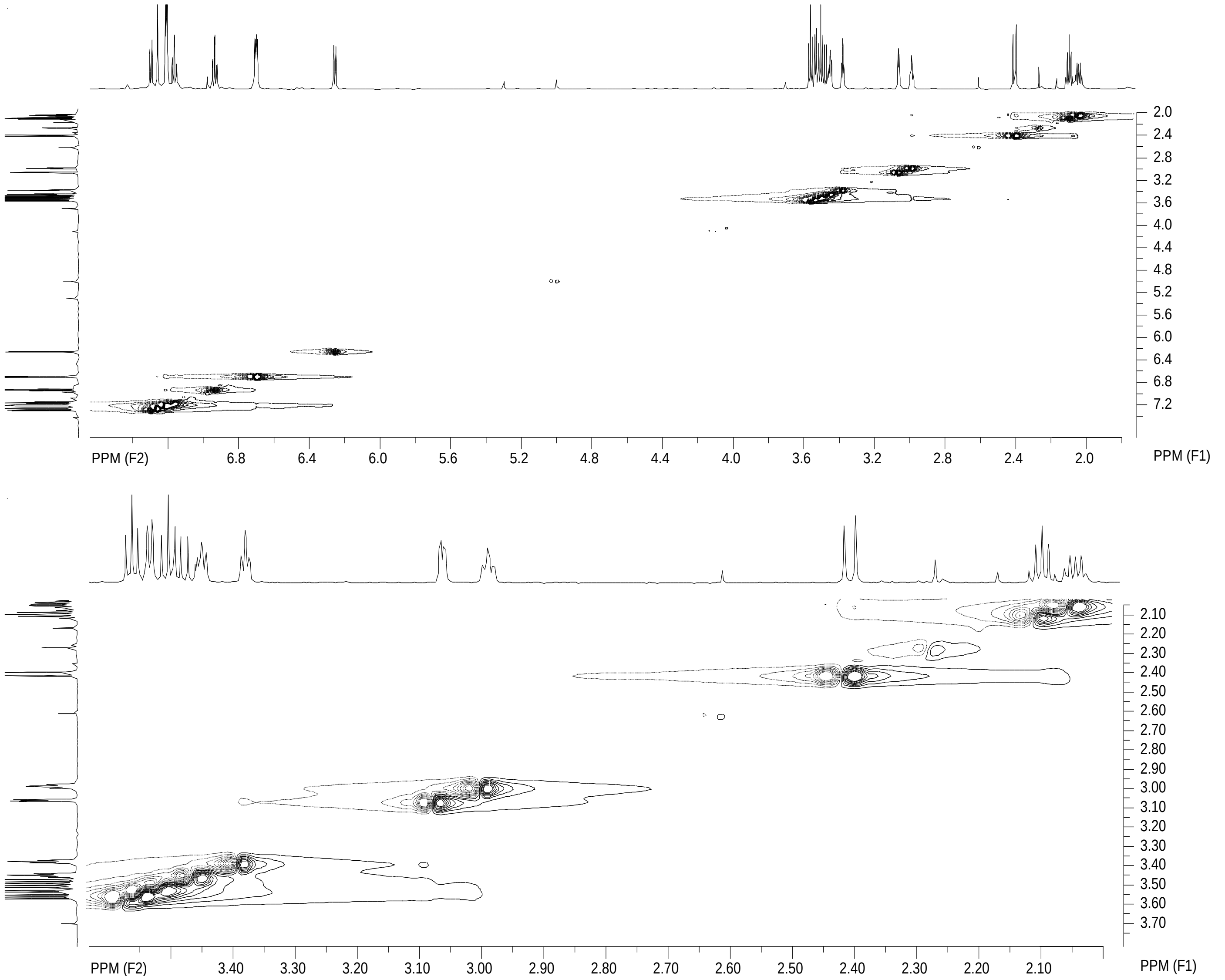

Figure S21. LR COSY spectrum ($CDCl_3$) and enlarged selected part of *endo*-**45.**

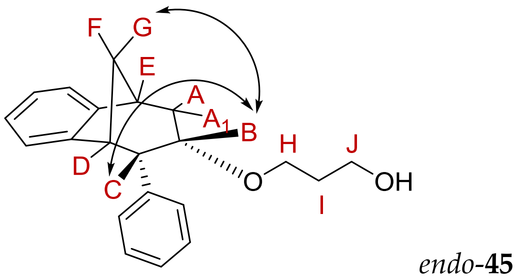

endo-**45**

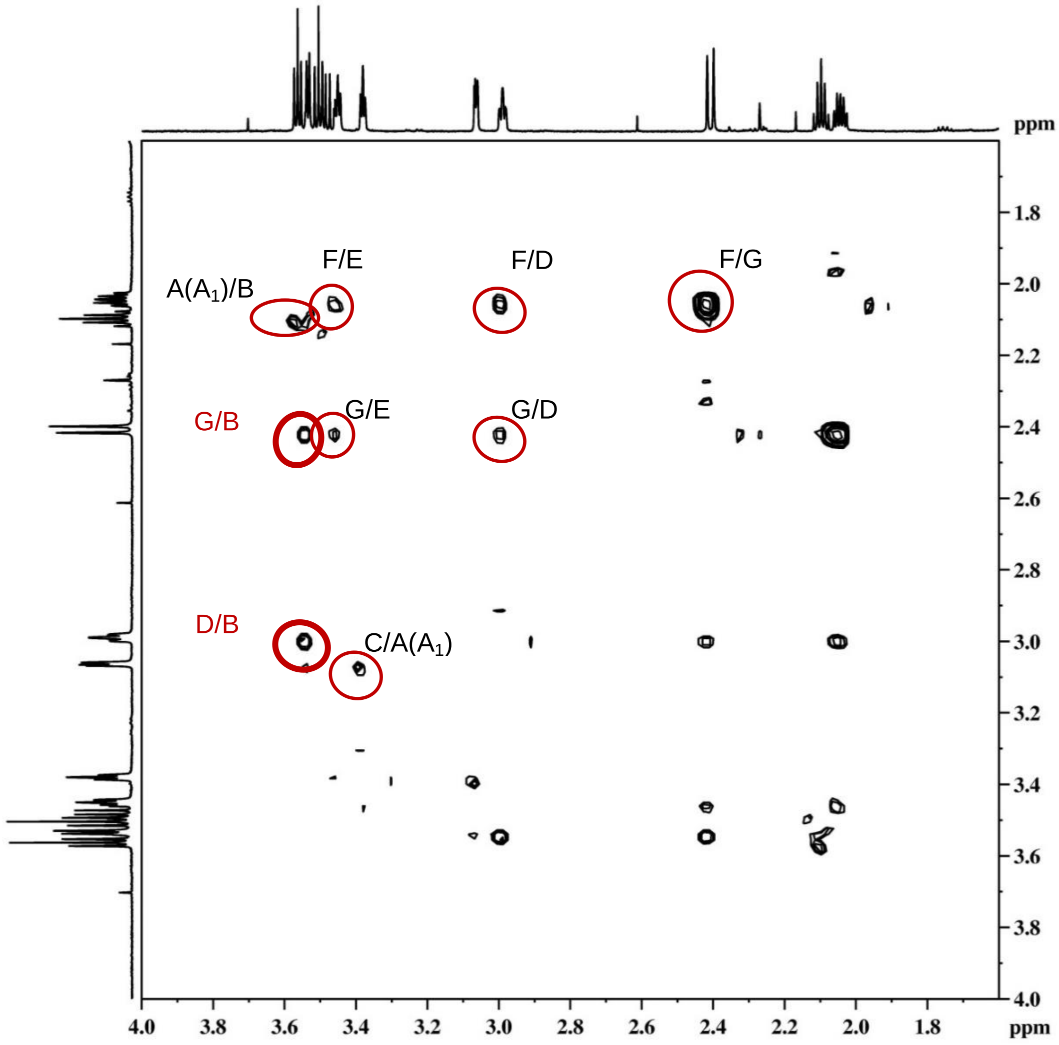

Figure S22. NOESY spectrum ($CDCl_3$) of *endo*-**45.**

(5*R*,6*S*,9*S*)-7-ethoxy-6-(4-methoxyphenyl)-6,7,8,9-tetrahydro-5*H*-5,9-methanobenzo-[7]annulene (*endo*-46)

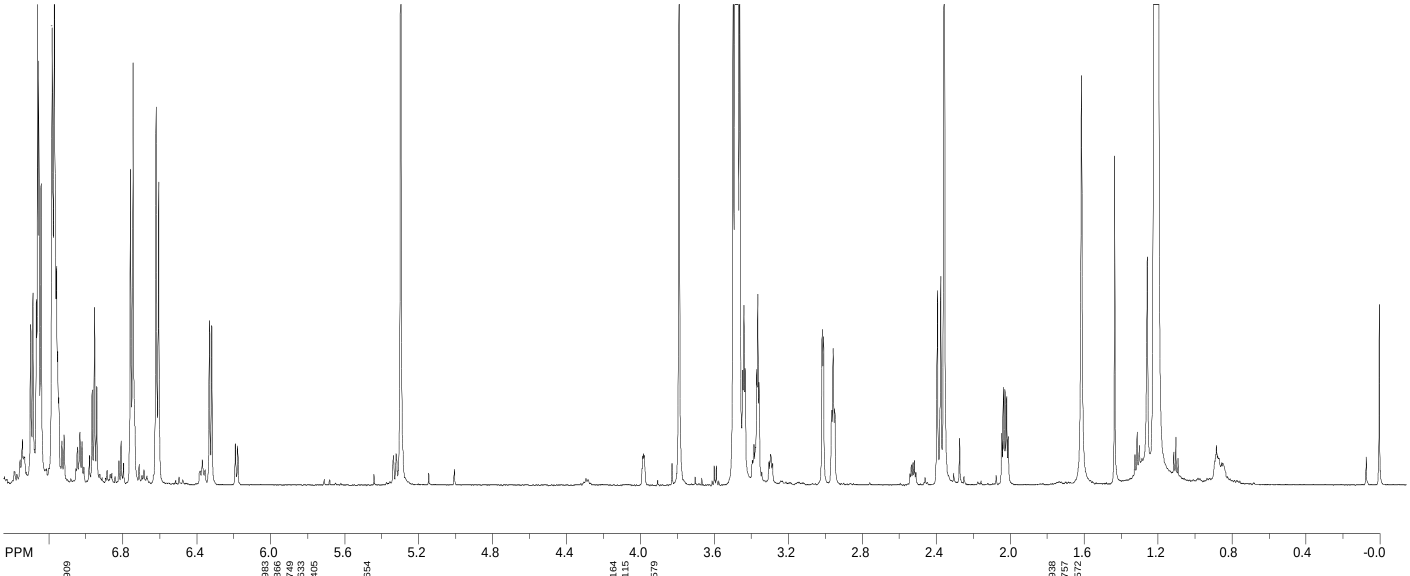

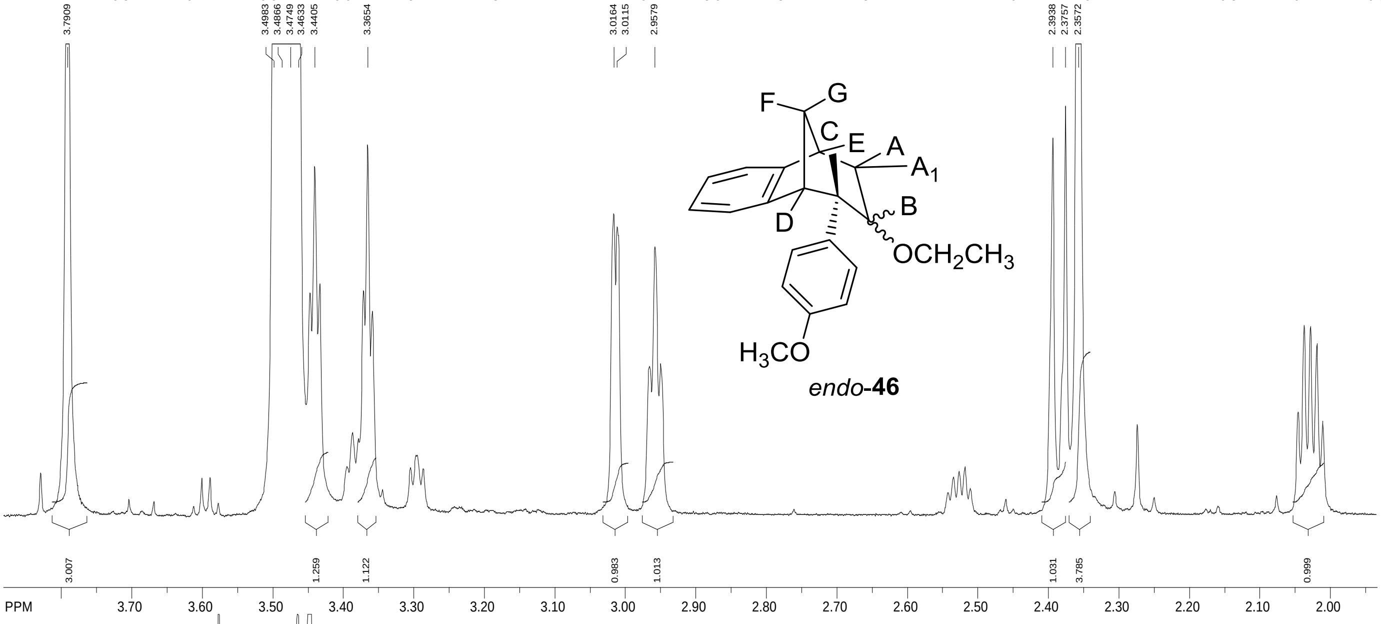

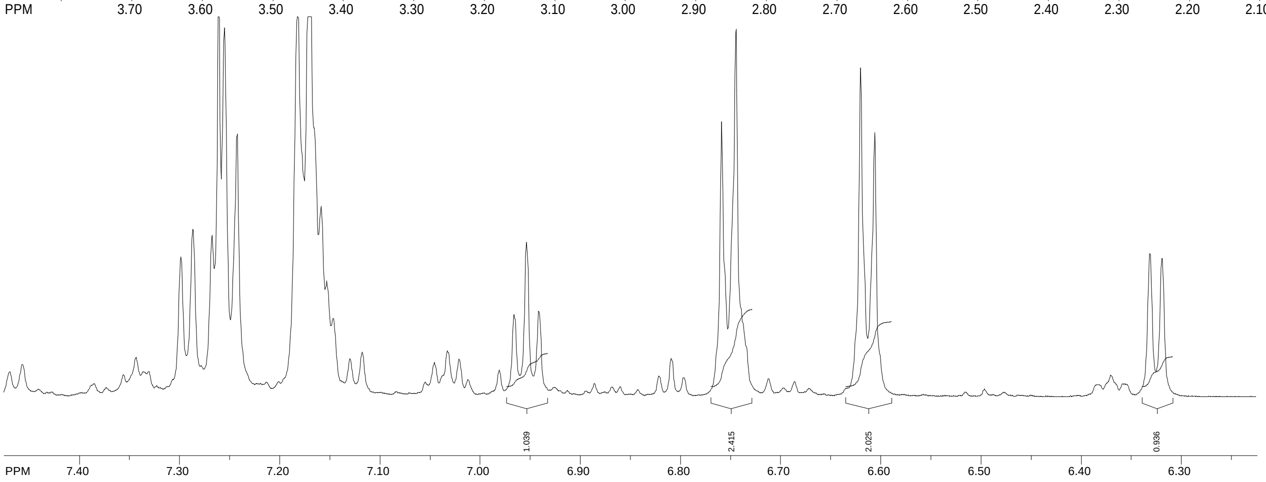

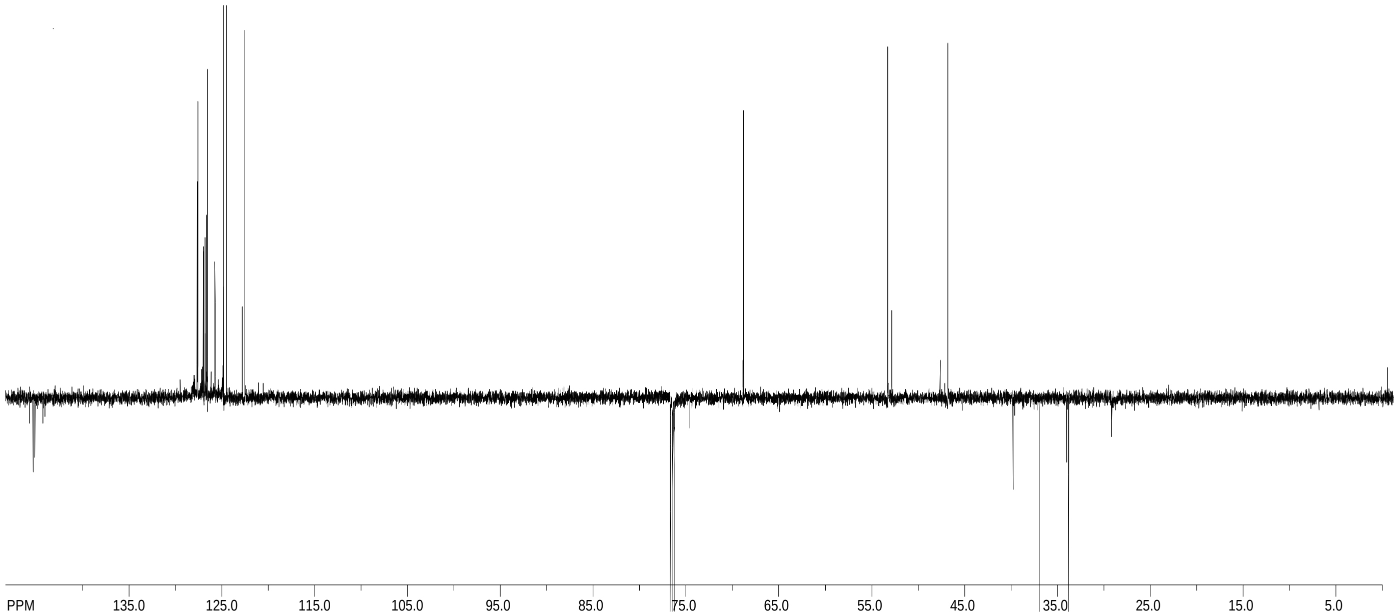

Figure S23. ^{1}H NMR spectrum ($CDCl_3$) and its aliphatic and aromatic parts (previous page) and ^{13}C NMR spectrum ($CDCl_3$) of *endo*-**46.**

(5*R*,6*S*,9*S*)-6-(4-methoxyphenyl)-7-propoxy-6,7,8,9-tetrahydro-5*H*-5,9-methanobenzo[7] anunlene (*endo*-47)

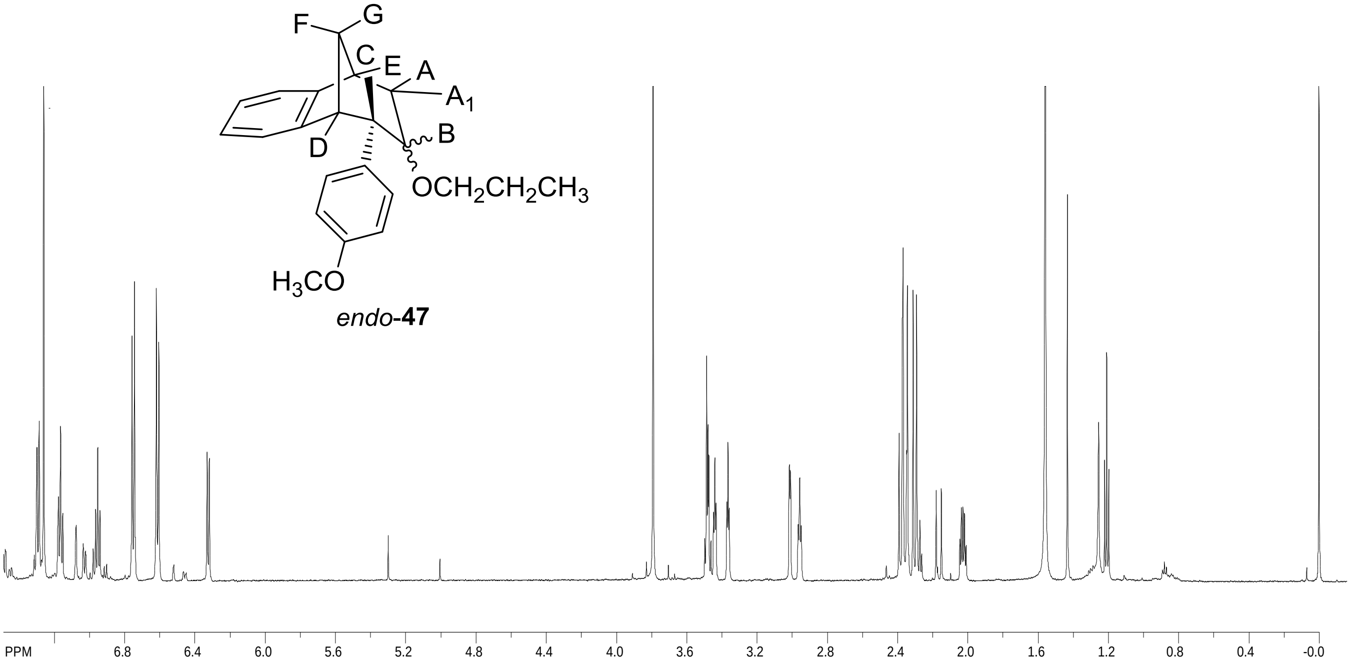

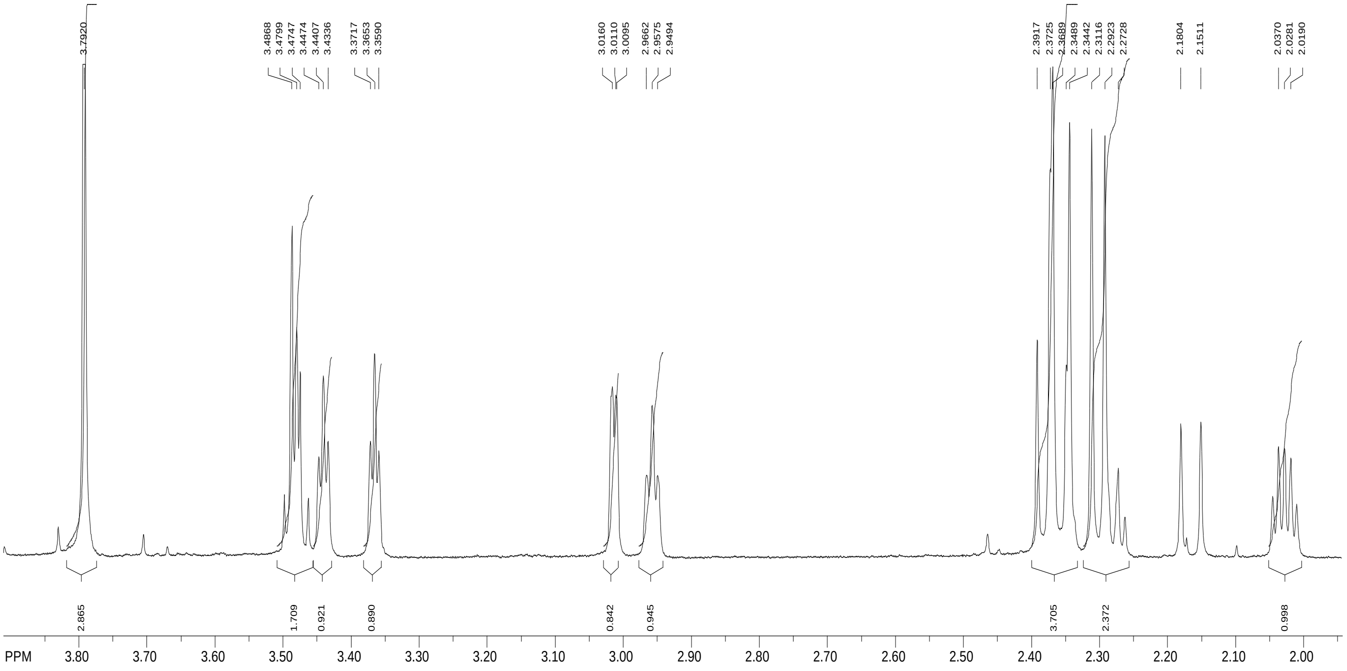

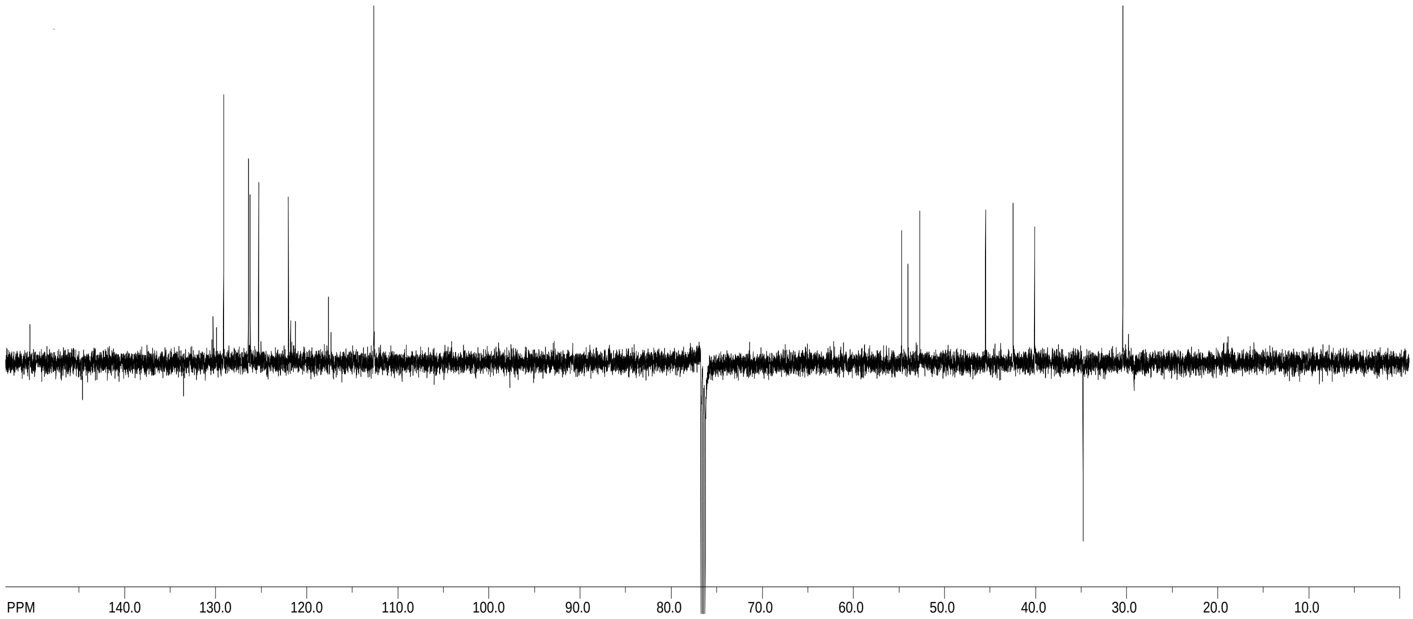

Figure S24. ^{1}H NMR spectrum ($CDCl_3$) and its enlarged selected part(previous page) and ^{13}C NMR spectrum ($CDCl_3$) of *endo*-**47.**

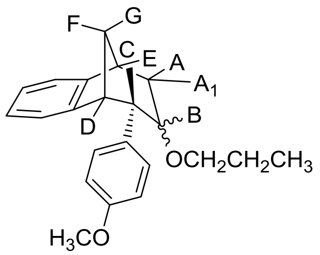

endo-**47**

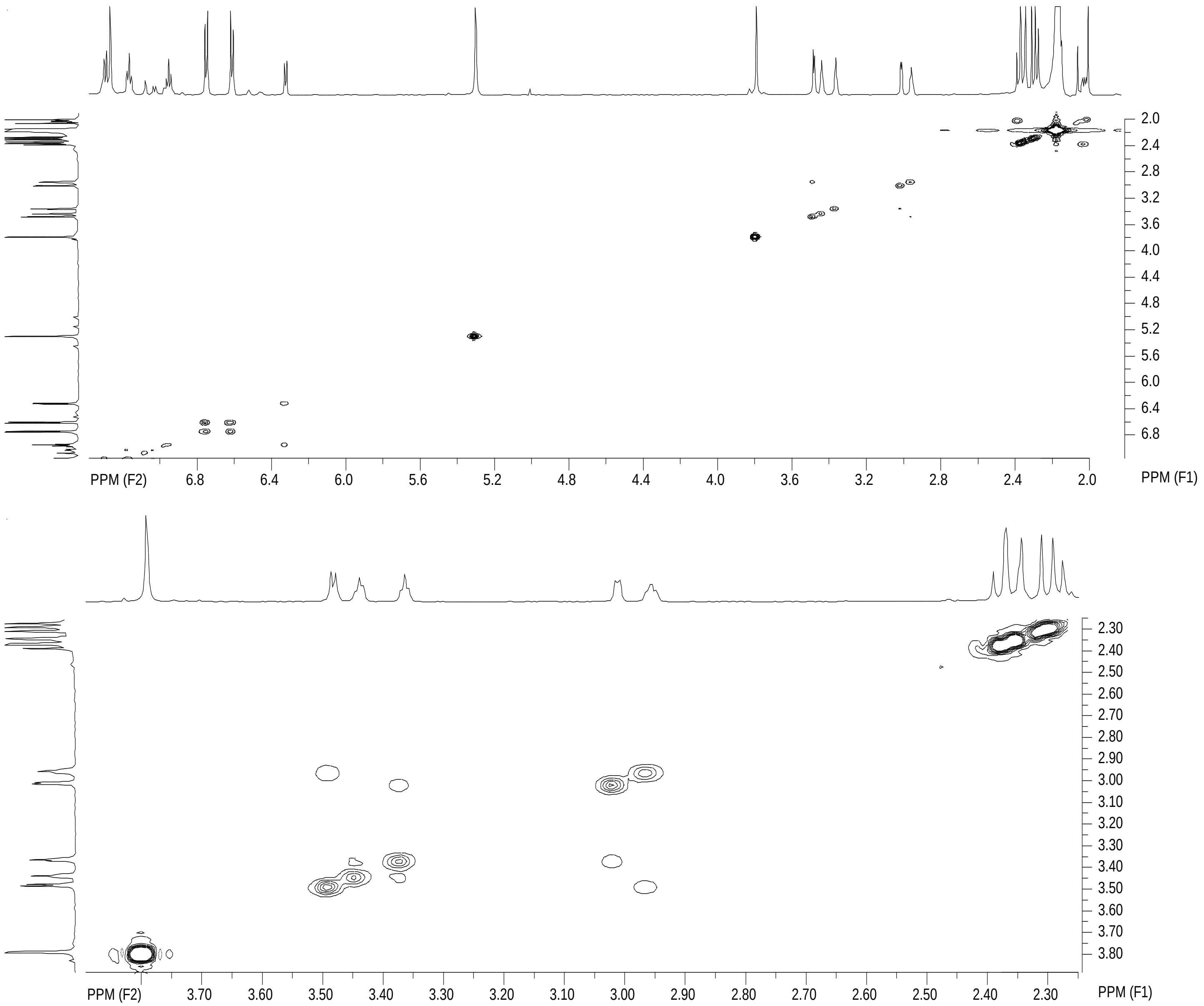

Figure S25. COSY spectrum ($CDCl_3$) of *endo*-**47**and its aliphatic part.

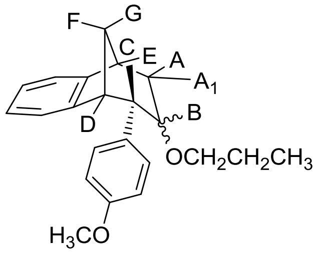

endo-**47**

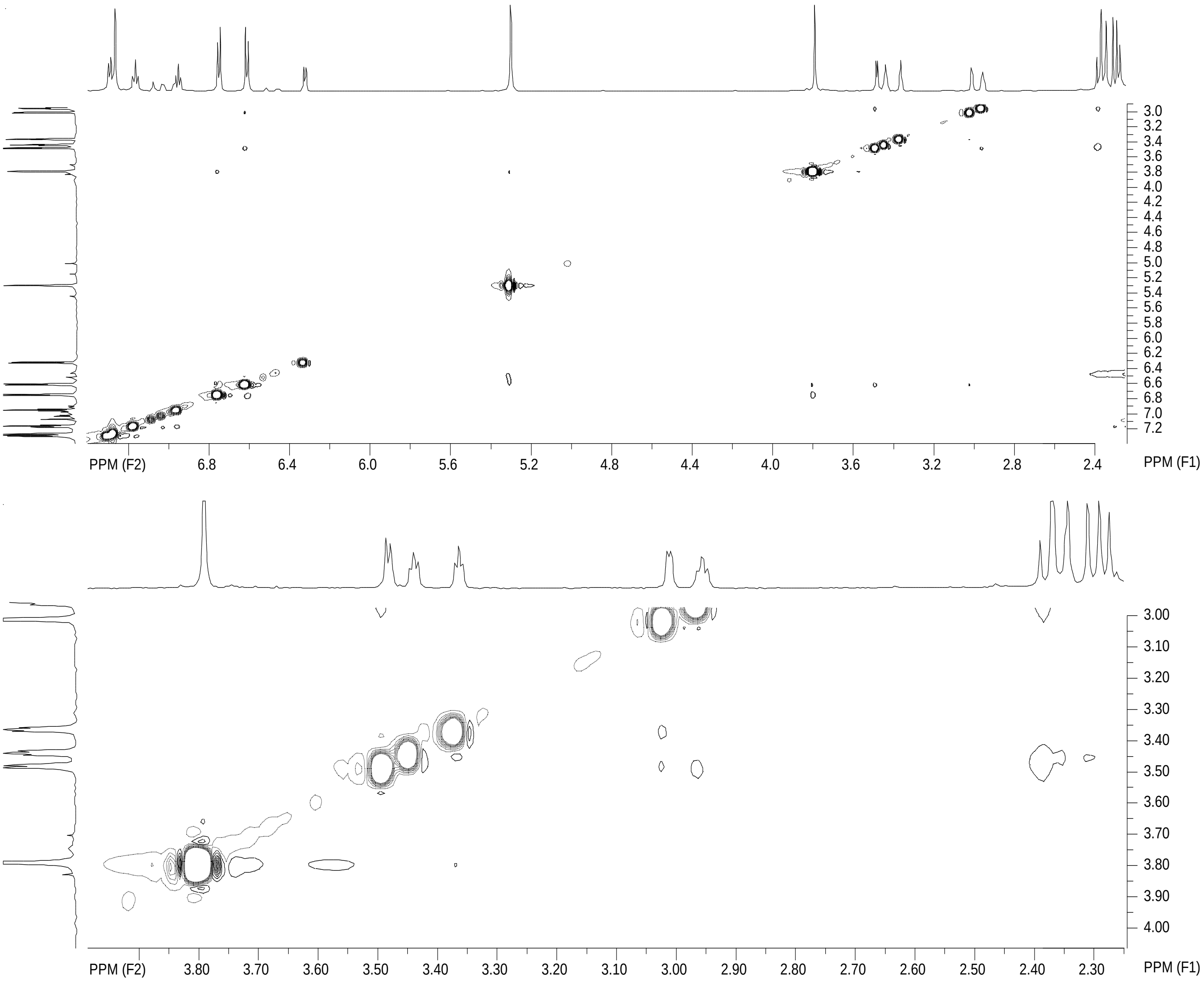

Figure S26. LR COSY spectrum (CDCl3) of *endo*-**47**and its aliphatic part.

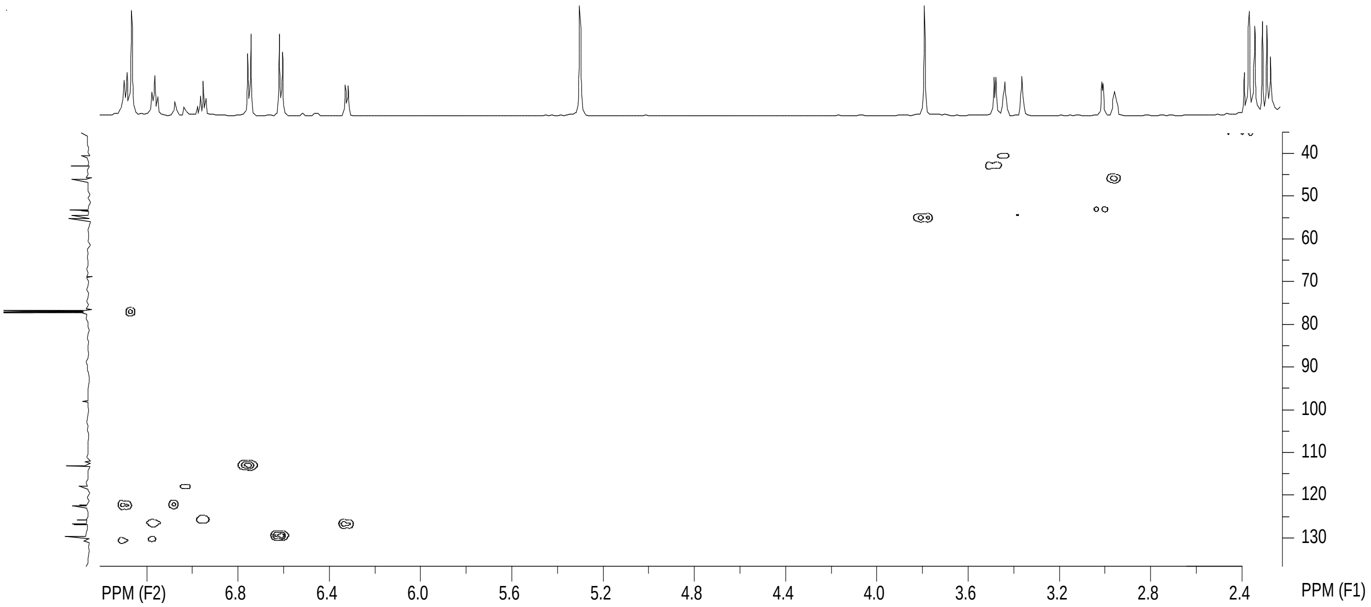

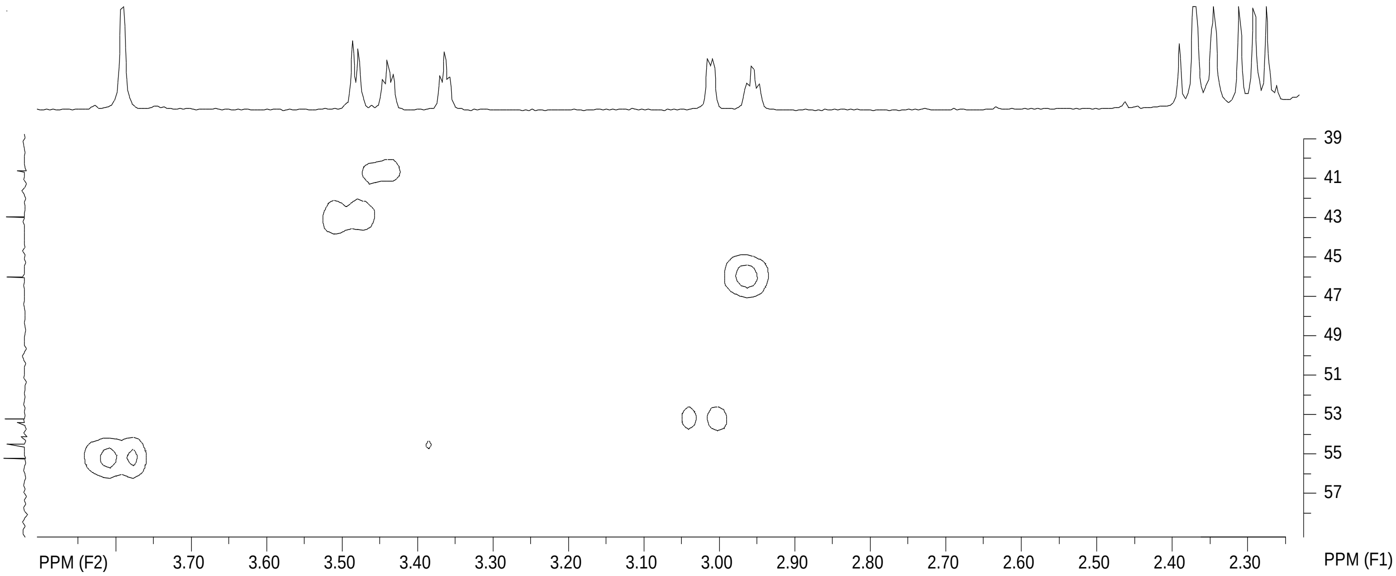

Figure S27. HSQC spectrum ($CDCl_3$) of *endo*-**47**and its aliphatic part.

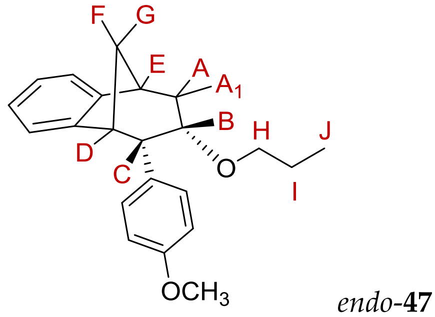

endo-**47**

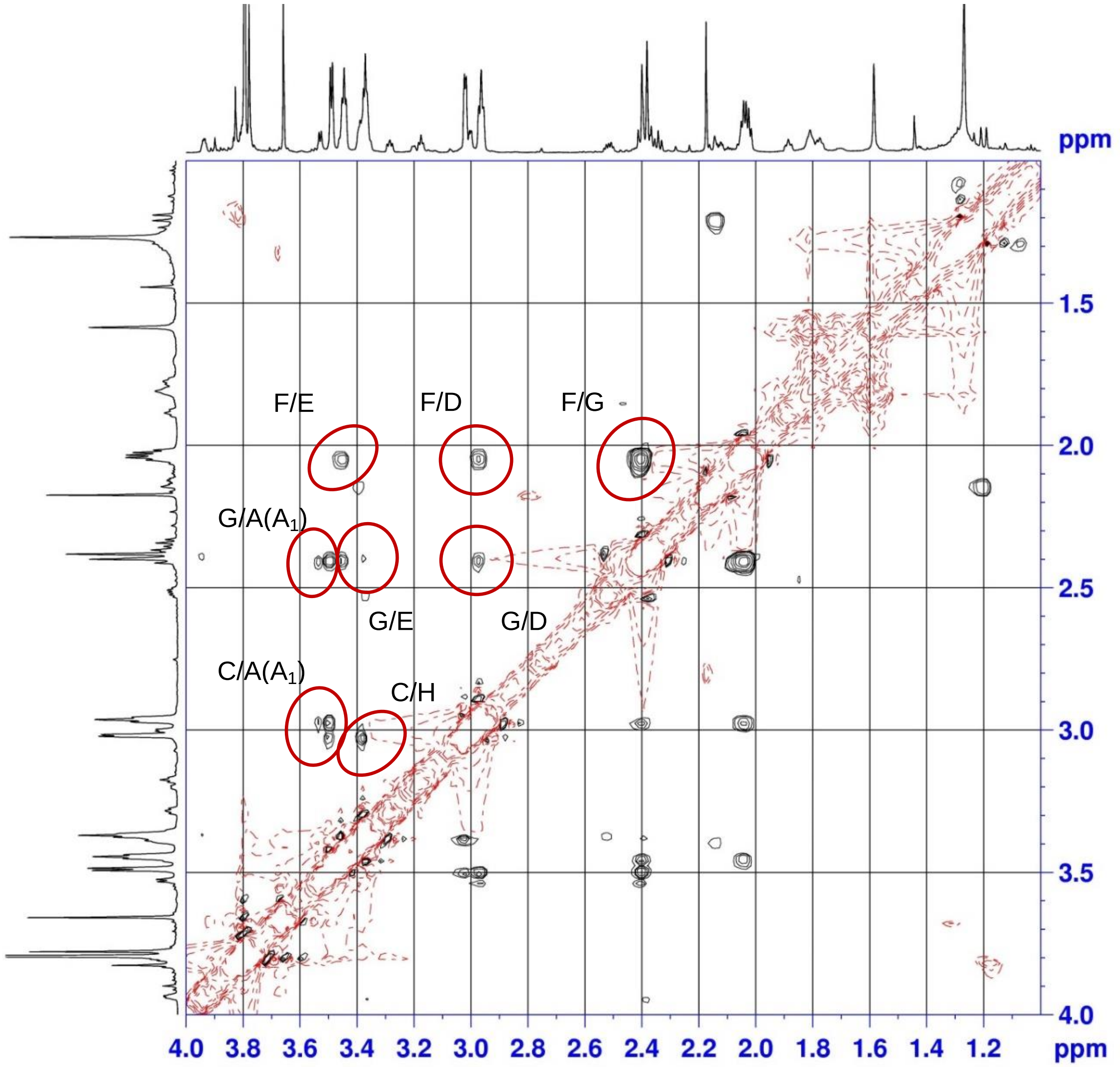

Figure S28. NOESY spectrum ($CDCl_3$) of *endo*-**47**.

(4R,9R)-9,10-dihydro-4H-4,9-methanobenzo[4,5]cyclohepta[1,2-*b*]furan (49)

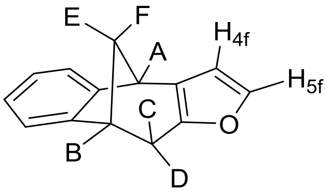

49

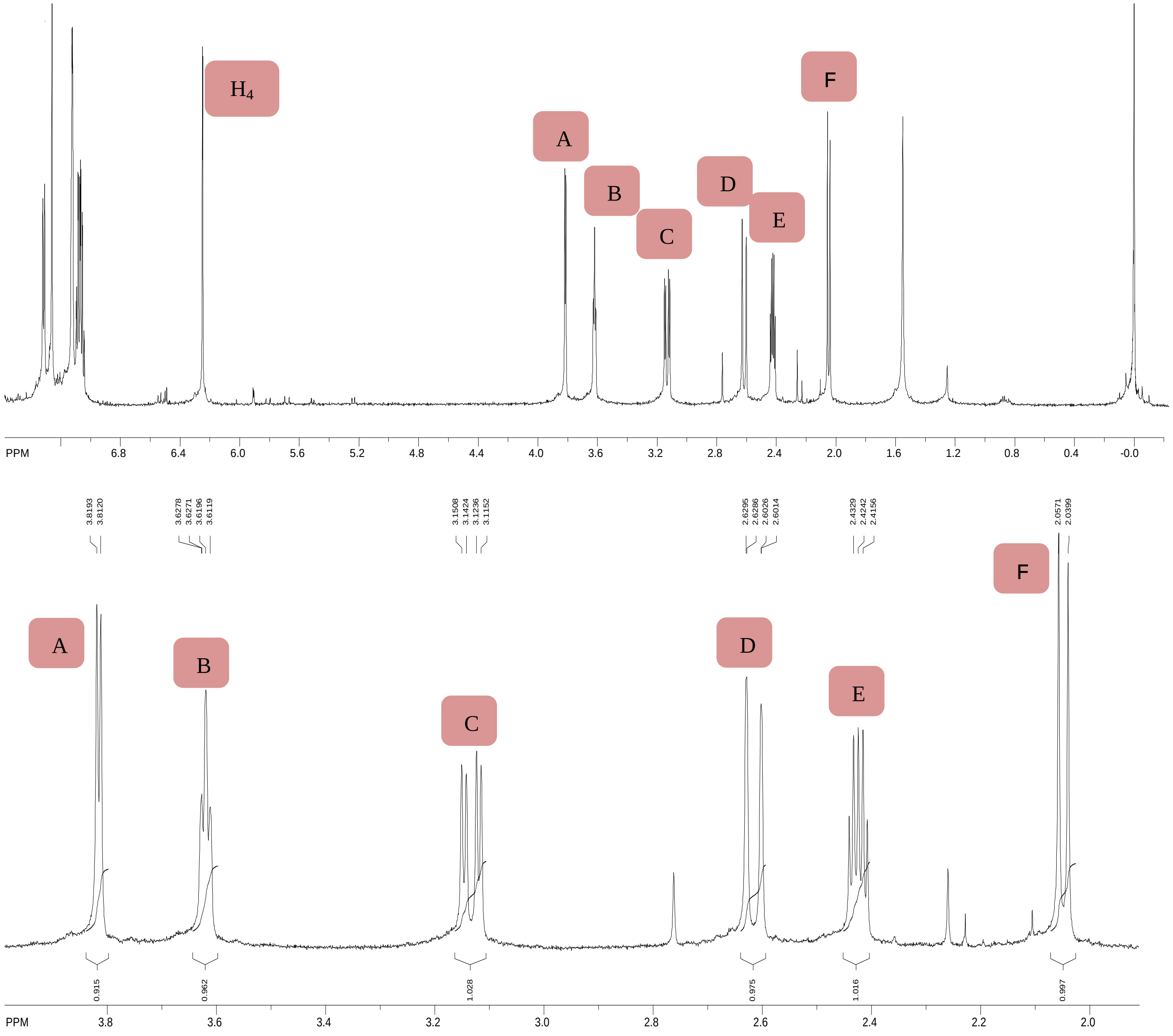

Figure S29. ^{1}H NMR spectrum ($CDCl_3$) of **49** and its aliphatic part.

(5*R*,9*R*,*Z*)-6-((*Z*)-3-hydroxybut-2-ene-1-ilydene)-6,7,8,9-tetrahydro-5*H*-5,9-methano-benzo[7]annulen-7-ol (51)

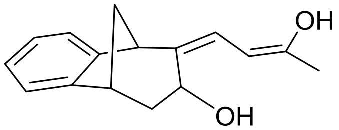

51

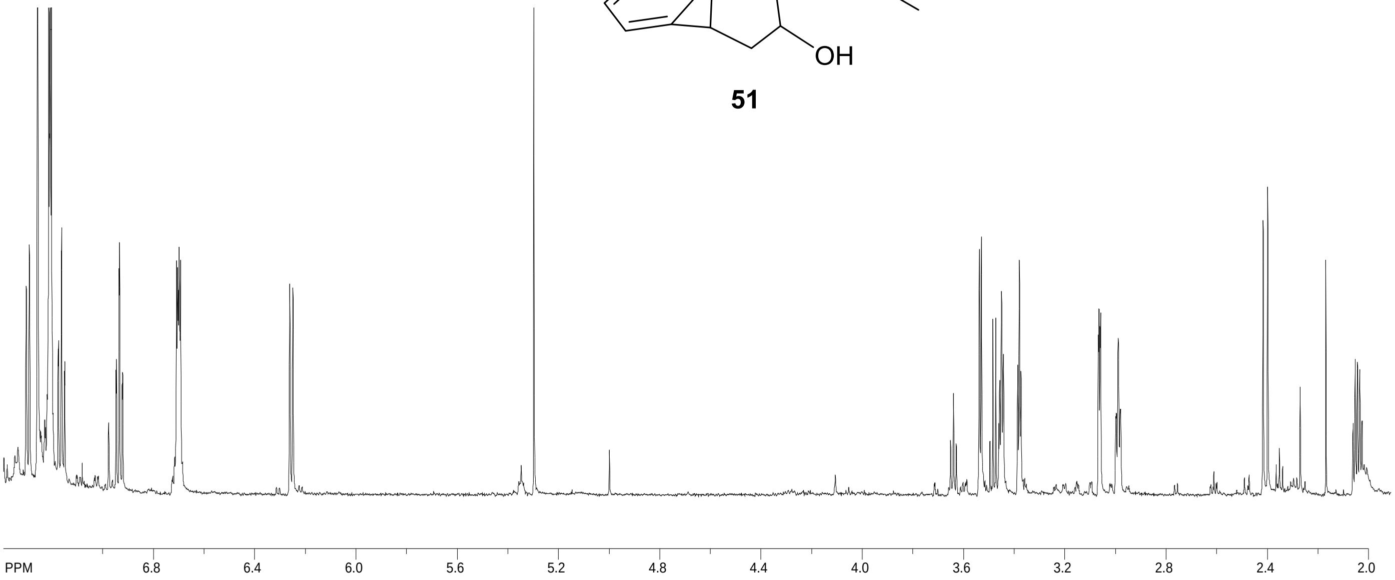

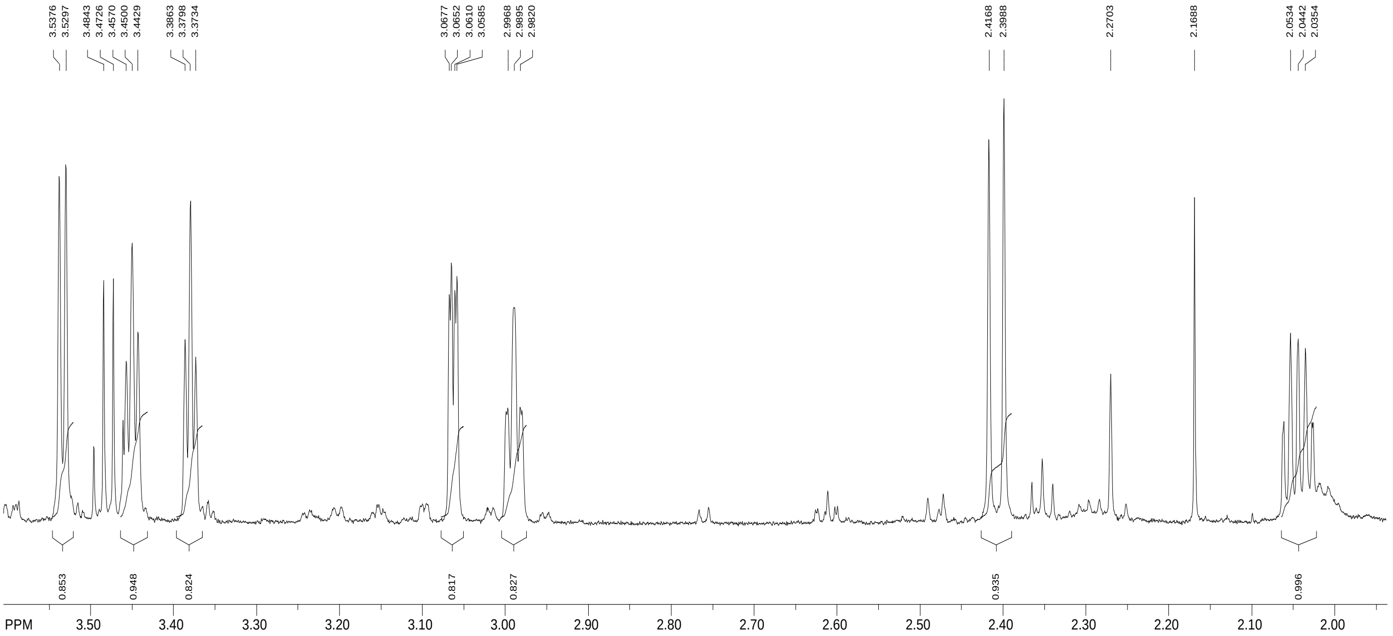

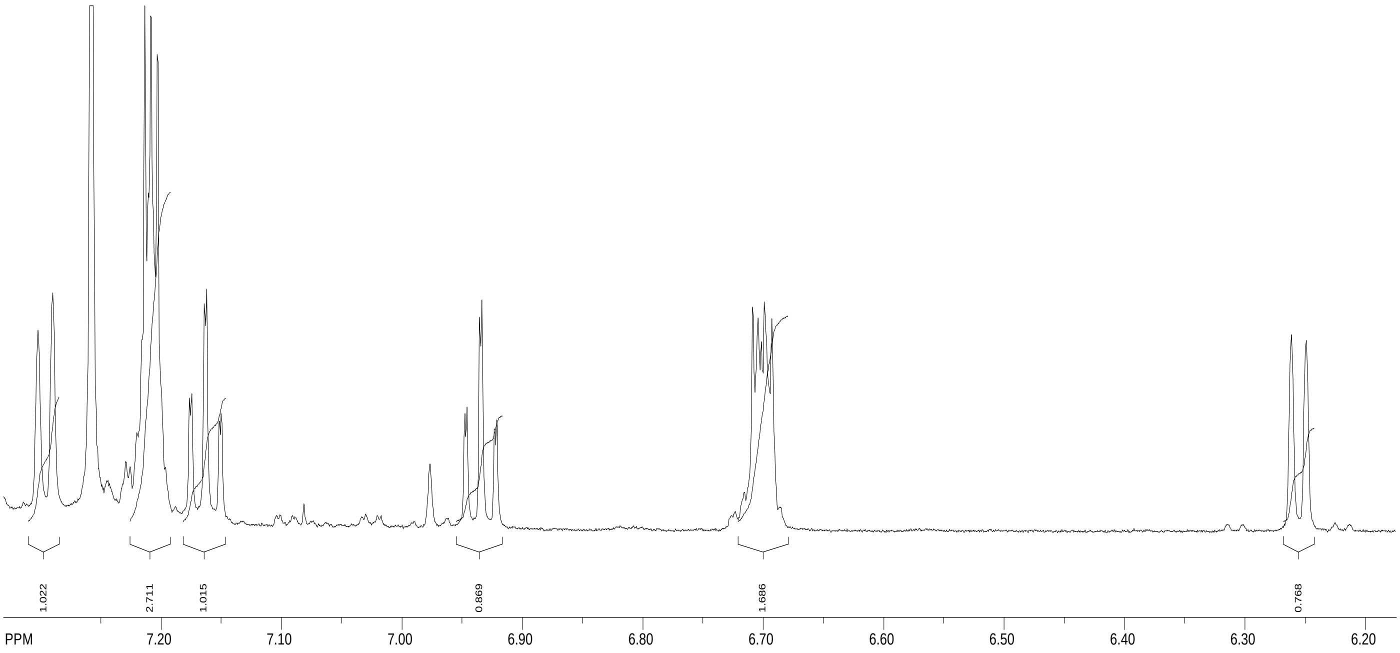

Figure S30. 1H NMR spectrum (CDCl3) of **51** its aliphatic part (previous page) and aromatic part.

(*Z*)-1-((5*R*,9*R*)-7-hydroxy-5,9-dihydro-6*H*-5,9-methanobenzo[7]annulen-6-ilydene)-pentan-3-one (53)

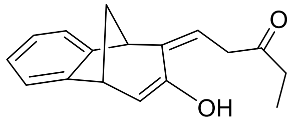

53

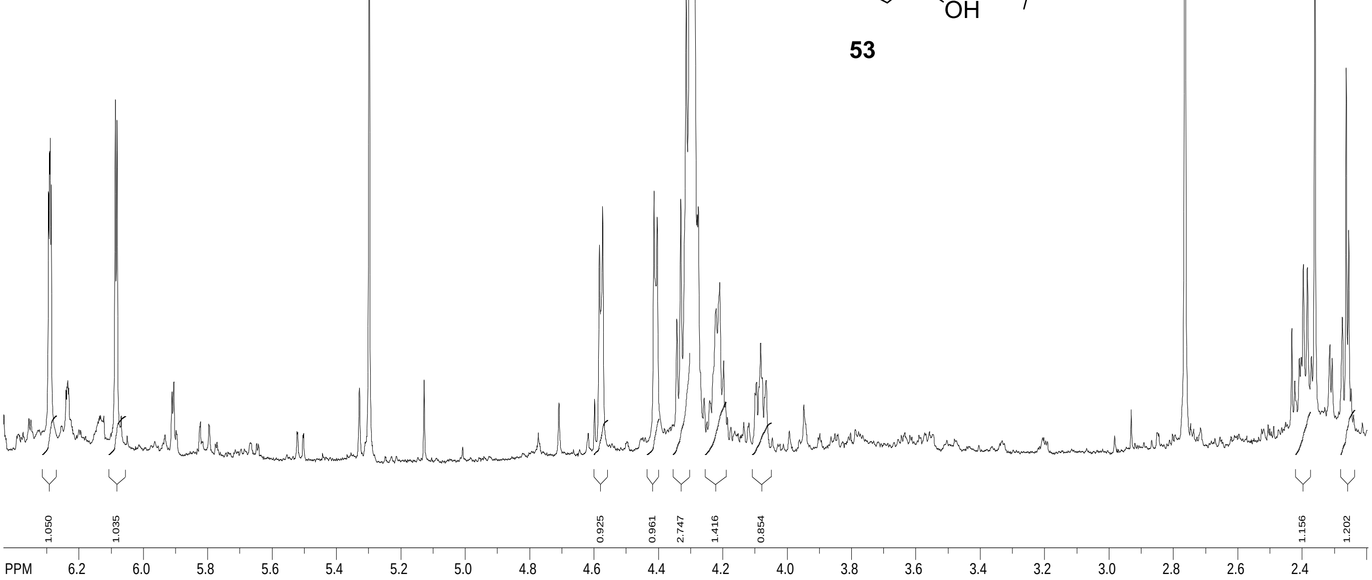

Figure S31. ^{1}H NMR spectrum ($CDCl_3$) of **53.**

Cartesian coordinates of optimized geometries of reactants, transition states, and products of reactions 1-4

***endo*-38-above**

scf done: -770.438741

C	0.817127	2.107451	0.408551
C	1.153954	0.767930	0.562235
C	2.301825	0.264219	-0.071488
C	3.099092	1.068898	-0.870672
C	2.755438	2.416119	-1.020791
C	1.628164	2.928157	-0.384328
C	0.492764	-0.352575	1.360019
C	1.698872	-1.287753	1.595968
C	2.409528	-1.216936	0.235346
C	1.548933	-1.902762	-0.812836
C	0.085574	-1.812377	-0.675670
C	-0.565531	-1.148572	0.544180
H	2.350562	-0.879793	2.374313
H	1.405360	-2.304109	1.869576
H	0.052709	-0.004520	2.297750
H	3.430835	-1.607249	0.235672
H	-0.052672	2.532650	0.899720
H	1.375784	3.978558	-0.497732
H	3.375537	3.066885	-1.630505
H	3.982762	0.665658	-1.358905
H	-0.879381	-1.979585	1.192872
C	-1.833599	-0.374521	0.197741
C	-2.725569	-0.850790	-0.772870
C	-3.913900	-0.181468	-1.054398
C	-4.248065	0.980503	-0.363223
C	-3.388835	1.450294	0.625020
C	-2.200840	0.776766	0.902545
H	-2.507574	-1.767640	-1.312513
H	-4.582781	-0.575195	-1.814321
H	-5.172260	1.506015	-0.584370
H	-3.640529	2.344101	1.188609
H	-1.564982	1.156551	1.694180
O	0.786198	-3.036256	-0.427533
H	1.973278	-1.959540	-1.815745
H	-0.496356	-1.831574	-1.593752

Complex-reactants (Reactions 1 and 2)

scf done: -771.056048

C	-2.515179	1.893112	0.477781
C	-2.037616	0.740336	-0.127978
C	-0.828331	0.748298	-0.837027
H	3.134284	1.325779	2.147547
C	-0.576178	3.085468	-0.360803
C	-1.776455	3.073784	0.347176
H	-0.484815	-0.299999	4.026384
C	-2.577734	-0.678637	-0.125901
C	-1.966691	-1.229774	-1.428183
C	-0.539775	-0.648841	-1.351308
C	0.290306	-1.479440	-0.320317
C	1.610730	-0.796616	-0.032159
C	1.795200	0.031088	1.081050
C	3.007422	0.692741	1.273851
C	4.046669	0.547453	0.358098
C	3.869657	-0.270227	-0.757045
C	2.661580	-0.935782	-0.945549
C	-0.510770	-1.760627	0.947931
O	-1.547043	-2.744570	0.858919
C	-1.928077	-1.386373	1.055983
H	-2.308112	-1.151043	2.049793
H	-3.668498	-0.746384	-0.078312
H	0.050750	-1.816933	1.879563
H	-1.987426	-2.320679	-1.476785
H	-2.498751	-0.818796	-2.291931
H	0.501603	-2.447709	-0.794244
H	2.527631	-1.577792	-1.814498
H	-0.009314	-0.646391	-2.308806
H	4.673720	-0.395417	-1.477085
H	4.989410	1.064464	0.513139
H	0.999108	0.161552	1.813395
H	0.856641	1.925545	-1.493518
H	-0.013927	4.010558	-0.451468
H	-2.141065	3.989463	0.803953
H	-3.445507	1.883964	1.039633
C	-0.087503	1.916583	-0.953234

Transition state (Reaction 1)

scf done: -771.012054

C	-1.978213	0.897708	-0.691037
C	-1.881341	-0.354756	-0.070799
C	-3.044692	-0.894055	0.494807
C	-4.263832	-0.221822	0.441845
C	-4.343902	1.021145	-0.180526
C	-3.196011	1.574169	-0.744856
C	-0.585662	-1.128690	0.117243
C	0.341755	-0.499295	1.208594
C	1.490263	-1.495471	1.483385
C	2.361703	-1.275679	0.235221
C	1.621547	-1.887308	-0.976588
C	0.217727	-1.523497	-1.127961
C	2.328577	0.235178	0.133246
C	1.131515	0.695324	0.708636
C	3.219228	1.123479	-0.447542
C	2.914211	2.490013	-0.436320
C	1.736923	2.947956	0.148550
C	0.832698	2.048136	0.727545
O	0.935710	-3.065390	-0.725347
H	-0.299887	-1.798227	-2.031827
H	2.036693	-1.206435	2.388101
H	1.152262	-2.529551	1.569119
H	-0.264574	-0.273072	2.092004
H	3.369110	-1.697274	0.318162
H	-0.089321	2.409118	1.177313
H	1.518699	4.012356	0.159167
H	3.606402	3.200200	-0.880603
H	4.140922	0.769356	-0.903446
H	-0.895529	-2.085787	0.551317
H	-1.092658	1.309957	-1.164140
H	-3.246448	2.542720	-1.235593
H	-5.290740	1.552175	-0.227470
H	-5.148312	-0.671268	0.885154
H	-2.992065	-1.862816	0.987535
H	0.356423	0.187939	-2.134110
H	2.238381	-1.851494	-1.888557

Product (Reaction 1)

scf done: -771.129131

C	0.561611	2.073747	0.768364
C	1.006578	0.761734	0.761152
C	2.240284	0.418630	0.171178
C	3.016767	1.400100	-0.431411
C	2.570629	2.729361	-0.419369
C	1.359800	3.065949	0.178580
C	0.338574	-0.515656	1.237436
C	1.569944	-1.409818	1.490314
C	2.423972	-1.070916	0.261294
C	1.749780	-1.859099	-0.965925
C	0.349251	-1.200325	-1.213843
C	-0.488492	-1.157620	0.074528
C	-1.843030	-0.496382	-0.071471
C	-2.037635	0.667623	-0.825439
C	-3.291242	1.273016	-0.902845
C	-4.382042	0.724278	-0.232289
C	-4.206432	-0.435907	0.519724
C	-2.950774	-1.033231	0.596558
O	1.686405	-3.158751	-0.744069
H	-0.171029	-1.811273	-1.962186
H	2.062036	-1.127579	2.430269
H	1.336492	-2.477551	1.490680
H	-0.302629	-0.373404	2.115021
H	3.467181	-1.396216	0.337510
H	-0.391268	2.335344	1.223910
H	1.031778	4.101876	0.190647
H	3.180091	3.505613	-0.875206
H	3.965619	1.145810	-0.899103
H	-0.657501	-2.200398	0.371116
H	-1.201157	1.109304	-1.359133
H	-3.415465	2.176059	-1.494827
H	-5.359699	1.193175	-0.299371
H	-5.049199	-0.880260	1.042533
H	-2.820731	-1.940779	1.183138
H	0.454986	-0.190171	-1.631939
H	2.378245	-1.536541	-1.858396

Transition state (Reaction 2)

scf done: -771.011371

C	-0.065163	1.936482	-1.050895
C	-0.796860	0.782289	-0.795854
C	-1.903260	0.811132	0.057374

C	-2.319541	2.005575	0.631348
C	-1.606020	3.172532	0.352281
C	-0.481017	3.138598	-0.474865
C	-0.629217	-0.624358	-1.335234
C	-2.100446	-1.097179	-1.274028
C	-2.491645	-0.593009	0.139680
C	-1.774565	-1.453188	1.190162
C	-0.504957	-2.073103	0.803498
C	0.266780	-1.518629	-0.416259
C	1.556178	-0.815945	-0.061688
C	1.661076	-0.053045	1.107440
C	2.837494	0.632355	1.403102
C	3.926436	0.570685	0.535065
C	3.831104	-0.182200	-0.634007
C	2.654863	-0.871084	-0.924932
O	-1.451621	-3.019423	0.452335
H	-2.686260	-0.556784	-2.026022
H	-2.226179	-2.169861	-1.395361
H	-0.208484	-0.627717	-2.346928
H	-3.570105	-0.591013	0.323164
H	0.810037	1.907441	-1.696775
H	0.068484	4.054050	-0.676628
H	-1.926223	4.116092	0.786134
H	-3.177865	2.028598	1.297635
H	0.514618	-2.414977	-1.001989
H	0.801944	0.016601	1.777203
H	2.900568	1.221743	2.313865
H	4.842364	1.107129	0.766528
H	4.674294	-0.238048	-1.317366
H	2.586039	-1.462281	-1.836451
H	-2.289564	-1.842540	2.048252
H	0.184474	-2.349836	1.618539
H	-1.431341	-0.081801	2.524214

Product (Reaction 2)

scf done: -771.117263

C	-0.030074	1.925087	-1.159057
C	-0.832860	0.831444	-0.860775
C	-1.865731	0.940610	0.077529
C	-2.096045	2.140896	0.738344
C	-1.295621	3.247115	0.430938
C	-0.273249	3.140850	-0.511481
C	-0.775882	-0.596367	-1.363024
C	-2.242903	-1.038072	-1.154693
C	-2.524020	-0.415596	0.232931
C	-1.766594	-1.231881	1.314686
C	-0.672099	-2.200531	0.720800
C	0.142921	-1.504125	-0.473677
C	1.432329	-0.820303	-0.098973
C	1.524015	0.032467	1.011797
C	2.718558	0.668437	1.338568
C	3.859387	0.467151	0.562174
C	3.790542	-0.380253	-0.541183
C	2.590856	-1.015351	-0.860516
O	-1.209408	-3.344412	0.309560
H	-2.891113	-0.591882	-1.918604
H	-2.333907	-2.125531	-1.126977
H	-0.442676	-0.644768	-2.406452
H	-3.591534	-0.334768	0.466442
H	0.781667	1.837786	-1.878504
H	0.339560	4.008046	-0.742307
H	-1.473318	4.197020	0.928186
H	-2.889720	2.227011	1.477575
H	0.397092	-2.377261	-1.084947
H	0.640355	0.210721	1.620255
H	2.758540	1.325602	2.203398
H	4.791461	0.964108	0.816369
H	4.672284	-0.551328	-1.153650
H	2.547424	-1.677511	-1.723358
H	-2.458656	-1.912125	1.822987
H	0.094653	-2.303720	1.551166
H	-1.369857	-0.541739	2.074773

***endo*-38-below**

scf done: -770.440777

C	-2.920276	-0.828049	0.795525
C	-1.748906	-0.607937	0.063972
C	-1.736338	0.421715	-0.885092
C	-2.870553	1.203111	-1.095672
C	-4.032317	0.973059	-0.361409
C	-4.054326	-0.046580	0.587608
C	-0.523729	-1.452937	0.342905
C	0.465028	-0.781272	1.345868
C	1.742637	-1.645366	1.411960
C	2.434301	-1.229616	0.095243
C	1.639250	-1.872625	-1.050509
C	0.171895	-1.956866	-0.916643
C	2.204827	0.269246	0.116110
C	1.033199	0.529265	0.835829
C	2.925094	1.298375	-0.469237
C	2.464687	2.610324	-0.312524
C	1.299699	2.872785	0.406491
C	0.569781	1.828958	0.985267
H	2.154702	-2.626632	-1.643164
H	2.353428	-1.367107	2.276240
H	1.529749	-2.718947	1.457396
H	-0.045923	-0.674359	2.307849
H	3.487371	-1.513570	0.032251
H	-0.340134	2.035847	1.543298
H	0.957740	3.897338	0.521839
H	3.021469	3.432394	-0.753446
H	3.831922	1.095216	-1.033222
H	-0.867510	-2.365934	0.847814
H	-0.832353	0.608996	-1.455853
H	-2.844377	1.996303	-1.837783
H	-4.915386	1.582751	-0.529936
H	-4.955633	-0.238232	1.163111
H	-2.942751	-1.625914	1.535256
O	0.781875	-1.024932	-1.805957
H	-0.356594	-2.758125	-1.433344

Reactants (Reactions 3 and 4)

scf done: -771.052798

C	-0.480397	1.897986	-0.967955
C	-0.993151	0.616160	-0.824625
C	-2.172907	0.395579	-0.103007
C	-2.849928	1.450877	0.488271
C	-2.340150	2.745508	0.337472
C	-1.167666	2.967377	-0.382495
C	-0.477380	-0.713075	-1.344514
C	-1.786990	-1.526686	-1.410712
C	-2.458656	-1.092714	-0.092598
C	-1.686050	-1.772455	1.046885
C	-0.225018	-1.909926	0.910907
C	0.486941	-1.428795	-0.348355
C	1.747236	-0.635876	-0.075645
C	1.786839	0.384942	0.882307
C	2.952943	1.120297	1.085355
C	4.096164	0.853020	0.334221
C	4.067056	-0.158830	-0.623009
C	2.901074	-0.893941	-0.823184
O	-0.793607	-0.958131	1.807696
H	-2.230071	-2.521072	1.617251
H	-2.389782	-1.220257	-2.271784
H	-1.633287	-2.609241	-1.448899
H	0.036720	-0.618102	-2.306635
H	-3.515384	-1.358696	-0.031856
H	0.436005	2.072597	-1.526964
H	-0.786456	3.978602	-0.493779
H	-2.864745	3.585901	0.783786
H	-3.763425	1.280077	1.052423
H	0.788504	-2.353071	-0.859360
H	0.897418	0.597896	1.467008
H	2.967190	1.907011	1.834917
H	5.004257	1.426732	0.496618
H	4.952987	-0.380241	-1.211662
H	2.883575	-1.686021	-1.569274
H	0.273091	-2.735146	1.420095
H	-3.538998	-4.196982	0.064662

Transition state (Reaction 3)

scf done: -771.012435

C	1.744852	0.212832	1.029221
C	1.697052	-0.675580	-0.052282
C	2.806715	-0.739359	-0.904892
C	3.933887	0.050470	-0.689158
C	3.973211	0.922955	0.396375
C	2.875457	0.997066	1.251694
C	0.484700	-1.524341	-0.396351
C	-0.494567	-0.771478	-1.358096
C	-1.827730	-1.552207	-1.406227
C	-2.452250	-1.105176	-0.067449
C	-1.625581	-1.740099	1.098196
C	-0.266956	-2.173195	0.768167
C	-2.157706	0.380196	-0.109943
C	-0.974625	0.569683	-0.834982
C	-2.819366	1.457280	0.456469
C	-2.289212	2.742051	0.284383
C	-1.109003	2.931758	-0.430926
C	-0.438974	1.839137	-0.995538
O	-0.844205	-0.859542	1.821437
H	-2.228076	-2.452943	1.682500
H	-2.437973	-1.212801	-2.250997
H	-1.682379	-2.633549	-1.466580
H	-0.001894	-0.676776	-2.331880
H	-3.515885	-1.341141	0.029106
H	0.484038	1.988310	-1.552120

H	-0.708266	3.933949	-0.556205
H	-2.801938	3.598750	0.713902
H	-3.735994	1.313080	1.023943
H	0.865265	-2.361353	-0.985215
H	0.881100	0.275991	1.681333
H	2.895719	1.676515	2.099931
H	4.851511	1.537223	0.575169
H	4.782129	-0.021291	-1.364766
H	2.785527	-1.424821	-1.750394
H	0.287567	-2.717892	1.515354
H	-0.449642	-3.900431	-0.002929

Product (Reaction 3)
scf done: -771.121481

C	1.380902	-0.334760	1.094144
C	1.376075	-1.028498	-0.124179
C	2.532247	-1.027769	-0.909205
C	3.678659	-0.347596	-0.498190
C	3.679443	0.340183	0.712811
C	2.528641	0.341417	1.503492
C	0.117912	-1.705130	-0.625287
C	-0.706195	-0.747786	-1.566292
C	-2.208961	-1.084210	-1.428821
C	-2.506485	-0.475295	-0.041101
C	-1.890352	-1.367284	1.136351
C	-0.775431	-2.290656	0.499634
C	-1.736065	0.814798	-0.122311
C	-0.695571	0.674210	-1.044272
C	-1.880707	1.998499	0.590472
C	-1.000547	3.054891	0.338123
C	0.021800	2.919731	-0.602434
C	0.187369	1.716815	-1.295153
O	-1.516764	-0.663236	2.194990
H	-2.723591	-2.110146	1.347058
H	-2.778924	-0.581322	-2.220318
H	-2.420187	-2.157169	-1.488916
H	-0.320162	-0.822969	-2.588881
H	-3.572253	-0.309772	0.153623
H	1.003365	1.597699	-2.005261
H	0.697332	3.750268	-0.790030
H	-1.110414	3.990596	0.880785
H	-2.666751	2.099861	1.333624
H	0.438859	-2.542917	-1.259896
H	0.455086	-0.326169	1.687389
H	2.524836	0.882217	2.446939
H	4.569773	0.871653	1.038634
H	4.569437	-0.358991	-1.120971
H	2.534902	-1.563835	-1.857347
H	-0.146516	-2.631513	1.330829
H	-1.253575	-3.189517	0.087232

Transition state (Reaction 4)
scf done: -771.017101

C	-0.741939	1.881180	-0.901171
C	-1.082097	0.543068	-0.787882
C	-2.273020	0.160198	-0.150143
C	-3.134898	1.110024	0.368111
C	-2.795506	2.465341	0.250479
C	-1.611420	2.846982	-0.373333
C	-0.376305	-0.704412	-1.291598
C	-1.584123	-1.646318	-1.469276
C	-2.357775	-1.359436	-0.163966
C	-1.569422	-1.966750	1.006526
C	-0.128805	-1.717643	1.066704
C	0.584823	-1.359579	-0.253403
C	1.836837	-0.538465	-0.037581
C	1.871789	0.547298	0.848650
C	3.035806	1.298911	1.001543
C	4.181562	0.988770	0.270868
C	4.157053	-0.085465	-0.616433
C	2.995027	-0.838504	-0.764724
O	-0.681930	-0.668625	1.781434
H	-2.065831	-2.338844	1.885643
H	-2.178692	-1.346096	-2.339595
H	-1.305435	-2.697246	-1.559180
H	0.181839	-0.523616	-2.217447
H	-3.379549	-1.743261	-0.159632
H	0.179437	2.182443	-1.394841
H	-1.363243	3.901272	-0.461594
H	-3.466109	3.223976	0.645233
H	-4.059137	0.816313	0.860275
H	0.893952	-2.312293	-0.705782
H	0.978603	0.776380	1.421536
H	3.047349	2.133103	1.698250
H	5.086855	1.576994	0.393474
H	5.044062	-0.341134	-1.189700
H	2.983210	-1.680175	-1.454493
H	0.482772	-2.429029	1.646816
H	-1.834386	-3.794512	0.419596

Product (Reaction 4)
scf done: -771.121440

C	-0.972992	1.830361	-0.908685
C	-1.173076	0.466508	-0.775248
C	-2.328529	-0.029185	-0.149395
C	-3.285236	0.836270	0.352683
C	-3.075654	2.217708	0.236926
C	-1.933087	2.709432	-0.387038
C	-0.334536	-0.708818	-1.240993
C	-1.444319	-1.758387	-1.463028
C	-2.292818	-1.546398	-0.193127
C	-1.510353	-2.143019	0.995832
C	-0.138756	-1.420847	1.270180
C	0.614759	-1.258210	-0.132933
C	1.880492	-0.448013	-0.022875
C	1.887393	0.823299	0.575770
C	3.060725	1.572956	0.632543
C	4.250681	1.078147	0.097373
C	4.259035	-0.183591	-0.492289
C	3.083998	-0.933324	-0.549495
O	-0.291043	-0.303808	1.963662
H	-2.083414	-2.035394	1.924217
H	-2.022715	-1.525723	-2.365406
H	-1.054035	-2.778786	-1.549249
H	0.238434	-0.483806	-2.149098
H	-3.294632	-1.987019	-0.265015
H	-0.083962	2.217509	-1.401736
H	-1.791294	3.782641	-0.484825
H	-3.818695	2.910483	0.623265
H	-4.186384	0.455602	0.828804
H	0.907143	-2.262182	-0.481270
H	0.962467	1.147885	1.040166
H	3.049078	2.552266	1.105792
H	5.162313	1.668143	0.143068
H	5.179439	-0.586804	-0.907182
H	3.100058	-1.918264	-1.013477
H	0.492908	-2.214553	1.788511
H	-1.381768	-3.220628	0.801604
